# Satellite-Like W-Elements: Repetitive, Transcribed, and Putative Mobile Genetic Factors with Potential Roles for Biology and Evolution of *Schistosoma mansoni*

**DOI:** 10.1093/gbe/evab204

**Published:** 2021-09-01

**Authors:** Maria Stitz, Cristian Chaparro, Zhigang Lu, V Janett Olzog, Christina E Weinberg, Jochen Blom, Alexander Goesmann, Christoph Grunau, Christoph G Grevelding

**Affiliations:** 1 Institute of Parasitology, BFS, Justus Liebig University Giessen, Giessen, Germany; 2 IHPE, CNRS, IFREMER, UPVD, University Montpellier, Perpignan, France; 3 Institute for Biochemistry, Leipzig University, Germany; 4 Bioinformatics and Systems Biology, Justus Liebig University Giessen, Germany

**Keywords:** *Schistosoma mansoni*, genome evolution, W-element, mobile genetic element, noncoding RNA, ribozyme

## Abstract

A large portion of animal and plant genomes consists of noncoding DNA. This part includes tandemly repeated sequences and gained attention because it offers exciting insights into genome biology. We investigated satellite-DNA elements of the platyhelminth *Schistosoma mansoni*, a parasite with remarkable biological features. *Schistosoma mansoni* lives in the vasculature of humans causing schistosomiasis, a disease of worldwide importance. Schistosomes are the only trematodes that have evolved separate sexes, and the sexual maturation of the female depends on constant pairing with the male. The schistosome karyotype comprises eight chromosome pairs, males are homogametic (ZZ) and females are heterogametic (ZW). Part of the repetitive DNA of *S. mansoni* are W-elements (WEs), originally discovered as female-specific satellite DNAs in the heterochromatic block of the W-chromosome. Based on new genome and transcriptome data, we performed a reanalysis of the W-element families (WEFs). Besides a new classification of 19 WEFs, we provide first evidence for stage-, sex-, pairing-, gonad-, and strain-specific/preferential transcription of WEs as well as their mobile nature, deduced from autosomal copies of full-length and partial WEs. Structural analyses suggested roles as sources of noncoding RNA-like hammerhead ribozymes, for which we obtained functional evidence. Finally, the variable WEF occurrence in different schistosome species revealed remarkable divergence. From these results, we propose that WEs potentially exert enduring influence on the biology of *S. mansoni*. Their variable occurrence in different strains, isolates, and species suggests that schistosome WEs may represent genetic factors taking effect on variability and evolution of the family Schistosomatidae.

**Significance**

Previous studies described W-elements (WEs) as repeated, noncoding satellite-DNA of the large heterochromatic part of the female-specific W-chromosome of *S. mansoni* with unknown function. We challenge this view by analyzing all W-element families (WEFs), their structures, WEF-dependent transcript profiles (including stage-, sex-, pairing-, gonad-, and strain-specific/preferential expression), and autosomal occurrence, which indicates their mobile nature. Furthermore, we predicted roles of WEs as carriers of genetic information such as noncoding RNA, for which obtained biochemical evidence. Analyzing different schistosome strains, isolates, and even species finally showed the variable existence of WEFs. These finding suggest that WEs might play roles for the biology of *S. mansoni* and might represent one of the driving forces in the evolution of the family Schistosomatidae.

## Introduction

Schistosomes are parasitic plathyhelminths and cause schistosomiasis (bilharzia). Listed as neglected tropical disease by the WHO, schistosomiasis ranks close to malaria in terms of parasite-induced human morbidity and mortality (Hotez and Kamatha 2009; Colley et al. 2014). The life cycle of the parasite is complex with a fresh-water snail of the genus *Biomphalaria* as intermediate host, producing human-dwelling larvae (cercariae) that develop in the vertebrate host into adults, pair, and produce eggs that are excreted with the feces and hatch snail-infecting miracidia when in contact with water.

Schistosomes are evolutionary unique as they are the only digenean parasites that have evolved separate sexes (Moné and Boissier 2004). Heteromorphic sex chromosomes determine the sex of schistosomes, with ZZ in the males and ZW in the females. Earlier studies from us and others has shown that the females-specific region of the W-chromosome is essentially composed of repetitive DNA sequences (W-elements, WEs) arranged in large satellite blocks (Lepesant, Cosseau, et al. 2012; Lepesant, Roquis, et al. 2012). WE sequences are abundant on the W-chromosome, but may occasionally be transmitted to autosomes, shown for the female W1 repeat sequence (Grevelding 1999). In most species with Y or W sex chromosomes, it is found that: 1) repetitive sequences accumulate on these chromosomes, 2) large regions are heterochromatic, and 3) these chromosomes deteriorate or are completely absent in an extreme case. Among the accepted evolutionary theories is that the accumulation of repetitive sequences on one of sex chromosomes has facilitated recombination suppression between the heterochromosomes thus protecting sexually beneficial loci (Ohno 1967). Another hypothesis is that chromosome rearrangements, sequence accumulation, and amplification may have occurred near the sex-determining locus as a result of suppression of recombination (Charlesworth 1991; Marais and Galtier 2003). Heterochromatization of the W-chromosome in schistosomes has long been known and has even been used as a marker for sex identification at the cercaria stage, where male and female phenotypes are indistinguishable (Grossman et al. 1980, 1981; Liberatos and Short 1983). Our earlier results (Lepesant, Cosseau, et al. 2012) showed that the repetitive sequences located in the heterochromatic region of the W-chromosome carry a euchromatic signature in the miracidia stage (presence of H3K9ac and H3K4me3), which is gradually lost during development into adults. In most species, the structural change of the chromatin is a highly organized process, and the observed heterochromatin/euchromatin cycle of W-specific repeats suggests functional importance. Full or partial euchromatization could not only allow transcription of functional WE but may also open space for mobilization of these elements. The WEs W1 and W2 were originally found in Puerto Rican isolates of *Schistosoma mansoni* (Spotila et al 1987; Webster et al. 1989; Drew and Brindley 1995). Southern blot analysis demonstrated their female-specific occurrence, and in situ hybridization localized W1 and W2 in the heterochromatic region of the W-chromosome (Hirai et al. 1989; Spotila et al. 1989). According to their repetitive nature, both elements were classified as satellite-like DNA (satDNA). Subsequent studies in the Liberian strain and further strains of *S*. *mansoni* demonstrated W1 and W2 occurrence in both sexes of nearly all investigated strains, including another isolate from Puerto Rico (Grevelding 1995; Quack et al. 1998). In contrast to the polymorphic occurrence of W1 and W2, another family of repetitive elements, called D9 (Spotila et al. 1989), repeatedly occurred in the investigated schistosome strains (Quack et al. 1998). Molecular analyses of clonal populations obtained from crossing experiments with characterized parental generations, including males with and without W1 and W2, showed the variable emergence of both WEs among 10 generations of male progeny—even when the parental male exhibited no W1 and W2 (Grevelding 1999). These data indicated a meiotic level of (illegitimate) recombination and suggested a Z-chromosomal or autosomal existence of W1 and W2 in the male progeny of these crosses. However, diversity in W1/W2 abundance and presence occurred also among siblings of single offspring generations. This pointed to an additional process generating variable copy numbers of WEs in the genome. Indeed, the analysis of clonal cercariae, generated by monomiracidial snail infections, demonstrated differences in W1 and W2 copy numbers within clonal cercariae obtained from the same snails but at different time points post infection (Grevelding 1999). This was unexpected because clonal cercariae were considered to be genetically identical (Jourdane and Theron 1980; Jones and Kusel 1989). Additionally, the genetic heterogeneity found in individuals of the same clonal cercarial population led to the hypothesis that mitotic recombination may contribute to variable WE copy numbers in males, because only asexual but no sexual reproduction processes take place in the intermediate host (Grevelding 1999). Using an in vitro technique that allowed the generation of defined clonal daughter sporocysts originating from a single mother finally confirmed that W1 copy numbers can vary among daughter sporocysts generated by one defined mother (Bayne and Grevelding 2003). These results clearly pointed to the possibility of mitotic recombination or transposition in the intermediate host. The underlying mechanisms of WE variability remained obscure.et al. 2012b

A microarray study with RNA of female cercariae provided first evidence for W1 and W2 transcripts (Fitzpatrick et al 2008). The authors hypothesized that these transcripts may be associated to germ-line protection in female schistosomes such as interfering with retrotransposable element activity, as previously proposed for repeat elements in *Drosophila* (Pélisson et al. 2007). In 2012, a comprehensive sequencing analysis to de novo assemble *S. mansoni* repeat elements based on version 5 (V5) of the genome (Berriman et al. 2009) predicted 36 W-element families (WEFs) (Lepesant, Roquis, et al. 2012).

In addition to their peculiar evolutionary position as the only dioeciously living trematodes, another unique feature of schistosome biology is the essentiality of a constant pairing contact for the sexual maturation of the female. Pairing induces mitotic activity and differentiation processes in the female that finally lead to the development of the female gonads, ovary, and vitellarium (Popiel and Basch 1984; Den Hollander and Erasmus 1985; Kunz 2001; Grevelding 2004). This is a prerequisite for egg production and closely associated with the pathology of schistosomiasis since eggs, which fail to reach the gut lumen, migrate via the blood stream to liver and spleen. Here, these eggs lodge in the tissues causing granuloma formation, inflammatory processes, and fibrosis (Olveda et al. 2014). The sexual maturation status of a female is reversible. Upon separation, egg production stops and females dedifferentiate to an immature status. Upon repairing, differentiation of the gonads and egg production start again (Clough 1981). Although the underlying processes have not been completely understood, recent transcriptomics approaches have highlighted a complex scenario of male–female interaction. A comparative RNA-Seq analysis of paired and unpaired adult *S. mansoni* and their gonads showed the occurrence of >7,000 gene transcripts in the gonads of both sexes, of which 243 (testes) and 3,600 (ovaries) were pairing-dependently transcribed. High numbers of differentially expressed genes in the ovary were expected because of the pairing-induced sexual maturation of females (Erasmus 1973; Shaw 1987; Kunz 2001; Neves et al., 2005; Beckmann et al., 2010). Among others, evidence was obtained for the participation of neuronal processes, guided by G protein-coupled receptors and neuropeptides, in male-associated processes of the male–female interaction (Hahnel et al. 2018; Lu et al. 2019). In addition, kinase signaling seems to dominate processes in females leading to gonad differentiation and the maintenance of the sexual maturation status (Grevelding et al. 2018).

In this study, we challenge the classical view that repetitive DNA on the sex chromosomes is simply a by-product of heterochromatization and provide further evidence for their functional importance. We made use of genome sequencing updates in combination with recently obtained transcriptome data sets to reanalyze WEFs, their structures, their chromosomal occurrence, their physical relationships, and their developmentally regulated and strain-associated transcriptional activities. From the obtained results, we propose a hypothesis for their functional roles in schistosome biology and evolution.

## Results

### There Are 19 WEFs in *S. mansoni*

To get a new overview of WEFs in *S. mansoni*, we performed a local BLAST (Basic Local Alignment Search Tool) search of the published WEFs (Lepesant, Roquis, et al. 2012), which were based on version 5 (V5) of the genome, against version 7 (V7; PRJEA36577, Puerto Rican strain; https://parasite.wormbase.org; last accessed September 2021; Howe et al. 2017). Except for W32, we found all 36 examined WEFs with a high percent identity on W-scaffolds, which cover DNA sequences, which are likely derived from/correspond to the female-specific sex chromosome W but have not been exactly assembled yet. We detected differences in DNA sequence similarity among individual WEs of a single WEF. For instance, the ten most significant hits of W6 elements (monomer repeat length: 310 bp) showed no mismatch and correspond 100%, whereas the ten most significant hits of W23 elements (monomer repeat length: 125 bp) showed deletions and mismatches resulting in 76–100% identity (supplementary fig. 1, Supplementary Material online). W1, W8, W10, W13, W17, W18, W20, W22, W31, W34, and W35 exclusively matched to W-scaffolds (table 1). Some WEs, or partial versions thereof, also occurred on autosomes. BLAST analyses revealed that some WEs of a single WEF clustered in single chromosomal regions, whereas other WEFs occurred as separate clusters in different chromosomal regions. For WEF 26 (W26.2), we detected clusters of different total lengths in two yet undefined regions of W, provisionally designated W003 and W010 in V7 (table 1). Although WEs of all 36 examined WEFs aligned to V7, repeat units of W9, W15, W19, and W32 showed no clustering of multiple, aligned repeats. Instead, we found a wide distribution of mostly partial fragments of these WEs on sex chromosomes and autosomes (data not shown). Because these WEs presented no multiple repeat character, we excluded them from further analysis.

**Table 1:** BLAST Results of WEFs in the Genome Version 7 of *Schistosoma mansoni*

No.	WE (V7)	WE (V5)	WE Monomer Lengths	Number of Copies	Scaffold	Start	Stop	Total Length of the Array
1	W1.2	W1; W23	475	270	SM_V7_W003	320,915	513,415	192,500
2	W2.2	W2; W3	709–711	Up to 34.1	SM_V7_W001	422,658	479,629	56,971
3	W4.2	W4; W30	1,206	11.7	SM_V7_W004	1	14,111	14,110
4	W5.2	W5	1,104	41.2	SM_V7_W018	1	45,515	45,514
5	W6.2	W6; W18; W35	715–718	Up to 45.7	SM_V7_W014	1	53,884	53,883
6	W7.2	W7	980	29	SM_V7_W016	1	28,424	28,423
7	W8.2	W8	538	60.6	SM_V7_W015	1	32,211	32,210
8	W10.2	W10	671	59.2	SM_V7_W021	1	39,689	39,688
9	W11.2_W002	W11; W14; W28	903	33.8	SM_V7_W002	712,429	755,874	43,445
	W11.2_ZW	W11; W14; W28	1,294	29.5	SM_V7_ZW	1,1804,837	11,863,496	58,659
10	W12.2	W12	475–499	Up to 46.1	SM_V7_W004	206,491	279,311	72,820
11	W13.2	W13; W17; W20; W33	524–646	Up to 23.8	SM_V7_W003	229,593	276,593	47,000
12	W16.2	W16; W21	317	204.2	SM_V7_W007	57,292	122,025	64,733
13	W22.2	W22	604	106.6	SM_V7_W008	1	64,369	64,368
14	W24.2	W24	636	3.6	SM_V7_W020	1	2,259	2,258
15	W25.2	W25	415–428	Up to 115.4	SM_V7_W012	1	49,306	49,305
16	W26.2_W003	W26	399–402	Up to 146.7	SM_V7_W003	1	161,189	161,188
	W26.2_W010	W26	400	152.6	SM_V7_W010	1	61,065	61,064
17	W27.2	W27; W29	403	101.3	SM_V7_W017	1	40,810	40,809
18	W31.2	W31; W34	260	205.5	SM_V7_W004	41,351	114,609	73,258
19	W36.2_W001	W36	333–335	173.4	SM_V7_W001	1	104,272	104,271
	W36.2_W005	W36	332	Up to 450.5	SM_V7_W005	1	149,646	149,645

Note.—Based on genome version V7, a number of 19 WEFs (WE V7) has been newly defined (first column, no. 1–19). Each WEF consists of one or more of those repeats that were previously found as individual WEs in version 5 of the genome (WE V5; Lepesant, Cosseau, et al. 2012). In some cases (no. 9, 16 and 19), representing WEF (W11.2, W26.2, and W36.2, respectively) WEs were found on two different scaffolds each associated with the W-chromosome and thus split into two subfamilies each. Furthermore, information of the length of WE monomers within WEFs is given as well as copy numbers (floating points indicate partial sequences), scaffolds with start and stop positions, and the total lengths of WEFs of the array. In some cases, the individual lengths of a WE monomer within a unit varied. For instance, a monomer of WEF 2.2 (no. 2) consists of W2 and W3 elements, which showed length variations between 709 and 711 bp. WEF 2.2 occurred “up to” 34.1 times, which means that the length variant 711 bp occurred 34 times whereas the 709 bp variant in a lower amount (supplementary fig. 2, Supplementary Material online).

**Table 2:** WEFs on Autosomes

WE	Length	Presence on Autosomes	Minimal–Maximal Length	Copy Numbers on Autosomes	WE on Autosomes
W2.2	709–711	All	37–705	68	Parts
W4.2	1,206	All	44–390	1,101	Parts
W5.2	1,104	All	26–742	3,454	Parts
W6.2	715–718	All	36–161	5,382	Parts
W7.2	980	2, 4, 6, 7	80–156	5	Parts
W11.2	903–1,294	All	30–514	18,610	Parts
W12.2	475–499	All	26–102	172	Parts
W13.2	524–646	1, 2, 4, 6	38–131	19	Parts
W16.2	317	All	26–314	203	Parts
W22.2	604	All	26–232	1,897	Parts
W24.2	636	All	28–450	2,302	Parts
W25.2	415–428	All	25–428	39,648	Parts and full-length (408)
W26.2	399–402	All	25–399	25,162	Parts and full-length (321)
W27.2	403	All	27–403	55,303	Parts and full-length (174)
W36.2	333–335	All	25–335	42,670	Parts and full-length (858)

Note.—Summary of the BLAST analyses of WEs against genome version V7 of *S. mansoni* in the NCBI database. Of the 19 newly defined WEFs, representatives of 15 WEF were found to be distributed among all 7 autosomes, whereas W7.2 and W13.2 were found on 4 autosomes. WEs, their original length (bp), their copy numbers on autosomes, and minimal and maximal lengths (bp) of the WE parts are listed. Next, the numbers of full-length and partial WEs on autosomes are given. Here, the numbers of full-length WEs for W25.2, W26.2, W27.2, and W36.2, respectively, are given in parentheses.

**Fig. 1:**
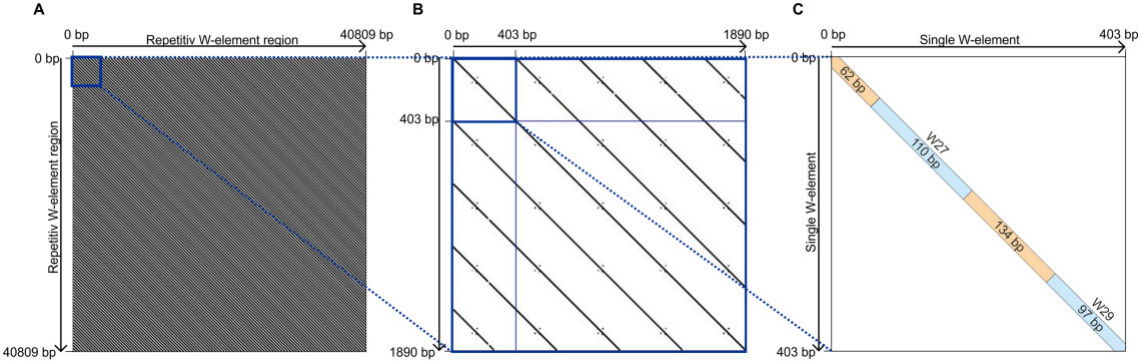
The new definition of WEF W27.2. Showcase for the new definition of WEFs in *S. mansoni*. (*A*) Result of the dotplot analysis for WE W29 in V7. The regular stripe pattern indicates a tandem repeat structure of this element without noticeable inversions or deletions. (*B*) Close-up of a small part of *A* (see blue square in *A*, upper left corner) showing a length of 403 bp for this WE unit. (*C*) A further close-up showing that W29 is closely associated to W27, which finally led to the new designation WEF W27.2 (table 1).

Next, we applied dotplot analyses to determine the structures of WEFs and the total lengths of WE monomers within the respective clusters (fig. 1). In relation to individual monomer repeat units, length variations from 82 (W32) to 1,132 bp (W4) occurred in V5 (supplementary fig. 2, Supplementary Material online). Furthermore, we found that some WEFs analyzed in V7 consist of sequences previously defined as separate repeat units in V5. For example, we found W27 and W29, originally described as monomer repeat units of 110 and 97 bp, respectively (Lepesant, Cosseau, et al. 2012), to be part of the same repeat unit (fig. 1). According to V7, the W27/W29 monomers are separated by spacer sequences of 134 and 62 bp, respectively, which together with the W27/W29 monomers form a new monomer of a total length of 403 bp (supplementary fig. 2*H*, Supplementary Material online). This 403-bp monomer represents a single repeat unit of the newly named W27.2 family. This designation relates to the former nomenclature used for these satellite-like repeat elements in schistosome research (Lepesant, Cosseau, et al. 2012). W27.2 consists of about 100 copies of highly similar monomer repeat units that define this WE family (fig. 1 and supplementary fig. 2, Supplementary Material online). We made similar findings for the majority of WEs/WEFs.

Therefore, with respect to the uninterrupted repetitive patterns of WEs within respective chromosome regions as well as small distances or overlaps of different WEs within a genome locus, we integrated some of the formerly described 36 WEFs (Lepesant, Cosseau, et al. 2012), which resulted in newly defined 19 WEFs (table 1 and supplementary fig. 2*A* and *B*, Supplementary Material online). These 19 WEFs vary in their copy numbers from 3 (W24.2) to 450 (W36.2) times at one or several chromosomal locations of the V7 genome assembly (table 1). These numbers may underestimate the actual copy numbers of the WEF in the genome because large satellite WEF-containing blocks are absent from the assembly.

### Some WEs Are Also Present on Autosomes

Former studies reported on the presence of WEs in male *S. mansoni* (Grevelding 1995; Quack et al. 1998). These findings suggested the presence of WE on Z and/or on autosomes. To find evidence for one or both possibilities, we took WE monomers of the newly defined WEFs and WE-transcript sequences detected in males for BLAST analyses against V7 in the NCBI database, which allowed assigning hits to individual chromosomes. For 4 out of 19 WEFs (W1.2, W7.2, W8.2, and W10.2), we found no autosomal localization. Represented by either full-length or partial WE sequences, 15 WEFs were distributed among all 7 autosomes (table 2 and supplementary fig. 3*A*, Supplementary Material online).

Next, we analyzed the distribution of single-size elements on autosomes and found heterogeneous patterns. In most cases, we detected size distributions patterns typical for individual WEF. The majority of autosomal W5.2 copies were comparably small, between 30 and 120 bp, with a copy-number bias for 56-bp long variants on chromosome 6 and another bias for 119-bp long variants on chromosome 1. Copy-number variants of W11.2 peaked in 241–244-bp long sequences with a clear bias of 412-bp-sized copies on chromosome 1, which contained also most of the dominating 81–88-bp variants of W25.2. W27.2 copies exhibited the largest distribution of different-sized variants across all chromosomes, whereas 150–165-bp long variants dominated the W36.2 family with a bias of 405 copies of 157 bp on chromosome 1. The majority of the longest size variants across all WEFs were found on chromosome 1. Among the few exceptions are W12.2 copies with the dominating size variant of 34 bp on chromosomes 5 (supplementary fig. 3*B*, Supplementary Material online).

For some of the parental WEFs of those partial, autosomal WEs, we identified sequence similarities to known mobile genetic elements (supplementary fig. 4, Supplementary Material online). W2.2 showed 65% sequence identity over a stretch of 607 bp to the LTR retrotransposon Saci-1 of *S. mansoni*, which is 5,980 bp in length (DeMarco et al. 2004). Open-reading frame (ORF) analysis indicated that this sequence stretch covered the complete protease-coding part of Saci-1. Within WEF W4.2 (1,206 bp) a short but significant sequence homology of 44 bp (89% identity) occurred to the LTR retrotransposon Boudicca (5,858 bp) (Copeland et al. 2003). A 120-bp fragment of W5.2 (1,104 bp) showed 96% identity to the non-LTR retrotransposon Perere-2 (4,544 bp) (DeMarco et al. 2005). This fragment covered part of the reverse transcriptase gene. In W11.2_ZW (1,294 bp), a partial sequence of 150 bp showed 84% identity to the non-LTR retrotransposon Perere-3 (DeMarco et al. 2005). Finally, in W16.2_ZW (1,294 bp) a partial sequence of 317 bp showed 81% identity to the DNA transposon Curupira-1 (4,878 bp) (Jacinto et al. 2011). An analysis of potential ORFs indicated partial sequence homologies of W2.2 to *gag* (Group antigen) and *pol* (a reverse transcriptase), and of W5.2 to endonuclease-reverse transcriptase and *pol* (data not shown). Genes like *gag*, *pol*, and endonuclease-reverse transcriptase are parts of mobile genetic elements such as retrotransposons or retroviruses. However, we did not find a complete ORF for a transposase or an endonuclease-reverse transcriptase in one of the investigated WEFs. The exception is W36.2_W001 (335 bp), which showed strong sequence similarity to the SMalpha family of SINE-like retrotransposons as reported in an independent study (Ferbeyre et al. 1998). Furthermore, a focused structural analysis on one element showed direct repeat sequences as part of a monomer of WEF W25.2. In this case, duplicate sequence stretches were identified flanking this WE at the presumptive target site. The duplicated sequences differed from the W25.2 sequence and could have originated from target site duplication (TSD) (supplementary fig. 5, Supplementary Material online; data not shown).

### WE Transcripts Occur in All Investigated Strains, Life Stages, Sexes, and the Gonads of *S. mansoni*

To get a first overview of WEF transcript occurrence in the different biological samples, we performed a sample-distance matrix analysis. By pairwise comparisons, this approach provided information about WEF transcript amounts based on the summation of all reads of transcribed WEs—independent of the composition of each WEF that contributed to the WE transcript pool of each sample. These samples included miracidia, cercariae, sporocysts, female and male schistosomula, paired and unpaired males and females, as well as testes and ovaries of paired and unpaired females and males, respectively, and also samples of other strains, depending on availability of sequencing data. We selected data sources with at least two biological replicates for the individual life stages, both sexes, and the gonads (supplementary table 1, Supplementary Material online) and normalized the data using DESeq2 prior to further analysis. When available, we also included replicates.

The result indicated remarkable differences in WE transcript amounts among the investigated samples. Furthermore, we observed clustering of samples with similar or dissimilar levels of the total amount of WE transcripts (supplementary figs. 6 and 7, Supplementary Material online). In the Liberian strain, miracidia (Mir_1, Mir_3, Mir_6) showed high deviations of WE transcripts compared with other stages but these levels differed from miracidia of the Puerto Rican strain (Mir). We detected similar deviations for cercariae of the Puerto Rican strain (Cer1-3) and the Liberian strain (CerM1–CerM3; CerF1–CerF3). Generally, schistosomula samples (SomF/M) and samples from males and testes (sM1–sM3; bM1–sM3; sT1–sT3, bT1–bT3) seemed to be homogeneous with respect to transcript amounts. Bigger differences occurred between unpaired females (sF1–sF3), which revealed an overall higher WE transcript level compared with paired females (bF1–bF3). Finally, we found differences also among biological replicates as exemplified by samples from females and ovaries (bF1–bF3; bO1–bO3; sO1–sO2), and even among replicates as observed for schistosomula (SomM/SomF samples). In summary, this first overview indicated a high degree of variability in overall WE transcript amounts between life stages, strains, and sexes, but also between biological replicates.

### WEF Expression Levels Differ among and within Strains, Life Stages, Sexes, Pairing Status, and Gonads of *S. mansoni*

**Fig. 2:**
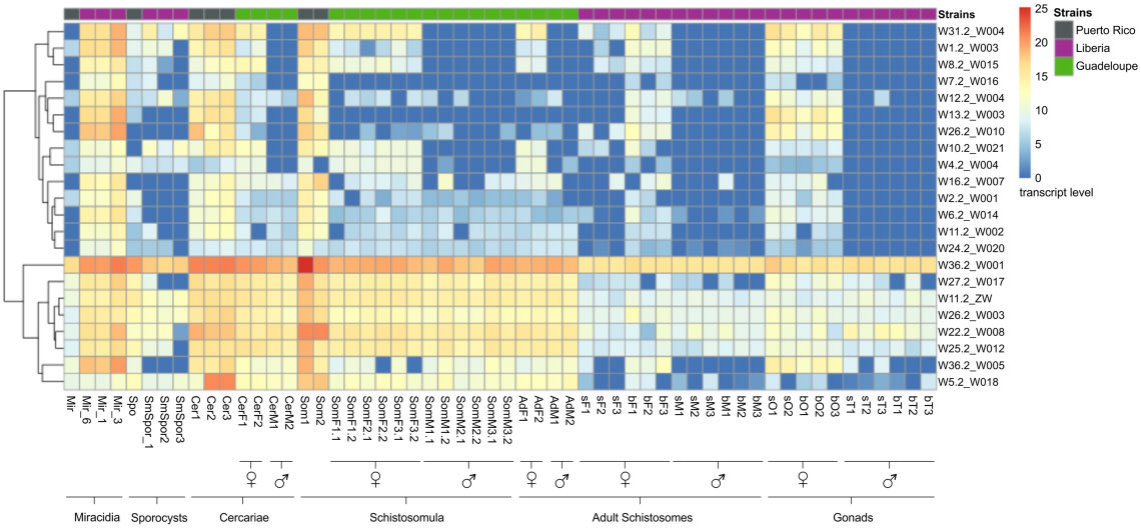
WE transcript profiles across different *S. mansoni* strains, life stages, sexes, and gonads. Heatmap generated by Deseq2 analysis showing the hierarchical clustering of transcript profiles of WEs of all 19 WEFs in the following life stages: Mir, miracidia; Cer, cercariae; (Sm)Spo(r), sporocysts; Cer(M/F), cercariae (male/female); Som(F/M), schistosomula (female/male); AdM, adult paired males; sF, unpaired (single-sex) females, bF, paired (bisex) females; sM, unpaired (single-sex) males; bM, paired (bisex) males; sO, ovaries of unpaired females; bO, ovaries of paired females; sT, testes of unpaired males; bT, testes of paired males. When available, biological and technical replicates were included. The first number behind a sample abbreviation indicates the number of the biological replicate. The second number indicates the technical replicate. For example, SomF1.1 indicates the first biological and first technical replicate of a female schistosomula, sample. SomF1.2 is the second technical replicate of this schistosomula sample. Samples without numbers had no replicate (supplementary table 1, Supplementary Material online). Furthermore, biological symbols indicate female and male samples. In cases without symbol, the sample origin was mixed sex. The horizontal line at the top of this figure shows a color code for the different schistosome strains, which were the basis for generating RNA-Seq data (supplementary table 1, Supplementary Material online). We used strains from Guadeloupe (beige), Liberia (purple), and Puerto Rico (gray). The dendrogram at the outer left side indicates relationships between appropriate WEs/WEFs as labeled at the outer right side. The color code indicates various levels of expression (from dark blue = no transcripts [0] to deep red = high transcript level [25] in the form of log2-transformed normalized counts of all data sets used).

Next, we performed differential expression analysis to investigate WEF expression profiles across strains, different life stages (from miracidia to adults), sex-, gonad-, and/or pairing-dependent expression. Again, we discovered remarkable differences (fig. 2 and supplementary figs. 8 and 9, Supplementary Material online). Strain-dependent expression occurred, among others, for W7.2, with transcripts in schistosomula of the Puerto Rican strain but not in schistosomula of the Guadeloupean strain. We found strain-dependent expression differences also between the Guadeloupean and Liberian strains. For example, W11.2 expression in males of the Guadeloupean strain was higher than the respective levels of males of the Liberian strain. Also with respect to schistosomula samples, we discovered differences between strains, here Guadeloupean and Puerto Rican. This applied also to miracidia samples from the Liberian and Puerto Rican strains, although here only one biological replicate was available for analysis.

Furthermore, differences in stage-dependent expression patterns occurred, within and among strains. Within the Liberian strain, for example, W13.2 appeared to be expressed in miracidia and paired females but not in sporocysts and males, respectively. In contrast, there was no W13.2 expression in females of the Guadeloupean strain. In the latter strain, no W13.2 transcripts occurred in schistosomula in contrast to schistosomula of the Puerto Rican strain, which expressed W13.2. We identified comparable differences for WEF expression comparing cercariae of both strains.

Furthermore, we found sex- and gonad-dependent expression patterns such as for W13.2 but also for W31.2. In these and other cases, expression levels were high in paired females and even higher in their ovaries. No W13.2 or W31.2 expression occurred in males and testes, a finding which applied to the majority of WE. In case of W31.2, an additional finding was the high expression level in miracidia and in ovaries, independent of the pairing status. Sex- and pairing-dependent expression occurred, among others, for W11.2 and W13.2 with transcripts in paired females but not in unpaired ones or in males. Also within biological replicates, independent of the strain, we identified in part differences in expression levels of some WEFs. For example, the expression level of W31.2 varied within three biological replicates of cercariae from the Puerto Rican strain. Also in the Liberian strain, WEF 31.2 expression varied among three biological replicates of miracidia, sporocysts, unpaired and paired females and their ovaries, respectively.

Finally, we observed the most persistent profile in these analyses for W36.2_W001, which appeared to be transcribed in all strains, life stages, sexes, and gonads. Overall expression levels of W36.2_W001 were higher in larval compared with adult stages, independent of the strain.

### Evidence for Noncoding RNAs in WEFs

Because WEF transcripts occurred, and we detected nearly no continuous ORFs indicating protein-coding information, we next investigated whether WEFs may contain ncRNAs. These RNAs encode no proteins, but as regulatory RNAs they can directly influence cellular processes (Mattick and Makunin 2006; Hombach and Kretz 2016). To search for ncRNAs, we analyzed the 19 WEFs using StructRNAFinder, which predicts and annotates RNA families in transcripts or genome sequences (Arias-Carrasco et al. 2018).

For sequence parts of WEF W1.2, W5.2, W7.2, W8.2, W11.2_ZW, and W12.2, StructRNAFinder predicted similarities to micro-RNA (miRNA) (supplementary table 2 and supplementary fig. 10, Supplementary Material online). According to the output of sequence similarities and structural predictions, some seemed more likely than others (supplementary fig. 11, Supplementary Material online). This class of short ncRNAs is processed from stem-loop regions of longer RNA transcripts and can influence post-transcriptional processes during gene expression (Bartel 2018). Transcripts of WEF possibly encoding miRNAs showed varying transcript amounts in life-cycle stages, strains, sex and tissue. For example, miRNA candidates mir-785 and mir-891 appeared to form stable, miRNA-like hairpin structures (supplementary fig. 11, Supplementary Material online). Transcripts of mir-785 sequence-containing W12.2 occurred in a strain-influenced (lower expression levels in the Guadeloupean strain compared with the other two strains; fig. 2 and supplementary fig. 8, Supplementary Material online) as well as stage-restricted and sex/gonad-influenced manner (high expression levels only in miracidia, paired females and ovaries compared with males and testes of the Liberian strain; supplementary fig. 9, Supplementary Material online). For mir-891 sequence-containing W8.2, we found sex-, gonad-, strain-, and stage-influenced patterns (fig. 2). Interestingly, in the Guadeloupean strain a clear sex-biased expression level of W8.2 occurred. We detected W8.2 transcripts in female cercariae, female schistosomula, and adult females, whereas no W8.2 transcripts occurred in the male-sample counterparts of this strain (supplementary fig. 8, Supplementary Material online). Among other possibilities, these observations suggest roles for W12.2 and W8.2 in sex-related processes.

For sequence parts of WEFs W2.2, W5.2, W11.2_ZW, W22.2, W24.2, and W26.2_W003, StructRNAFinder predicted similarities to small nucleolar RNAs (snoRNAs) (supplementary table 3, Supplementary Material online). Together with associated proteins, snoRNAs form ribonucleoprotein complexes directing the post-transcriptional modification of target RNAs (Lui and Lowe 2013). WEFs potentially encoding snoRNAs showed varying transcript amounts in different strains, life-cycle stages, sexes, and tissues. Among these is a candidate for SNORD59. It is potentially encoded by WEF W2.2, which contains the snoRNA C/D family-specific C-box (UGAUGA) and D-box (CUGA) motifs (Galardi et al. 2002) within the SNORD59 region of W2.2 (supplementary fig. 12, Supplementary Material online). The W2.2 expression level was higher in cercariae and schistosomula of the Puerto Rican strain compared with the Guadeloupean strain (supplementary fig. 8, Supplementary Material online). In the Liberian strain, W2.2 showed preferential expression in miracidia, paired females, and ovaries (supplementary fig. 9, Supplementary Material online). Expression levels of WEFs containing snoRNA candidates TB11Cs2H1 (W26.2_W003) and GlsR19 (W36.2_W001) in adults of the Guadeloupean strain exceeded those from adults of the Liberian strain (fig. 2). In the latter strain, W26.2_W003 and W36.2_W001 expression dominated in miracidia compared with other samples (supplementary fig. 9, Supplementary Material online). Within the sequence of W22.2, different snoRNA candidates were predicted, sR11, snoZ178, and SCARNA7 (supplementary table 3 and supplementary figs. 10 and 11, Supplementary Material online). W22.2 appeared expressed at higher levels in miracidia and schistosomula of the Puerto Rican strain compared with the Guadeloupean strain. In the Liberian strain, W22.2 expression is higher in miracidia than in other samples (supplementary fig. 9, Supplementary Material online).

In case of W5.2 and W11.2_ZW, StructRNAFinder predicted sequence parts for both miRNA and snoRNA, which partly overlapped (supplementary figs. 10 and 11, Supplementary Material online). W5.2 and W11.2_ZW expression levels appeared to be higher in the Puerto Rican strain compared with the Guadeloupean and Liberian strains (fig. 2 and supplementary fig. 8, Supplementary Material online). However, compared with W11.2_ZW showing constant expression among all samples of the Liberian strain, W5.2 expression was higher in miracidia and sporocysts than all other stages and tissues of this strain (supplementary fig. 9, Supplementary Material online).

At this stage of the analysis, however, it is unclear whether these predictions of miRNA and snoRNA correspond to biologically relevant ncRNAs. In addition, we found four WEFs that contain sequences reminiscent of the hammerhead ribozyme (HHR) class of ribozymes. These are catalytic RNAs and defined as ncRNA molecules that can catalyze chemical reactions (Lilley 2019; see next section).

### Some WEFs Contain Functional Hammerhead Ribozyme Sequences

To date, there are 14 natural ribozyme classes known that differ by their conserved secondary and tertiary structure. They are grouped according to the chemical reaction they catalyze. Of the 14 classes, 9 cleave their own phosphate backbone at a specific site by catalyzing a phosphoester transfer reaction and are therefore called self-cleaving ribozymes (Jimenez 2015, Weinberg et al. 2019). The first HHRs were discovered in plant virus-like satellite RNAs and viroids (Prody et al. 1986). Among the self-cleaving ribozymes, HHRs are abundant and can be found in all domains of life (de la Peña and Garcıa-Robles 2010; Perreault et al. 2011; Jimenez et al. 2011; Seehafer et al. 2011; Hammann et al. 2012) including representatives in schistosomes (Ferbeyre et al. 1998).

**Fig. 3:**
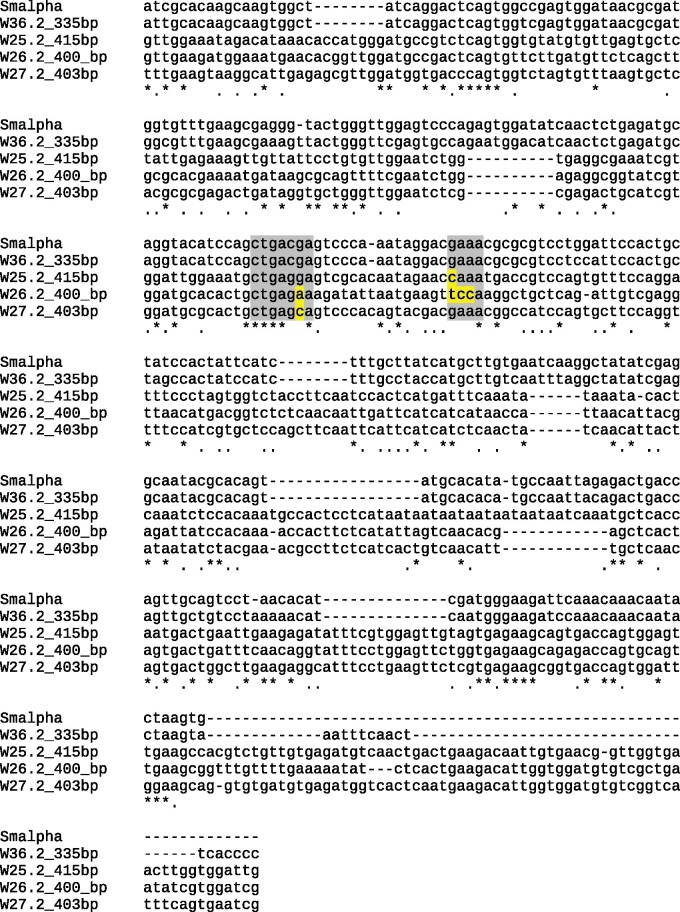
Alignment of WE example sequences from families W36.2, W25.2, W26.2, and W27.2 Compared with SMalpha. Clustal-based alignment of the WEF W36.2, W25.2, W26.2, and W27.2 compared with the SMalpha family of SINE-like retrotransposons (Ferbeyre et al. 1998), which contain the HHR sequence. The chosen examples show sequence similarity to the highly conserved core sequences CUGANGA and GAAA (gray background), but also deviations occurred (marked in yellow) (Ruffner et al. 1990; Perreault et al. 2011).

The HHR class is characterized by three helices (stems I–III) forming a junction that includes 12 highly conserved nucleotides. Together, these elements build the catalytic core of the HHR. If the transcription start and end lie within stem I, these ribozymes are referred to as type I HHRs. In *S. mansoni*, a type I HHR occurs as part of the SMalpha family of SINE-like retrotransposons (Ferbeyre et al. 1998), which is represented by the WEF W36.2_W001 in our study. To investigate the similarity between the HHR sequence of the W36.2_W001 and the HHRs found in other WEFs, we created a multiple sequence alignment using Infernal (Nawrocki and Eddy 2013). The results showed weak sequence similarity among W25.2, W26.2_W003, W27.2, and W36.2_W001 (fig. 3).

**Fig. 4:**
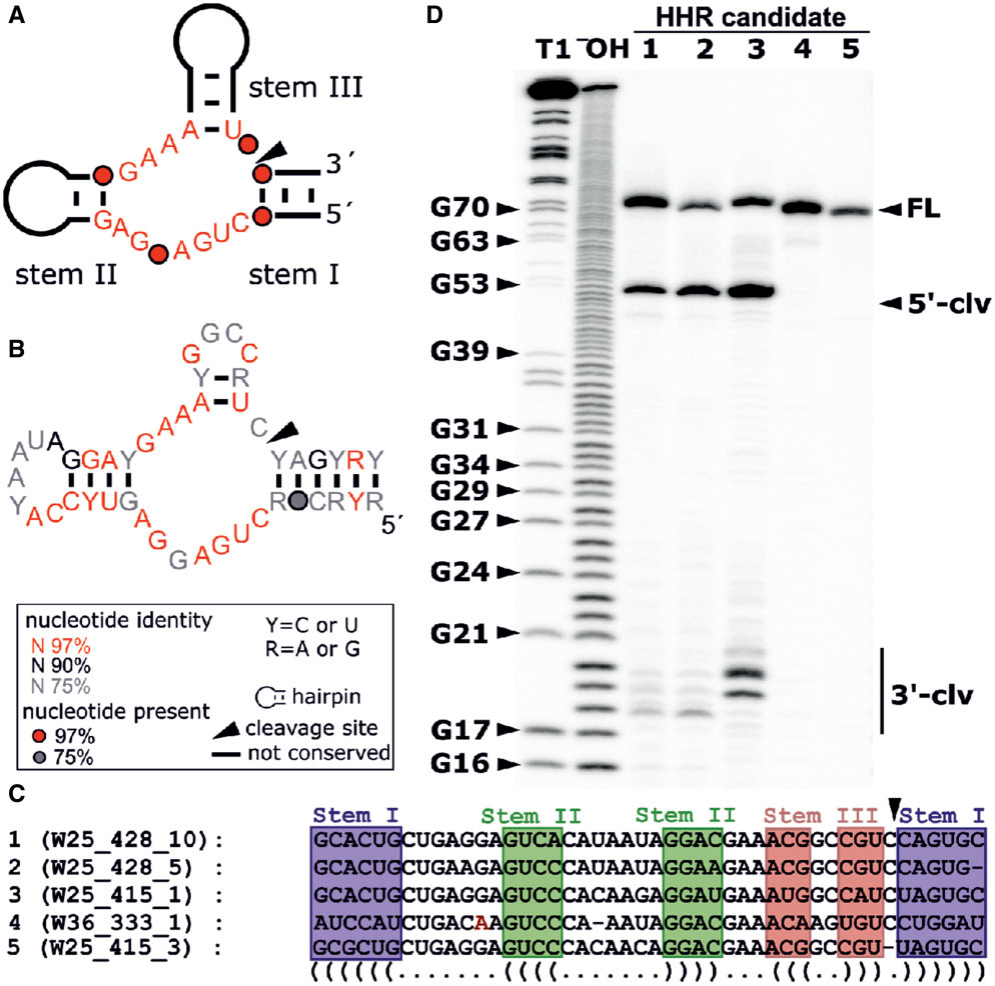
Active HHRs are part of autosomal WEs. HHR candidates as part of WEs were selected for functional analysis. (*A*) Consensus sequence and secondary structure of the HHR type I catalytic core. The stems are labelled I–III, and highly conserved nucleotides are shown in red. (*B*) Consensus sequence and secondary structure of HHR candidates found as part of WEs. Only those HHR candidates that contain all conserved sequence and structural features were included into this consensus (supplementary table 4 and supplementary fig. 13, Supplementary Material online). R2R was used to draw the model (Weinberg and Breaker 2011). (*C*) Alignment of HHR sequences from selected candidates tested for self-cleavage activity in vitro. The stems are highlighted, and mutations in highly conserved regions shown in red (i.e., Candidate 4-W36_333). At the cleavage site (arrow), candidate 5 W25_415_3 has a deletion and served as control. (*D*) Co-transcriptional cleavage analysis of selected HHR candidates. Full-length transcript (FL), 5′- (5′-clv), and 3′-cleavage (3′-clv) fragments are indicated by arrows. “T1” indicates partial digestion after G nucleotides by RNase T1 and “^–^OH” partial alkaline hydrolysis. Samples were separated by 20% PAGE.

Varying sequence similarity for HHRs occurred within these WEFs (fig. 4 and supplementary fig. 13, Supplementary Material online), which we also observed for W-chromosomal and autosomal copies of these WEFs showing incomplete or altered HHR consensus sequences with deletions, insertions, and point mutations. As shown before, mutations in the CUGANGA and GAAA consensus motifs can lead to inactivity of HHRs or diminished cleavage speeds (Ruffner et al. 1990).

Previous studies already confirmed the activity of the SMalpha-encoded type I HHR, represented by W36.2_W001 (Ferbeyre et al. 1998; Canny et al. 2004). Prior to experimental validation of activities of predicted HHRs of other WEFs, we analyzed the appropriate sequences for intact three-stem junctions and perfect conservation in the CUGANGA and GAAA sequence motifs to exclude presumably inactive HHRs (supplementary table 4, Supplementary Material online) (Ruffner et al. 1990). To investigate additional HHRs for their self-cleavage activity in vitro, we selected predicted candidates from different WEs (fig. 4). Because of the high similarity between all candidates (supplementary fig. 13, Supplementary Material online), we selected only five representatives. These candidates descend from WEF copies on autosomes and represent variants of W25.2 and W36.2 (fig. 4*C* and supplementary table 4, Supplementary Material online). Although the HHR core mainly comprised the three-stem junction with its conserved CUGANGA and GAAA sequences (fig. 4*A*), additional interactions outside the catalytic core increased ribozyme cleavage speed and structural stability (Khvorova et al. 2003; Uhlenbeck 2003; Martick and Scott 2006; Perreault et al. 2011). Therefore, we extended predicted HHR sequences by 11–13 nucleotides, which naturally occur at the 5′- and 3′-ends of the motifs.

Our analysis confirmed the activity of HHR candidates 1–3, which cleave into the expected 5'- and 3'-subfragments (fig. 4*D*). Due to the imprecise run-off transcription of the T7 RNA polymerase, we observed additional bands for the 3'-cleavage fragment (Chamberlin and Ring 1973). Candidate 4 contains a mutation in the catalytic core (CUGANAA), and candidate 5 has a deletion at the cleavage site. Both alterations likely rendered these candidates inactive based on detailed studies on the effect of mutations to the HHR core on cleavage (Ruffner et al. 1990). In summary, we confirm all predictions in this cotranscriptional cleavage assay.

### WEFs Vary among Different Schistosome Species

Previous studies discussed the existence of WEFs and mobile genetic elements such as retrotransposons in different schistosome species. In their original article on W1 elements in *S. mansoni*, now WEF W1.2, Webster et al. (1989) reported the absence of W1 from *S. matthei, S. haematobium, S. japonicum, S. douthitti*, and *Fasciola hepatica* based on Southern blot results in the pregenomic era. Furthermore, the SMalpha family (represented by W36.2), based on PCR data originally assumed by Ferbeyre et al. (1998) to be present in *S. haematobium* and *S. douthitti* but absent from *S. japonicum*, was later found in this species in high copy numbers and with the HHR core motif (Laha et al. 2000). Based on genome data for schistosomes (Howe et al. 2017), we searched for WEFs in *S. rodhaini*, which belongs to the same clade as *S. mansoni*, and in *S. haematobium* and *S. japonicum*, which represent different clades, respectively (Lawton et al. 2011).

BLASTn analysis showed the presence of the majority of WEFs in the other species. Compared with *S. mansoni*, however, lengths and structures of the elements varied. Most of the WEFs found in the other species revealed reduced sizes (supplementary table 5, Supplementary Material online). One example is W11.2_ZW, which in *S. mansoni* has a length of 1,294 bp, whereas in *S. japonicum* its size is 251 bp, in *S. haematobium* 375 bp, and in *S. rodhaini* 275 bp, respectively. Partial versions of five WEFs, W2.2_709, W4.2, and W7.2, occurred in all investigated species except *S. japonicum*. In *S. rodhaini*, a nearly complete version of WEF W31.2 exists, which is absent from *S. japonicum* and *S. haematobium*. Remarkably, full-length and partial versions of WEF W8.2 and W10.2 exclusively occurred in *S. mansoni*. Only for WEF W36.2, size differences of the repeat units were lower among the different species. For W36.2_W005 and W36_W001, we found slightly larger unit sizes in *S. rodhaini* (347 and 337 bp, respectively) compared with *S. mansoni* (332 and 335 bp, respectively) (supplementary table 5, Supplementary Material online).

**Fig. 5:**
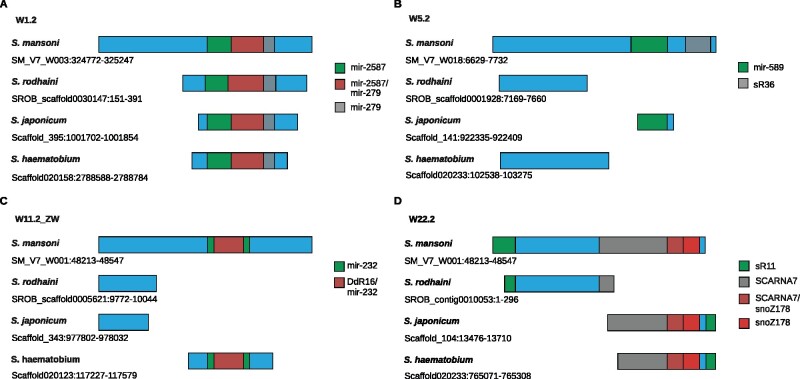
Structural comparison of selected WEFs in different schistosome species. Diagram of structural differences of selected WEF in *S. mansoni*, *S. rodhaini*, *S. japonicum*, and *S. haematobium* that potentially encode regulatory RNAs. (*A*) W1.2 (blue) harbors coding sequences for miRNAs mir-2587 (green) and mir-279 (gray). Compared with *S. mansoni*, best W1.2 hits upon BLASTn analysis showed shorter orthologs in *S. rodhaini*, *S. haematobium*, and *S. japonicum*. Although shorter, these orthologous sequences preserved full-length mir-279 and mir-2587. (*B*) W5.2 (blue) harbors coding sequences for mir-598 (green) and snoRNA sR36 (gray). We found none of these regulatory RNAs in orthologous sequences of *S. rodhaini* and *S. haematobium*. In *S. japonicum*, a partial mir-2587 sequence occurs, however, not flanked by W5.2 sequence areas. In this case, we cannot exclude a false-positive hit. (*C*) W11.2_ZW harbors overlapping coding sequences for mir-232 (green) and DdR16 (gray), which is part of the mir-232 sequence. This overlapping RNA-coding part appears to be completely preserved in *S. haematobium*, although the flanking W11.2_ZW sequence areas are shorter compared with *S. mansoni*. In *S. rodhaini* and *S. japonicum*, we detected smaller W11.2_ZW elements without RNA-coding parts. (*D*) W22.2 harbors sR11 (green) and overlapping coding sequences for SCARNA7 (gray) and snoZ178 (red), which is part of the SCARNA7 sequence (red/gray area). Only shorter W22.2 elements occur in the other schistosome species. In *S. rodhaini*, a small W22.2 variant exists containing fragments of the sR11 and SCARNA7 sequence. In *S. japonicum*, a big part of the overlapping RNA-coding region occurs with a small part of sR11 and a shortened SCARNA7 part, but a completely preserved snoZ178 part. We made a similar finding for *S. haematobium*, here the SCARNA7 (gray) part was slightly shorter compared with the *S. japonicum* counterpart. The sequences were obtained from data deposited on WormBase ParaSite (version 15, October 2020; https://parasite.wormbase.org), BioProject numbers: PRJEA36577 (*S. mansoni*), *S. rodhaini* (PRJEB526—Republic of Burundi), PRJNA520774 (HuSjv2, *S. japonicum)*, and PRJNA78265 (*S. haematobium*). Scaffold numbers are given.

To investigate whether the size variations structurally affected regions with hypothesized regulatory functions, we performed alignment analyses using the best BLASTn hits focusing on selected WEFs that contain regions potentially coding for regulatory RNAs, W1.2 (mir-279, mir-2587), W5.2 (mir-598, sR36), W11.2_ZW (mir-232; DdR16), and W22.2 (SCARNA7, sR11, snoZ178). This analysis indicated a mosaic pattern with respect to the presence and integrity of these parts of the WEF sequences that potentially code for regulatory RNAs. Whereas the part of W1.2 coding for the miRNAs mir-279 and mir-2587 was completely present in all investigated species, other *S. mansoni* miRNAs or snoRNAs were either absent or only partially preserved, or the W1.2 sequence was completely present in one or two further species (fig. 5).

**Fig. 6:**
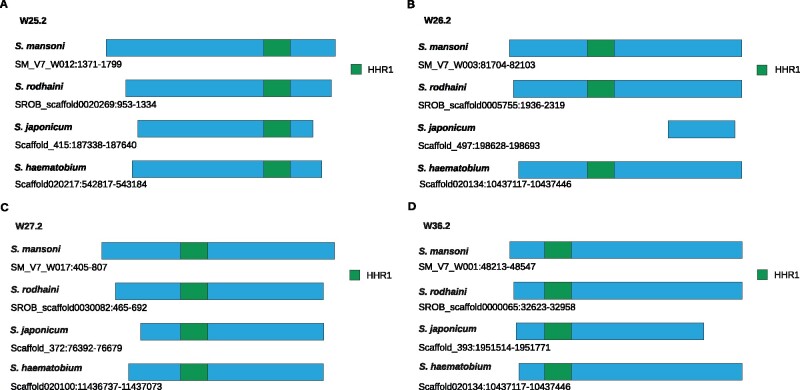
Structural comparison of HHR-containing WEFs in different schistosome species. Diagram of structural differences of WEF W25.2, W26.2, W27.2, W36.2 in *S. mansoni*, *S. rodhaini*, *S. japonicum*, and *S. haematobium* that potentially encode type I HHR1 (scaffold information is given). The ribozyme parts of these sequences are highlighted in green. Compared with *S. mansoni*, BLASTn analysis showed shorter orthologs for all four WEFs in *S. rodhaini*, *S. haematobium*, and *S. japonicum*. Except for W26.2 in *S. japonicum*, the ribozyme motif was highly conserved among the selected species. The sequences were obtained from data deposited on WormBase ParaSite (version 15, October 2020; https://parasite.wormbase.org), BioProject numbers: PRJEA36577 (*S. mansoni*), *S. rodhaini* (PRJEB526—Republic of Burundi), PRJNA520774 (HuSjv2, *S. japonicum)*, and PRJNA78265 (*S. haematobium*). Scaffold numbers are given.

For the HHR-containing WEF, species comparison showed a different picture. Looking again for best hits, we detected orthologs of three of four WEFs (W25.2, W27.2, and W36.2) in all examined schistosome species, with *S. mansoni* showing the longest size variants. In nearly all cases, the integrity of the HHR motifs was fully preserved. The exception was W26.2, of which only a small part without HHR motif appeared to be conserved in *S. japonicum* (fig. 6). When we searched for the HHR sequence of W26.2 in the *S. japonicum* genome by BLASTn, we found no hit (data not shown).

## Discussion

The days of “junk DNA” are over. When the senior authors of this article studied genetics at their respective universities, the common doctrine was that the nonprotein coding part of eukaryotic genomes consists of interspersed, “useless” sequences, often organized in repetitive elements such as satDNA. The latter might have accumulated during evolution, for example, as a consequence of gene duplication events to separate and individualize gene function (Britten and Kohne 1968; Comings 1972; Ohno 1999). This view has fundamentally changed (Biscotti, Canapa, et al. 2015), and our study is the first one addressing this issue with structural, functional, and evolutionary aspects for the genome of a multicellular parasite.

Using V7 of the *S. mansoni* genome we reanalyzed WEFs, which previous studies described as organized in 36 families of repetitive, nonprotein coding DNA elements occurring on the W-chromosome (Lepesant, Roquis, et al. 2012). From our results, we deduce 19 WEFs based on the fact that many of the original 36 WEFs are fused together. These 19 WEFs exhibit a surprising diversity of features. As expected for repetitive DNA, there is high sequence similarity but also variation among the repeat units of each single WEF. Besides few partial WE sequences of some WEFs on the WZ chromosomes, we found for 15 out of 19 WEF copies also on autosomes. Here, copy numbers considerably varied as well as the lengths of WE units of the respective WEFs. The autosomal occurrence possibly indicates the potential of element mobility. Indeed, in some WEF sequences we found residues of features typical for mobile genetic elements such as retrotransposons or retroviruses. Although we found no evidence of complete ORFs coding for a transposase, in an exemplary case, we detected direct repeats and TSD, which are often, but not always, found at integration sites of mobile genetic elements (Jensen et al. 1994; Han et al. 2014). Repetitive elements such as satDNA have been discussed to originate from transposable elements (TEs). Both share sequence similarity and organization patterns, which suggests a mutual relationship between satDNA and TEs. This probably influenced satDNA evolution and its roles on genome architecture and function (Meštrović et al. 2015; Satović et al. 2016). The mechanism of satDNA formation from TEs is unclear yet. However, structural elements such as palindromes, direct/inverted repeats, and the ability of stem-loop formation may be involved as well as illegitimate recombination events and deletions based on double-strand breaks and excision. These factors might drive rearrangements of TEs and the production of sequence parts as templates for further amplification to form tandemly organized satDNA clusters. This may apply to at least some of the WEs described here (W2.2, W4.2, W5.2, W11.2, and W16.2), which showed partial sequence homology to known mobile genetic elements. SatDNA monomers might be recognized by transposase-driven mechanisms acting on short DNA sequence motifs of these DNA sequences, which are recognized by enzymes related to transposases (Meštrović et al. 2015). As alternative to transposon-like cut and paste mechanisms, reintegration of repeat elements may happen via circular DNA intermediates generated from tandem repeats, as shown in *Arabidopsis* (Woodhouse et al. 2010). Whether this or similar mechanisms may apply to the WEF of *S. mansoni* has still to be elucidated.

Based on evidence for transcriptional activity and dynamic occurrence, recent years have unravelled novel roles for satDNA (Biscotti, Canapa, et al. 2015). Among these is the establishment of heterochromatic states at centromeres and telomeres, which is indispensable for preserving chromosome integrity and genome stability. In *S. mansoni*, Lepesant, Cosseau, et al. (2012) reported WEF-associated transcript occurrence and chromatin status of repeats to be stage specific. Open chromatin on the W-chromosome occurred in larval stages but not in the sexually dimorphic adult stage. Furthermore, the euchromatic character of histone modifications around the WE repeats on W decreased during the life cycle. When transcribed, repeat RNA appeared stage specifically in the larval stages miracidium and cercaria. These authors concluded that WEFs may play roles in structural changes of the chromatin and sex-chromosome emergence in ZW systems (Lepesant, Cosseau, et al. 2012). Furthermore, heterochromatization was discussed as major factor for the differentiation of sex chromosomes in the schistosomatids and also for schistosome speciation (Lawton et al. 2011).

Here, we provide conclusive evidence for WEF expression throughout schistosome development, from the miracidium to the adult stages. Copy numbers considerably varied among the WEFs and depended on the life-cycle stage, sex, pairing, and strain. In a Liberian strain of *S. mansoni*, genome instability of the W1 element was hypothesized from the finding of gain or loss of W1 elements in the progeny of crosses, or even during larval development within the snail host (Grevelding 1999). This pointed to recombination processes during meiosis and/or mitosis. Our new results suggest that a mobile character of WEF may account for this genome instability, which allows WEs to recombine within a single generation. This could also explain the observed strain differences. Accordingly, some of the results shown (fig. 2 and supplementary figs. 6–9, Supplementary Material online), may represent snapshots of the WEF setup within the samples at the time they were available for analysis. Therefore, WE occurrence and copy numbers may be a matter of change over time and generations, a view that is supported by the observed variations among biological and technical replicates. As practical consequence, sex determination of clonal cercarial populations based on PCR using WE-specific primers, as previously performed with varying success (Gasser et al. 1991; Boissier et al. 2001; Chevalier et al. 2016), may eventually lead to inconsistent results.

Our approach to assign putative roles to WEFs led to the StructRNAFinder-based prediction of different classes of regulatory RNAs as parts of WE sequences. Due to their roles in gene regulation, miRNAs have attracted attention (Bartel 2018). In mammals, X-chromosomal miRNA expanded by gene duplication after the emergence of sex chromosomes, and hundreds of different miRNAs have been identified shaping mRNA expression (Meunier et al. 2013). Many are conserved, which may also applies to *S. mansoni* (http://www.mirbase.org/; last accessed September 2021; Kozomara et al. 2019). As example, mir-181, in our study predicted as putative part of W8.2, may represent a miRNA family for which multiple roles in immune cell development, hematopoiesis, cell death pathways, cancer, and drug resistance have been reported in humans (Weng et al. 2015; Braicu et al. 2019). Species comparison indicated W8.2 in *S. mansoni* but not in the closely related species *S. rodhaini* and *S. japonicum* or *S. haematobium*. In *Drosophila*, mir-279, potential part of W1.2, influence neuron formation of the olfactory sensory system (Hartl et al. 2011) and eye patterning interfering with EGFR pathways (Duan et al. 2018). Roles for miRNAs have also been reported for parasites and host–parasite interaction (Zhao and Guo 2019; Britton et al. 2020). Plant-parasitic nematodes induce feeding site formation in host cells, which differentially express miRNAs upon infection (Jaubert-Possamai et al. 2019). In schistosomes, the presence and conservation of genes involved in miRNA pathways and their role in *B*. *glabrata/S*. *mansoni* interaction has been discussed (Queiroz et al. 2017; Cardoso et al. 2020). Furthermore, miRNAs of *S. mansoni* and *S. japonicum* may participate in male–female interaction, sexual development, and pathological processes in the final host (Zhu et al. 2014; Cai et al. 2016; Yu et al. 2019). In *S. haematobium*, a genome-wide analysis of small ncRNAs identified mir-785 expression in both sexes, which corresponds to our findings. Furthermore, homology-based prediction indicated a voltage-dependent anion-selective channel protein (MS3_05034) as potential target (Stroehlein et al. 2018). Transcripts of the MS3_05034 ortholog of *S. mansoni*, Smp_091240, occur in males, females, and their gonads (Lu et al. 2016). *Schistosomamansoni* mir-785 is also expressed in adults although with a sex bias (females > males) including the gonads (ovary ≫ testes), and influenced by pairing (paired females ≫ unpaired females).

Also, snoRNAs are widely distributed among eukaryotes and participate in the modification and processing of ribosomal and small-nuclear RNAs, splicing, rRNA acetylation, mRNA abundance and translational efficiency (Lui and Lowe 2013; Bratkovič et al. 2020). SNORD59, a putative snoRNA in W2.2, is a member of the C/D family directing site-specific 2'-O-methylation of substrate RNA such as 18S rRNA (Galardi et al. 2002). The conserved C/D family-specific C-box (UGAUGA) and D-box (CUGA) motifs are present in W2.2-encoded SNORD59. Perfect matches occur in the 709 bp variants of W2.2, whereas in the 711 bp variants, a G/C mutation has been found at position 3 of the D-box motif (supplementary fig. 12, Supplementary Material online). The 2′-O-methylation of the ribose moiety is important for the maturation of almost all classes of RNAs and involves different snoRNP (ribonucleoprotein) complexes (Kiss-László et al. 1996; Ojha et al. 2020). They contain nucleolar proteins (Nop), of which orthologs exist in *S. mansoni* such as Nop 56 (Smp_053470 and Smp_048660; https://parasite.wormbase.org). Smp_053470 and Smp_048660 are most abundantly expressed in sporocysts and ovaries of paired females (Lu et al. 2016, 2018; http://schisto.xyz/, V7), which coincides with SNORD59 transcripts in these stages/tissues (supplementary table 3, Supplementary Material online). SnoRNAs can be further processed into smaller RNAs with different functionality including miRNAs (Scott and Ono 2011). In mammals, protein-coding genes exist that express both snoRNAs and miRNAs in single introns. The existence of eukaryotic and archean members suggested that snoRNAs—in evolutionary terms—are more ancient compared with miRNA (Dennis and Omer 2005). However, there is also evidence for recently evolved snoRNA and miRNAs (Niwa and Slack 2007). This suggests that both RNA classes evolve dynamically and at fast rates, which may also apply to the predicted *S. mansoni* snoRNA and miRNAs. Furthermore, many of the most recently evolved snoRNA and miRNAs may be derived from TEs. Indeed, previous studies showed TSDs at snoRT (human snoRNA-like retrogene) integration sites, which supports their mobile character (Weber 2006; Lui and Lowe 2013). Our indicative findings of TSDs and intra-WE inverted repeats flanking miRNA and snoRNA subunits of WEFs as well as their mobile character correspond to these concepts. To prove whether the predictions of snoRNAs and miRNAs as parts of appropriate WEFs have functional relevance will be subject of future studies. In this study, we focused on functional evidence of type I HHRs, which are parts of some WEF. HHRs are widely distributed in the animal and plant kingdoms, and they can be associated with repeated DNA (Perreault et al. 2011; Cervera and de La Peña 2014; de La Peña et al. 2017; Lünse et al. 2017). Recent studies on the biology of genomic HHRs discuss their potential roles in the propagation of retrotransposons, which are major components of eukaryotic genomes including schistosomes (Venancio et al. 2010), and which contribute to genome evolution shaping developmental processes and eukaryotic complexity (Mita and Boeke 2016). Remarkably, our species analysis showed a high conservation of the ribozyme-like sequences in W25.2, W26.2_W003, W27.2, and W36.2_W001 among the examined species with the exception of the absence of the type I HHR in W26.2 in *S. japonicum*. To test whether WEF-encoded HHRs are catalytically active, we selected predicted ribozyme sequences of autosomal WEs. Alignments showed that some WEF-encoded HHR sequences harbor the canonical core sequences CUGANGA and GAAA. Other HHRs, such as candidates 4 (W36_333_1) and 5 (W25_415_3), have mutations or deletions in highly conserved nucleotides that likely lead to a reduction or loss of the cleavage activity (Ruffner et al. 1990). Indeed, all HHR candidates conform to previously described minimal HHRs with an extremely short stem III (Forster and Symons 1987; Lünse et al. 2017). Although only weak similarity to SMalpha HHR existed, it may be tempting to speculate about their functional role as part of SINE-like elements similar to the ones already described (Ferbeyre et al. 1998). Furthermore, W25.2 showed higher expression in the Puerto Rican and Guadeloupean strains compared with the Liberian strain. Thus, it may contribute to strain-specific differences at the post-transcriptional level. Within the Liberian strain, W25.2 appeared to be expressed at a slightly higher level in the ovary of females compared with male testes or the adults. This suggests a gonad-associated function in females, which awaits confirmation in subsequent studies. These are needed to substantiate the expounded hypotheses and to assure that *S. mansoni* W-Eelements are not just selfish DNA (Biscotti, Olmo, et al. 2015, Thakur et al. 2021).

Creating variability and genome plasticity are hallmarks of parasites with different molecular principles invented during evolution (Lanzer et al. 1995). In schistosomes, repetitive WEs may represent one of these principles. Using the intermediate snail-host stage for asexual recombination (Grevelding 1999; Bayne and Grevelding 2003) and the final host stage for sexual recombination, schistosomes have exceptional preconditions for rapid evolution driven by adaptation to new environments. Prerequisite for asexual recombination of WEFs from the heterochromatin area of the W-chromosome is a biphasic chromatin stage, in which both euchromatic and heterochromatic states can be adopted. Indeed, this has been confirmed for *S. mansoni* by showing that euchromatic histone modifications around WEs dominate in the intermediate snail host but decrease afterwards (Lepesant, Cosseau, et al. 2012). In *P. falciparum*, bistable chromatin has been discussed as a mechanism regulating variant gene expression patterns within clonal populations (Llorà-Batlle et al. 2019). In nematodes, mobile genetic elements contribute to genome plasticity in the absence of sexual reproduction (Castagnone-Sereno and Danchin 2014). This fits to the general view of the impact of mobile genetic elements on genome evolution and adaptation, which is important for organisms frequently facing new environments such as parasites (Schrader and Schmitz 2019), particularly those with complex life cycles. The evolution of repetitive DNA was also associated with reproductive isolation, founding new species, genome integrity, and karyotype evolution (Coghlan 2005; Biscotti, Canapa, et al. 2015; Lower et al. 2018). Especially with respect to heterochromatic regions, remarkable diversity of karyotype patterns exist for different schistosome species leading to the definition of six clades correlating with the different geographical distribution as well as with the hypothesized Asian origin of schistosomes (Hirai et al. 2000; Lawton et al. 2011). Our comparison of WEFs in selected schistosome species of different clades provide a first hint for their potential contribution to karyotype variability, and thus speciation. This may include a varying repertoire of WEF-encoded regulatory RNAs, which differs between these species.

Repetitive DNA discloses high sequence and copy number variability among and within species but also in closely related organisms, which points to rapid evolution (Lower et al. 2018). This may involve coevolution with regulatory RNAs as it was hypothesized for satellite repeats and long noncoding RNAs (Lee et al. 2019) as well as the dual relationship between Alu repeats (short interspersed nuclear elements of the human genome) and miRNAs. Duplication events involving Alu elements have favored the expansion of miRNA clusters and their expression (Lehnert et al. 2009).

From the data obtained in our study and against the background of recent literature, it is tempting to speculate that more of the WE “junk-DNA” than expected might be functional and relevant. WEs of all investigated WEFs exhibit a capricious incidence, and they are transcribed in a stage-, sex-, pairing-, gonad, and strain-specific or preferential pattern. From exemplary findings of features typical for the activity of mobile genetic elements, we hypothesize that WEs may have a mobile character. Together with previous findings of intraclonal recombination events of WEs (Grevelding 1999; Bayne and Grevelding 2003), their presumptive role in sex chromosome emergence (Lepesant, Cosseau, et al. 2012), their putative capacity to express regulatory RNAs, we propose that WEs might influence the biology of *S. mansoni*. Furthermore, based on the variable occurrence of WEFs in different schistosome strains, isolates, and even species, we hypothesize that the WEs represent one of the sources of heritable variability in the evolution of the family Schistosomatidae.

## Materials and Methods

### Ethics Approval

Experiments with hamsters to obtain *S. mansoni* material as basis for RNA-seq studies leading to the bioinformatics data analysis were done in accordance with the European Convention for the Protection of Vertebrate Animals Used for Experimental and Other Scientific Purposes (ETS no. 123; revised Appendix A) and had been approved by the Regional Council (Regierungspraesidium) Giessen (V54-19 c 20/15 c GI 18/10).

### Mapping and Characterizing WEF (V5) against Genome Version V7

WE repeat sequences, originally described as being organized in 36 families (Lepesant, Roquis, et al. 2012), were identified in the current genome version V7 of *S. mansoni* by local alignment searches via BLASTn (BLAST+, v. 2.6.0) (Camacho et al. 2009) using a coverage cut-off of 66% and an identity cut-off of 80%. In addition, all WEFs were also detected with Gepard (GEnome PAir—Rapid Dotter, v. 1.40) through the representation of the repetitive patterns in dotplot graphics (Krumsiek et al. 2007). Dotplot analysis (Gibbs and McIntyre 1970) was performed and visualized as two-dimensional matrices with sequences being compared along vertical and horizontal axes. In case of identity, individual cells within the matrix are shaded black, thus matching sequence segments appear as diagonal lines across the matrix. We also used Gepard to provide a graphical overview of existing patterns that are typical for mobile genetic elements such as transposons.

### Mapping and Counting of RNA-Seq Reads

First, we used the tool Trim Galore (https://www.bioinformatics.babraham.ac.uk/index.html; last accessed September 2021), a wrapper script to automate quality and adapter trimming as well as quality control, to remove the adapters required for Illumina sequencing from the RNA-Seq reads. In order to detect WE transcripts, RNA-Seq reads of different samples were aligned to a Multifasta file with all WE sequences using Bowtie2 (version 2.3.4.3; Reporting Option: all alignments) (Langmead et al. 2009; Langmead and Salzberg 2012). Using bedtools intersect (Quinlan and Hall 2010; version 2.27.1+galaxy1), RNA-Seq reads were screened for overlaps with WEF sequences to produce the raw read counts. To this end, we used the mapped reads of the Bowtie2 (bam file) analysis and a bed file containing length information of all WEF (table 1).

Additionally, we included RNA-Seq reads of all protein-coding genes in *S. mansoni* to increase the library sizes for between-sample normalization. Such read counts were obtained as described before (Lu et al. 2018). Briefly, the quality of raw RNA-seq reads was assessed using the FastQC tool (http://www.bioinformatics.babraham.ac.uk/projects/fastqc/; last accessed September 2021), and reads were aligned to *S. mansoni* V7 genome (WormBase Parasite WBPS14) using STAR 2.7.2a (Dobin et al. 2013) with the option *–alignIntronMin 10.* Counts per gene were summarized with FeatureCounts v1.4.5-p1 (Liao et al. 2014) based on the exon feature, using the annotation from WormBase Parasite WBPS14 (https://parasite.wormbase.org/).

### Open-Reading Frame Analysis

We used the NCBI program “ORFfinder” (https://www.ncbi.nlm.nih.gov/orffinder/; last accessed September 2021) to detect translatable sequence areas. For the analysis, we selected a minimum length of 30 nucleotides and only the start codon “ATG.” The amino acid sequences were examined by BLASTp (BLAST+, v.2.6.0; Camacho et al. 2009) against UniProtKB/Swiss-Prot.

### Differential Expression Analysis and Normalization

To determine quantitative changes in WE transcript levels between the RNA-Seq data sets, we performed differential expression analysis using raw reads in DESeq2 (Love et al. 2014). Throughout the manuscript, expression is defined as occurrence or levels of transcripts. For normalization of the data sets, we combined WE-specific read counts and read counts of protein-coding genes (Smp identifiers). For generation of the heatmap of the count matrix and the sample-distance matrix, we used R (https://www.r-project.org, v3.6.3; last accessed September 2021) package DESeq2 (v1.24.0) and pheatmap (v1.0.12), applying log2 transformation of normalized counts of WEs using the *normTransform* and *rlogTransformation* function, respectively.

### Identification of WEs on Autosomes

We used BLAST+ (v.2.6.0; Camacho et al. 2009) to identify highly similar sequences of WEs on autosomes, applying the megablast task and a 0.001 *E*-value cut-off. We only extracted sequences if the alignment covered more than 80% of the query sequence, and if the overall alignment percentage (OAP; percent identity of the alignment multiplied by the coverage divided by 100) was higher than 60. Exceptions of this rule were made in case fragmented autosomal WEs were small; fragment sizes below 25 bp were not considered. Using the sequence alignment tool MAFFT (v7.471; https://mafft.cbrc.jp/alignment/server/; last accessed September 2021), we calculated sequence homologies (Kuraku et al. 2013; Katoh et al. 2019). As input, we used the Fasta sequences and chose “Adjust direction according to the first sequence.” Clustal-formatted alignments were produced as results. To find patterns of WE mobile activity, such as potential transposition events, we used MITE Tracker, an open source program that provides positional information on inverted-repeat sequences and TSDs (Crescente et al. 2018).

### Functional Prediction

For the prediction of sequences coding functional RNA, we used StructRNAfinder, an integrative tool allowing the identification, functional annotation, and taxonomic allocation of sequences to RNA families by secondary structure inference (https://structrnafinder.integrativebioinformatics.me/run.html; last accessed September 2021) (Arias-Carrasco et al. 2018). StructRNAfinder displays sequence consensus alignments for RNA families, according to Rfam database (RNA families, data base version 14.2, Kalvari et al. 2018; https://rfam.xfam.org; https://structrnafinder.integrativebioinformatics.me/run.html), but also provides a taxonomic overview for each assigned functional RNA. As input, we generated FASTA files with complete RNAs from the Bowtie2 output by using the tool “bedtools MergeBED” (Galaxy Version 2.27.1) (Quinlan and Hall 2010) to merge overlapping and adjacent regions. We chose the option cmsearch and an *E*-value of 0.01. In the output options, we selected “report only best hit per sequence.”

### Determination of Ribozyme Activity during In Vitro Transcription

We produced transcription templates containing the T7 promoter sequence by extension of partly complementary oligonucleotides (supplementary table 6, Supplementary Material online) using the Phusion DNA Polymerase (ThermoScientific). We purified templates by phenol/chloroform extraction and ethanol precipitation. For in vitro transcription, 1 µg purified template, 1× transcription buffer, 3% DMSO, 4 mM NTPs, 0.013 U thermostable inorganic pyrophosphatase (NEB), and 25 ng of T7 RNA polymerase (laboratory preparation) were combined in a 30-µl reaction and incubated at 37°C for 2 h. The 1× transcription buffer contained 80 mM HEPES-KOH (pH 7.5), 24 mM MgCl_2_, 2 mM Spermidine, and 40 mM DTT. Labeling of HHR candidates during transcription occurred by the addition of [α^32^P]-CTP. We stopped reactions by adding RNA loading dye composed of 2.5 mM Tris–HCl (pH 7.6), 20% formamide, 0.06% bromophenol, and 0.06% xylene cyanol. A cleavage-deficient, elongated variant of W25_415_3 (154 nt) was gel purified after in vitro transcription, dephosphorylated using the antarctic phosphatase (NEB**)** and labeled at its 5'-end using the T4 polynucleotidyl kinase (NEB) and [γ^32^P]-ATP. Following another gel purification, we used the 5'-labeled RNA to create a size standard by alkaline hydrolysis and RNase T1 digestion. For experimental validation and product visualization, we separated the size standard and internally labeled ribozyme cleavage products by denaturing 20% polyacrylamide gel electrophoresis (PAGE) and detected the bands with an Amersham Typhoon Imager (GE Healthcare).

## Supplementary Material

Supplementary data are available at *Genome Biology and Evolution* online.

## Supplementary Material

evab204_Supplementary_Data

## Data Availability

All sequencing data underlying this article and used for analysis in this study are available at ENA (http://www.ebi.ac.uk/ena; last accessed September 2021). These data originated from published and yet unpublished studies covering adult and larval schistosomes (Grevelding 1995; Protasio et al. 2012; Wang et al. 2013; Lu et al. 2016; Picard et al. 2016; Lu et al. 2017; https://www.ebi.ac.uk/ena). Sample types, strains, replicates, accession numbers, and references are listed in supplementary table 1, Supplementary Material online. Further sequence information, as indicated in the text, was obtained from WormBase ParaSite (version 15, October 2020; https://parasite.wormbase.org; last accessed September 2021), BioProject numbers: PRJEA36577 (*Schistosoma mansoni*), *S. rodhaini* (PRJEB526—Republic of Burundi), PRJNA520774 (HuSjv2, *S. japonicum)*, and PRJNA78265 (*S. haematobium*). Finally, all WEF sequences can be downloaded from zenodo (https://zenodo.org/) using the link: https://doi.org/10.5281/zenodo.5482269.
