# Supplementary material for: Satellite-Like W-Elements: Repetitive, Transcribed, and Putative Mobile Genetic Factors with Potential Roles for Biology and Evolution of Schistosoma mansoni
Source: Genome Biol Evol. 2021 Sep 1;13(10):evab204. doi: 10.1093/gbe/evab204 (PMC8490949; doi:10.1093/gbe/evab204)
Supplement: evab204_Supplementary_Data [file evab204_supplementary_data.zip › Stitz et al_Supplementary Material.pdf]

Supplementary material

I. Supplementary Figures

1. Page 2, Supplementary Figure 1: BLASTn results of W6 and W23 repeat elements listing the 10 most significant hits within genome version V7 of *S. mansoni*.
2. Pages 3-12, Supplementary Figure 2: New definition of WEF families (A) and their sequences (B).
3. Pages 13-16, Supplementary Figure 3: WE sequences found on autosomes in males.
4. Pages 16-19, Supplementary Figure 4: Similarities between individual WEF and mobile genetic elements.
5. Page 20: Supplementary Figure 5: Autosomal occurrence of W25.2 on chromosome 4.
6. Page 21-22: Supplementary Figure 6: Sample-distance matrix analysis shows WE transcript occurrence across all analysed samples.
7. Page 22: Supplementary Figure 7: Log_2 values of WEF (see separate Excel file)
8. Page 23: Supplementary Figure 8: Comparison of WEF transcript profiles between the Puerto Rican and the Guadeloupe strains of *S. mansoni*.
9. Page 24: Supplementary Figure 9: WEF transcript profiles among all samples of the Liberian strain of *S. mansoni*.
10. Pages 25-26: Supplementary Figure 10: miRNA and snoRNA motifs within exemplary WEFs.
11. Page 26: Supplementary Figure 11: Rfam annotation and putative secondary structures of the predicted miRNAs and snoRNAs (see separate Excel file)
12. Page 27: Supplementary Figure 12: SNORD 59 is a predicted sequence part of WEF W2.2.
13. Page 28: Supplementary Figure 13: Multiple sequence alignment of SMalpha-HHR and HHR-like sequences found within WEFs.

II. Supplementary Tables

1. Pages 29-30: Supplementary Table 1: Samples and sources of *S. mansoni* used in this study.
2. Page 31, Supplementary Table 2: miRNAs predicted to be parts of WEFs.
3. Page 32, Supplementary Table 3: snoRNAs predicted to be parts of WEFs.
4. Page 33-34, Supplementary Table 4: Prediction of self-cleavage activity of different HHsR candidates found in W-element families (WEFs) in *S. mansoni*
5. Page 34, Supplementary Table 5: WEF occurrence in *S. mansoni, S. japonicum, S. haematobium, and S. rodhaini* (see separate Excel file)
6. Pages 35-36, Supplementary Table 6: Oligonucleotides used in this study.

I. Supplementary Figure 1.

BLASTn results of W6 and W23 repeat elements listing the 10 most significant hits within genome version V7 of *S. mansoni*

A

qseqid	sseqid	pident	length	mismatch	gapopen	qstart	qend	sstart	send	E-value	bitscore
W6	SM_V7_W014	100	310	0	0	1	310	3038	3347	2.42E-158	560
W6	SM_V7_W014	100	310	0	0	1	310	3780	4089	2.42E-158	560
W6	SM_V7_W014	100	310	0	0	1	310	4497	4806	2.42E-158	560
W6	SM_V7_W014	100	310	0	0	1	310	20172	20481	2.42E-158	560
W6	SM_V7_W014	100	310	0	0	1	310	21248	21557	2.42E-158	560
W6	SM_V7_W014	100	310	0	0	1	310	21953	22262	2.42E-158	560
W6	SM_V7_W014	100	310	0	0	1	310	22661	22970	2.42E-158	560
W6	SM_V7_W014	100	310	0	0	1	310	23373	23682	2.42E-158	560
W6	SM_V7_W014	100	310	0	0	1	310	24093	24402	2.42E-158	560
W6	SM_V7_W014	100	310	0	0	1	310	24809	25118	2.42E-158	560

B

qseqid	sseqid	pident	length	mismatch	gapopen	qstart	qend	sstart	send	E-value	bitscore
W23	SM_V7_W003	100	125	0	0	1	125	321741	321617	2.26E-58	226
W23	SM_V7_W003	79.3	111	23	0	1	111	466977	466867	2.75E-19	96.9
W23	SM_V7_W003	76	125	30	0	1	125	324120	323996	1.17E-17	91.5
W23	SM_V7_W003	76	125	30	0	1	125	468401	468277	1.17E-17	91.5
W23	SM_V7_W003	76	125	30	0	1	125	476942	476818	1.17E-17	91.5
W23	SM_V7_W003	76	125	30	0	1	125	478366	478242	1.17E-17	91.5
W23	SM_V7_W003	76	125	30	0	1	125	478841	478717	1.17E-17	91.5
W23	SM_V7_W003	76	125	30	0	1	125	479316	479192	1.17E-17	91.5
W23	SM_V7_W003	76	125	30	0	1	125	480740	480616	1.17E-17	91.5
W23	SM_V7_W003	76	125	30	0	1	125	485959	485835	1.17E-17	91.5

Suppl. Fig. 1: **A**, Representative BLASTn result of WE W6 listing the 10 most significant hits (out of 81) within V7. **B**, representative BLASTn result of WE W23 listing the 10 most significant hits (out of 162) within V7.Abbreviations: qseqid = sequence-ID (WE W6); ssedid = chromosomal area; pident = percent identity; length = length of alignment; mismatch = counts of non-matching bases; gapopen = counts of gaps; qstart = start of alignment within W6; qend = end of alignment within W6; sstart = start of alignment within V7; send = end of alignment within V7; E-value = number of expected hits of similar quality that could be found by chance; bitscore = required size of a sequence database in which the current match could be found just by chance. The used nucleotide collection database contained 60,954,042 sequences.

Supplementary Figure 2.

New definition of WEF families (<u>A</u>) and their sequences (<u>B</u>)

<u>A</u>

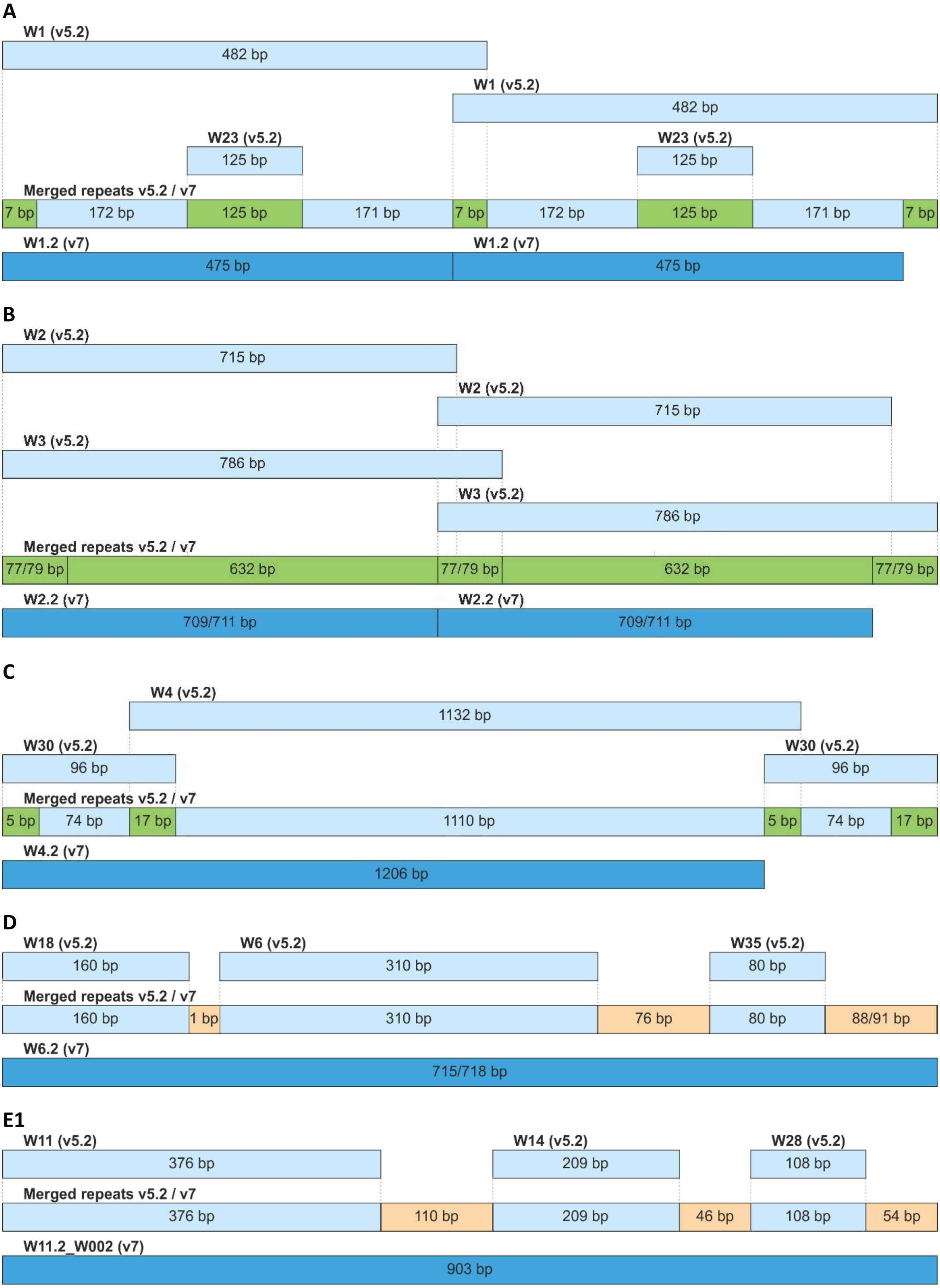

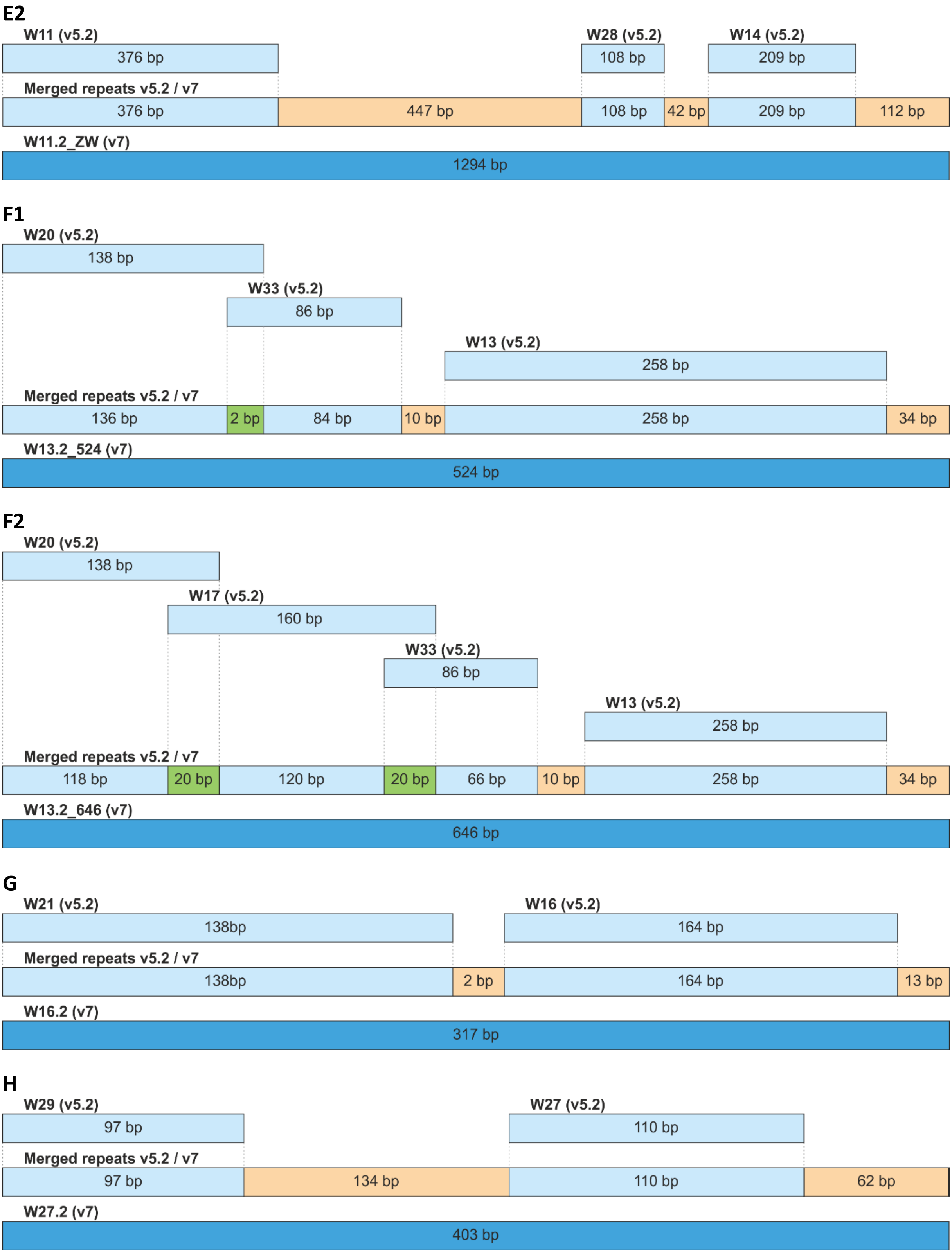
E2
W11 (v5.2)
376 bp
W28 (v5.2)
108 bp
W14 (v5.2)
209 bp
Merged repeats v5.2 / v7
376 bp
447 bp
108 bp
42 bp
209 bp
112 bp
W11.2_ZW (v7)
1294 bp
F1
W20 (v5.2)
138 bp
W33 (v5.2)
86 bp
W13 (v5.2)
258 bp
Merged repeats v5.2 / v7
136 bp
2 bp
84 bp
10 bp
258 bp
34 bp
W13.2_524 (v7)
524 bp
F2
W20 (v5.2)
138 bp
W17 (v5.2)
160 bp
W33 (v5.2)
86 bp
W13 (v5.2)
258 bp
Merged repeats v5.2 / v7
118 bp
20 bp
120 bp
20 bp
66 bp
10 bp
258 bp
34 bp
W13.2_646 (v7)
646 bp
G
W21 (v5.2)
138bp
W16 (v5.2)
164 bp
Merged repeats v5.2 / v7
138bp
2 bp
164 bp
13 bp
W16.2 (v7)
317 bp
H
W29 (v5.2)
97 bp
W27 (v5.2)
110 bp
Merged repeats v5.2 / v7
97 bp
134 bp
110 bp
62 bp
W27.2 (v7)
403 bp

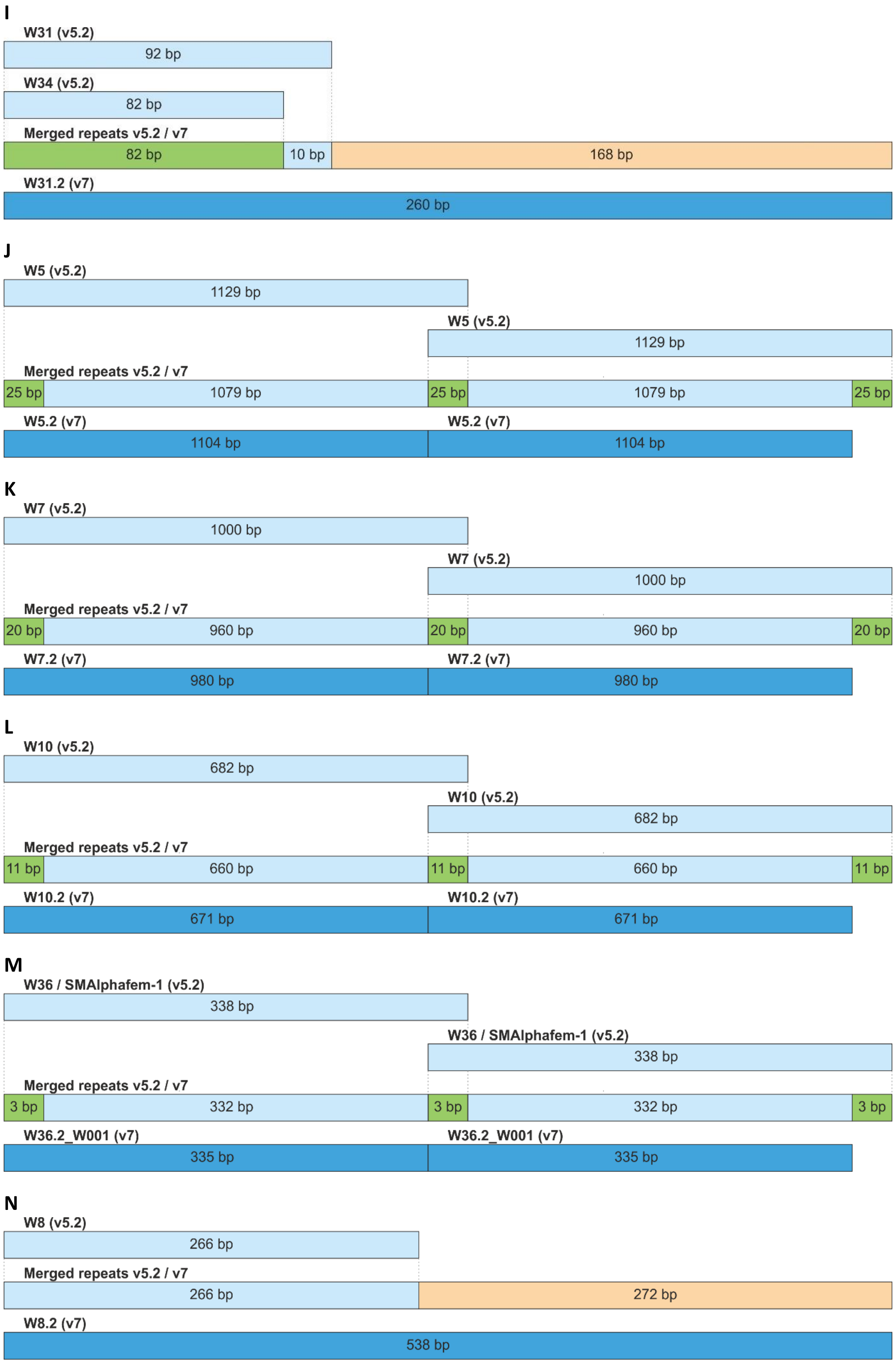
I
W31 (v5.2)
92 bp
W34 (v5.2)
82 bp
Merged repeats v5.2 / v7
82 bp
10 bp
168 bp
W31.2 (v7)
260 bp
J
W5 (v5.2)
1129 bp
W5 (v5.2)
1129 bp
Merged repeats v5.2 / v7
25 bp
1079 bp
25 bp
1079 bp
25 bp
W5.2 (v7)
1104 bp
W5.2 (v7)
1104 bp
K
W7 (v5.2)
1000 bp
W7 (v5.2)
1000 bp
Merged repeats v5.2 / v7
20 bp
960 bp
20 bp
960 bp
20 bp
W7.2 (v7)
980 bp
W7.2 (v7)
980 bp
L
W10 (v5.2)
682 bp
W10 (v5.2)
682 bp
Merged repeats v5.2 / v7
11 bp
660 bp
11 bp
660 bp
11 bp
W10.2 (v7)
671 bp
W10.2 (v7)
671 bp
M
W36 / SMAlphafem-1 (v5.2)
338 bp
W36 / SMAlphafem-1 (v5.2)
338 bp
Merged repeats v5.2 / v7
3 bp
332 bp
3 bp
332 bp
3 bp
W36.2_W001 (v7)
335 bp
W36.2_W001 (v7)
335 bp
N
W8 (v5.2)
266 bp
Merged repeats v5.2 / v7
266 bp
272 bp
W8.2 (v7)
538 bp

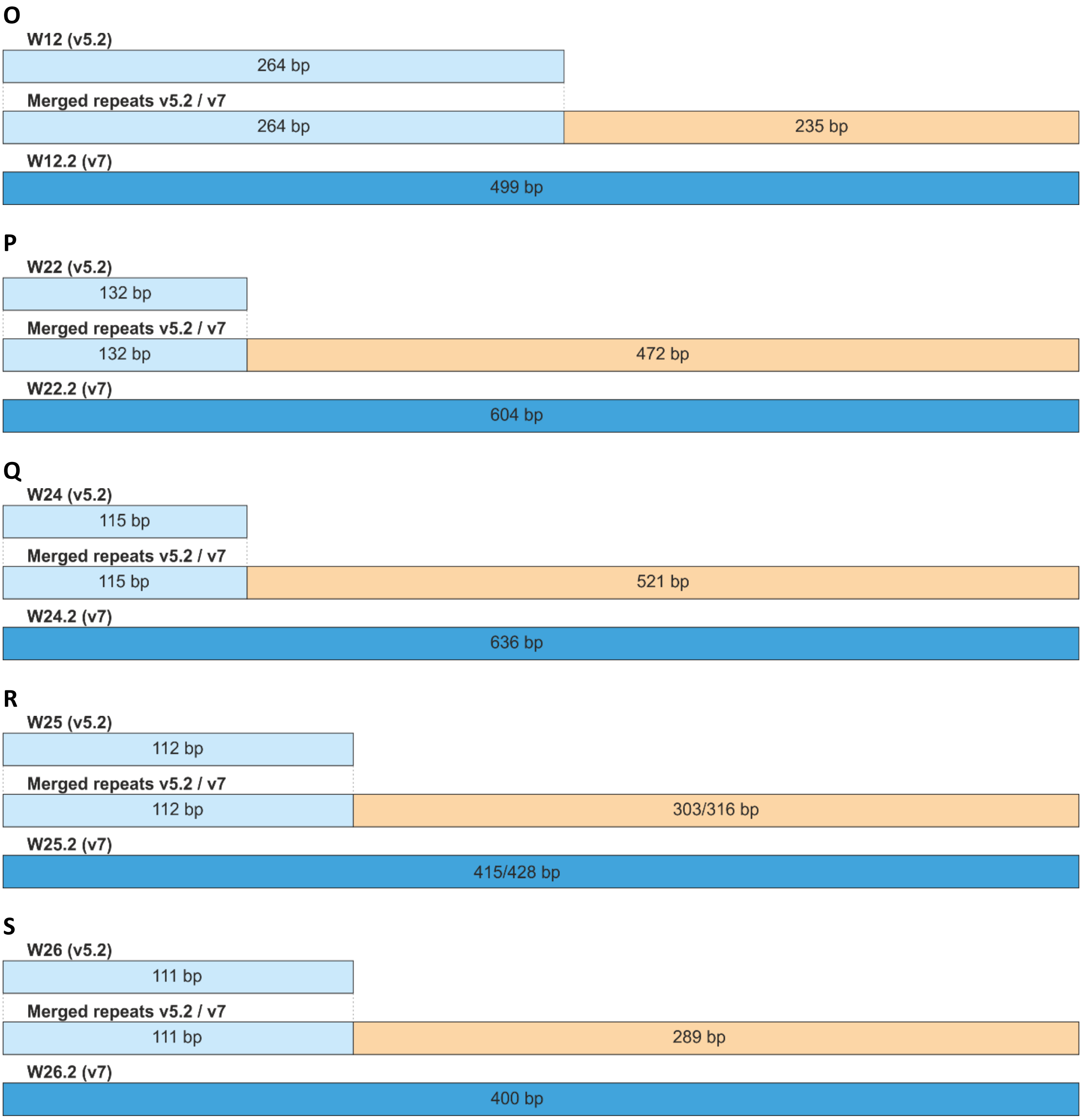

Suppl. Fig. 2: **A**, Summary of the structures of the newly defined 19 WEFs (A-S) based on BLASTn analyses and comparisons. To this end, the originally described 36 WEFs in genome version V5.2 of *S. mansoni* (Lepesant et al. 2012b) were compared with WE sequences updated in genome version V7. The original versions (V5.2), merged versions (V5.2/V7) and the final versions (V7) are given. The colors indicate former WEs (light blue), new additions in V7 (beige), overlapping regions (green), and the newly defined WEF versions (dark blue). In one case, W23 (see A), a WEF formerly described as individual repeat family was found in V7 to be part of another WEF, here W1.

Supplementary Figure 2.

B

Sequences of the 19 newly defined WEF

W1.2_SM_V7_W003

TCATTCAACAACATATAATTCTTTCTTCACACATATCACTGATCGAATGTCTTTGGAATATTTTGGAGTGAAATT
TGCGTTTCTCATTCTTATGAGCTTGATGACTCATGTGACAGGAATAAGATTTATGTGGATATCGACTGTGACAAT
GAGAATTGTGAATCGGATGTGCAGATGAGTTGTGCTGCGTACTTGTGTAGAGTTTCACAAATGTGTGATAATGCC
AATTCGAGTGTTTGTGGTTGCAATGACGTTCACACCTGGATTGAAGAGGCAGTGAGCGAATGTGATGATGCATTT
GATTGTGTGGTTGTGTTGGACCAATGTGCACATGGAATCGTTGCTTGTGCACATGGACCACCACAAATAACACAC
TCAATTCATTCTCCGTCTTCTCGAACAGGCATTGCCTACGAATCAAAAAAACTGTTTGCCTTATCATCTCGAACT
TTAATTTTTATTTCGAGAGTTAAAT

W2.2_1_SM_V7_W001_709bp

AATGTCACGTGGAAACTGATGTCCGAAAGAGCATAACACACAATTTCAGCGTTTCCCGATCTCAACCGAACGGGA
ATCGTTCGTGAACACACTTCACTGTTTATTGATTGAGGAAATGCGCAACGATTCTCGGCACTCGGCTCATCATTT
GAGCTGTTGACTGTTCAATCGTTGTGCATGATTGTCTTCTACTCATACTGATTGATCACTTGAATAGACATCGGC
AAACTGCACATCAGCAAGCTTACGCACTTGGTCGTCCAACGTGTGTACTCCTCTACTATGATGAACAACTGATTT
GCTATCTTCCGAGTATGATGCAGGTCCGAAAACAGCCCACCACAACAACGTTCTGACGGCGTACGCGTTTCGCCT
TACACACATTTCGTGGCCTGACACCCAACGTTCTTCCGACACGTCACAACCAATCACCAGTAGAACCTCTCCACA
ATCTACATCATCTAATTGTACATCACACAGATGCGGTCAATTCACCAGGTTGTTCGTTATCAACTTTGTTGACTT
ATGCACTCATACACATGCACAATCACAGCTCCTTCGATCATCACTTGTTCAGACCAATATGCAGAACATAGCTCA
ATAGACGCATTCGTAATTTTCGTCGTTCTATTACCACACACAGTGTGCATTTCCACCGACACTTAGATTTCCTTC
AACCCGACCGACCTCAACACAGTCTCACTTCATC

W2.2_1_SM_V7_W001_711bp

TCTTCTACTGATACTGATTTATCACTTGAATAGACATCGGCAAACTGCACATCAGCAAGCTCACGCACTTGGTCG
TCCAACGTGTGTACTCCTCTACTATGATGAACAACTCATTTGCTATCTTCCGAGTGTGGTGCAGCTCCGAAAACA
GACCACCACAACAACGTTCTGACGGCGTACGCGATTCGCCTTACACACATTTGGTCGCCTCACACCCAATGTTCT
TCCGACACGTCGCAACCAATCAGCAGTAGAACCTCTCCACAATCTACATTATCTAATTCTACATCACACAGATGC
GGTCAATTCACCAGGTTGTTCGTGATCAACTTTGTTGACTTATGCACTCATACACATGCACAATCACAGCTCCTT
CGATCATCACTTGTTCACACCAATATGCAGAACATAGCTCAATAGGCGCATTCGTAATTTTCCTCGTTCTATTAC
CACACACAGTGTGCATTTCCACCGACACTTAGATTTCCTTCAACACGACCGACCTCAACACAGTCTGACTTCATC
AATGTTACGTGGAAACTGATGTGCGAAAGAGCATAACACACAATTTCAGCGTTTCTCGATCTCAACCGAACGGGA
ATCGTTCGTGAACACACACTTCACTGTTTAGTGATTAAGGAAATGCGCAACGATTCTCGGCACTCGGCTCATCAT
TTCAGCTGTTGACTGTTGAATCGTTGTGCATGATTG

W4.2_SM_V7_W004

ATGATTCTCGAAGCTAAGTATAAGATGACTGAGAAAATTTACTGAGAAAAGTTGACCACGAAGAGAAGATCAGTA
ACGGTGAACGCTGGAAGAAGGAATCGAAATACCATGACTCGGTCATGGAGGCGTGGTCAACCCTCATAACTTCTT
GTGTTTCGTATCAAATATATTATTCTGTCCTCAATTCGAACGTGTCCTTCTGGAGTGCTAGGTTTCATAATGCTG
ATTGTCATGGTACGACGAGTCAGAGTAGAATGAGATGAGATCTCCTCGTTATATCTTATAGGTTATGTAGTCACA
TCGTGACAAATCTACAATTTAGCGATTGGGTCAATTATTCCATTAGCAACGATAAATGTACTAATATCGAACTAG
ACCATAATACTACAACCAAACTTTCATCAACTTGGTGTTTAAAAAGTACGTTAGACAACTAAATATTTAATCCCT
GACCATAGTTTCCACATATGATAGCAGTTTAACATATGTATGTATTTCAAGCATGATGCTATTGCTGCCACCAAG
TCCAGCAAGACATCCTACTTTCATATTACTAACCAATTACACTCTGTTCTGAGTAGTATTAATTTCAGTTATTTG
CTAGCTTATATATATTCATTGAATTGTTTTCTCGCCTTATTCGTTTCCTAATTCTTGCCTCCATTTCATTCATTT
GCAGTCTAATCCTATACTCTCGAATGCAAGAAATCAGATTACTGGCTGGAAATGTGATAGCATCTAATTCCAGCA
ATCTAGCTGTCCTGATATGAACGGAATTATATTGAAAAGATAACAACTCAGAACATCCTTATGAACGCCCTTGGT
TAAGGAATTTAGTTGACGCATAATGTAAATAAGTTGTTATTGTACGAATGACTCTACTCGTTAAATAAGTCAACA
AAAGGGCTGAACCAAATGAAACGTGAACAATCTCAAATGTACATGGACTGCAATACCTTTTTAGAATAAAGTTTA
CAGAAGTCTAAATGATTTCATCATGACAGGTGAATATGGGATCATCCAGGTCACCTCAATTAGGTGATTCTGTCT
GACACTCACTGGAGGACTTATATCAGATAATCGCAATATAATCGCTCATATAATAATTGACGCTTAGATGATATC

TTACCCCTTCACACTAAAGTTTCTAATGTCTGGGTGCTCACACAGACTGTGTGACCGTGTTTTGGTGTTGCTGCT
AACCAC

W5.2_SM_V7_W018

GGGTGCTGATAAATACTTCAAGTGCTTTGTCATGCATATTCATCAACAAGGTTCACATTCATACACCATGAATAA
CGTTGAGCTACCTGTTGTCCAGCCACATAATGGCCTGTGAGTCATCGTTGACCAAGACGTGAGAAATACTCAAAG
CTTCTGTATGATAGCCAACAAAGGTTCCACAATCTTCCAACCGTGTTGTTGTGAAAGCATTTTTAACTTTCTACA
TCGTGTTTGTTCGTCCTAAACCCGAGTAATGCACACAGGTGGCTGTCGCCTGTCCAAAATGATACAGTGAGCATT
TGCAAATGTTTCATGGAACCATCACTTAGCTGATTCTCGAGATCCACAAGCTCGCGTACGATGATAGACTGGCTG
AACTAATCCTATTTTCCTTGTCATAAAATAGGTGCACAAGTGACTTGATCACAGTTTCCAATTTCATTAATGATA
ACTTTGCATCTGACATGTCCTCATATTATTATTATTATGATTACTGTCATTATGATTATTCGTATTCTCAAACAG
AATTCACGAGGACACTGAGAATTATCACATGCACAGAACAAATCACTTATCACTTCACCATCAAATCATCGAATT
TTCACTCAGTGGAATCCATCATCTCACCACTCACGTGAAGCTCTGTCTGTCCACATTTTCACATCAATTTAGATC
ATTTGAGAGACTGGCATCGCTAGCACTAACGAAGGTCGCTCAGACTCCCGTCCTCTCCATACTCAAACCGCGTCT
CCCCTCTTCAGTCTCCTCACTGCATTTCAACGTGTCACGGCTCAAGTAGCATTTCATGTAGAGTTAACTGAGTCT
GTCCTATGTTTCCATTCTGATCTTGGTTCGACTTTCATGGTGTGCGCTCTCCCTTGTGATTAGTCAGAGAAACCG
TCATAGGGACAGTGTTATCCCTCTCATATATAAATCACCATCCTCGTCTGGCATCCTCATCATCATCATCATCAT
CATCATCATCATCATCGTTCTTGCTATATGCTGACTATGTGAAGATATGGAGAGCGATATGAAGTGAGGGTGGTA
GCTGAGAACTCACAAATGACCAGAAGAAATTATCTGGATGGTTCCAAACTTGAC

W6.2_SM_V7_W014_715bp

ACTCACTTGCAAATGATATGATACGAACGTTGTTGCAGCACTGGTCAATAGACCGTCTGTGATTGTTGACTGAAC
GAATTCAGTCTCACATTGTATTGTTGATGGTAACGCCGGTCACACACACTCGAATGATGAACTGCTGAAAACAAC
AGTGTTCATGAGTGTAATATACAGCTACAGAGGAAGCAAACAAAGTCACATTGATGACACAGAAACTTGACTCAA
TAGAATACTGAAAATGTCAATTCAATATATACCACACTGAAGGAAGTTGAGTTCCGTTGAAACGATTGGTAATAT
GAAGAAGATATTTTGTGAAGCAAAGTGTTCACTGCATATCTCATGCAAATGCTTGGTGTGAATACTGACAGCGAA
TGAAAAGAATTTGGCGTGTGTGATCAGCTGACATGTGAACGCGAACGATTGTGAATGAAATGGCAAAGAAATGAC
ACACTTGAAATATGTGAAGGGAATGGTGGAGATTGTTGAGCTGTGATTGACTTGATGAAATGATGAGTGTTGGAG
GACGGTTGAAGAGCTGGAAGCACTGGACGACCGATTGGTCGTTGCATGGGACTCGTGATCAGTGCGCCTCAGTGT
TATGACCCTGTTTGTGTTGAGTATCGTGTCTCAGTCTACAGCTCACAGTCGATGAACATATCCAACAAAAGTACA
CATGATCAATATTTCATATTATTATATTATTATCATTGTT

W6.2_SM_V7_W014_718bp

AGTCTCACATTGTATTGTTGATGGTAACGCCGGTCACACACACTCGAATGATGAACTGCTGAAAACAACAGTGTT
CATGAGTGTAATATACAGCTACAGAGGAAGCAAACAAAGTCACATTGATGACACAGAAACTTGACTCAATAGAAT
ACTGAAAATGTCAATTCAATATATACCACACTGAAGGAAGTTGAGTTCCGTTGAAACGATTGGTAATATGAAGAA
GATATTTTGTGAAGCAAAGTGTTCACTGCATATCTCATGCAAATGCTTGGTGTGAATACTGACAGCGAATGAAAA
GAATTTGGCGTGTGTGATCAGCTGACATGTGGACGCGAACGATTGTAAATGAAATGGCAAAGAAATGACACACTT
GAAATATGTGAAGGGAATGGTGGAGATTGTTGAGCTGTGATTGACTTGATGAAATGATGAGTGTTGGAGGACGGT
TGAAGAGCTGGAAGCACTGGACGACCGATTGGTCGTTGCATGGGACTCGTGATCAGTGCGCCTCAGTGTTATGAC
CCTGTTTGTGTTGAGTATCGTGTCTCAGTCTACAGCTCACAGTCGATGAACATATCCAACTAAAGTACACATGAT
CAATATTTCATTATTATTATTATTATATATCATTGTTACTCACTTGCAAATGATATGATACGAACGTTGTTGCAG
CACTGGTCAATAGACCGTCTGTGATTGTTGACTGAACGAATTC

W7.2_SM_V7_W016

TGCCGAGACAATGATGGATCGTGTTTACTGACAATGGAATATTTACCAGTGCTTCAACGACAACTTTCCTTATGG
ACGACTGAACACAGATGTGATGAAATGCTTGATATGTGGTCGAGCACAGAAACCGACTGACAAGATTTGCGCACA
ATTCCAGAACGCTCCAATTCTCATCATACAGTGGAGAATGTCATCACTTCACACAATGCATTGAATTCAGTTACT
GGTCGGATGTGAAGAGAACGCTAGATGAGGGTGTGAGAAATCGGTCAGTACAGAAATGTCAATCCCTATACAGAA
CAATCAATCATCCAATTGATTGAACTGTGAAATGTTCACGTGTGAGCCGAATGTTGACTGTCAGCTGACATTGAA
CACTATCAACGAAGTGATCTTCCACAATGCACTGAATGTCTGATGAACAATATTCATAAATCTGTGCTTCGTGTG
CACGACAGTCACAACAAGCAAATAGGTGATAGAGTACTCCAGTCAGTCCGTTCAACATCCACCTCTTCATTCCAG
TACTAATCACATTTTCATACACACCACTAAACTTCACTTTCACACACAATCTCACATTGGTGTAATCAAGCTGTG
CACAATACGTTCGACTGATTTGAGAACGGTGAAAATTCGTTGAAGTTTTGGAGGATAACATCCTTGTGTACAACA
GTGGTTGGTTTGTCCATTTACCGTGACCCATTTCAATTCGAAATACAATACAGTCCAGTCTACTCACCAACTTCT
CTATCCCTCACATAAACGTGCAAACATCACTACTTTCGAAAGAATACTCCAGCAGTATATAAATTTCCAATATTT

CACTTCACATTTCAACACCTTGATATTTGTCTCGTGGTGTTGTTTAGAAGTTGCAATATGTTCTCCGTTTTCCCT
CTTCATTGAATACACTGTGATAGCTGAACACCGTGGTACGAGTGCTTCTACAGTGTATTTGACGAGTACTTCATT
CGACA

W8.2_SM_V7_W015

ATTCACACATATATTATGTTCGACGAGTGAGTGATGAGAGAGAAGATTTCCATCACTTAATTGATCATAGTTCAA
ATTGTGCAATACATCTGTGAATTCAGATTCAATAAAATCGGTAACTAAATCACCTATGAATTCACACATATATTA
TGTTCGACGAGTGAGTGATGAGAGAGGAGATTTCCATCACGCACATCCTCATAGTTCGATTTGTGCAGGTGGTCT
GAGAATTTCGACACAATAAACTCAATCACAACATCACATATATATTTTCACTCATACAATACGTTTGACGAGTGG
CTGAAGATAGAAGAGAATTACTACACACAATTCATCATAGTTGAATCACTGCAATTGATGTGTCAAAATGGAATG
AATAAACTCAAACATTGCATCACCTTCGCATTCACACATACATTAAATTTGAAGAGTGAGAGATGAGAGAGGAGA
TTTCCATCACACAATTCATCATAGTTCGAATTGTGCAATACATCAGTGAATTCAGTTTTTATAAAATCGGTAACA
AAATCACATATGA

W10.2_SM_V7_W021

ACAACACCTACGAATTCACCCATACATGATTTTCAATAATTGAACGATGTCACATGACATTTACACGACATAATT
CATCACTTTCCATTTTGTGTAGGAGATCTCTGATTTTCGATCAATAACCTCAGTCGCAACTTCGCTCGTGAATTC
ACTCATACATTACCTTTGAGTTGTGACTGAAGAAAGGAGAGATTCCCTCCACTGAATTCAACATTGTTCCATTTA
TTCAGTTGATTTGTCGAAGGAGTTTATAGAATATCAAACGCTGCAGCATCCGTTCTTTCACTCATCCAATTTGCT
CGACGACTGATTGATGAGCAGGAAGATTTTCATCACGCAATTCATCACAGTTCAATTTGTGCAGTTGATGTGTAA
ATTGAGAATCACTAAACTCAGTCGCAACATCACCGGTAATTCAGCAATCCAATGTGTTCTACGAGTGGGTGATGA
TGTTGGGGGAGATTTCCATCACACAATTCATCATTCTTCAATACGTGCAGGCGTCCTACCAAACATCAATAATGC
AGATCAATCACCACATCATCATGTAATTTACCCATACATTATGTTGGAGGAGTGAACGATGAGAGAAGAGATTTA
CATCACACAATTCATCACAGTTCAATCACTGCAATCGATGTGTCAAACTAGAATCAATTATCGCAATCACA

W11.2_1_SM_V7_W002

CTAATCAAACACGTAGTATCCCTGTTCAAACTATTTCACTACACCGACTGAACATTTCATAGCACGAATCAATAT
TCAGTAATGTTGTATTCAATAATCAATCATTTTCACTTAAATATCTGTCAGAAGAAATAACATCAATGAAACCAC
CATGATGCAGGGCACACAGTTGTCAAGAATTTCACAGTCTCCACAATCAATATCCAAGCATAAATTGTTCATCTG
AAATACAACGAAGCAGTGCGTTCTACATGTTGATTCGATGAGAAGCGAAATATTGAAATTTTGTTCACTGAAATG
AGAAGATTGTTGAGAAAAGTGAATTGGACTTCGCAAATGAAATGTAGATCATTGGACACTATGGACTGCATTCAG
AATGCTTTCTGACTCGTAGTCGAACAGACATGTGAAACGGTTGGTCATTTTGAGGTTGAAATAATCAGAGCCGTA
TTTGTTCACTTGAAAACATGAAGATTGGTACAAGGAGGCACGAAATGTATATATATGCAGCACACATTGCGGTTG
GATTTGTCTGAGGACTGGAATACAGTGCGAGCGCCCGAACCGAATCAGGTGGATTTCTCAGGAGGCCACACCACC
AGCCTTTCACCTGAGGTGTAATCCACAAGGCAGTGAAGCAACGTCGGGCGATACGGTCCCATGGTAGACGGTGAC
CAACAATCGGTTTCATACGCCATTTATTCCCTCAACAGTTATAATCTAACACCTACACCTACAATGTTGAACATG
CAGTATGTGTCTGATTTGTAAAGATTTCAATCTTCTCGTCGCTAATCACAACGACTTCAACCGATCAATCCATTT
GGTCATGGGCGGCATATGACCACTGACAAATACTACGAAACGCCTGTCTTCCATTCTACGGTCAGTCAATAGAAA
ACT

W11.2_2_SM_V7_ZW

AAATGAAAAGTCACATGTGGAGATTCTGATTGGCTGATTAATTCACTGCTACTATGTTTAGCATTATTCTAGAAT
TTTCAGTAAAACGCAAGGACTTTAAAATACTATAAAAGTCCTATATTTTCTGTACATCAAACGAACCTTTTGAAG
TGAAGTGCTTCTCGCATATTTGTGCCTTTTCTCTCGTGTTCAAGTCGCTGTGTAGTTCTAGCTTGGGGGTCACGA
AAGTAGCTTAGGATCCGAATAATAGCGTTCAACCAACAAGACGTATCAGCGTATGAACCCGTGTTGGTCAACGAC
TACCATGGGACTGTATCGTCTAACGTTGCTCCACTGCCTTGTGGATCAGACCTTCACATCAAAGGCTCGTGGTGT
GGCCCCCTAAGAAAACCACCTGCTTCGGTTTCACGCACGAACACTATTCCAGTCCTCAGACAAATCCAATCACAA
TGTGTGCTGCATATACATTTGGTGCCTCCTTGTACCAATCTTCATGTTTTCTAGTAAATAAATACGGCACCTCTT
ATTTTAACCTCAAAATGACCAATCATTTCACATGTCGGCTCGACTACGAGTCACAGAGCATTCTCAATGCAGTCC
ATAATGTCAAATGATCTAGATGTAATTTGAGAAGTTCAATTTAGTTTTCTCAACAGTCTTCTCATTTCACTGAAC
AAAATTTCAATATTTCTCTTGTCTCATGAAATCAACATCTAAAACGCACTGCTCAGTTGTATTTCAGATGAACAA
TTTATGGTTGGATATTGATTGTAGAGACTATGAAATTCTTCACAACTGTGTGCCTCGCATTATAGTGGTTTCATT
TAAGTTATGCTGTTTGACAGATATTTAGGTAAAAATGAGCTATTATGGAATACAACACTACTGAATGATGATTCG
TGCTATAAAATGTTCAGTCGGTATAGTGAAATAGTTGAAACAGAGATACTATCTACGAGTTTTCTCTACAGTTTT
ATAGTGACTACCCGTAGAATGGAAGACACGCGTTTCGTAGTATTTGGGAGTCGTCATATGCCACCATTGTCCAAA
CGGAATGATCAATTGAAGTGGTTATCATTAACGACGAGGAGATTCAAACGTTTACAGATCCAAGGCACACTGCAT

GTTCAAAACGTGAGGTGCAAGTGTTACAATATAACTGTTGAGGGAAGAAATGGCGTATGTTGGTATGATCCTGAA
AATTTCTCGCAATCGACTTGATAAGCTCATGAAACATTGAGGAGCATTTCATTCAATCAGCGTTTGATTGGAAGT
TATGTTAGTAACATAGCGA

W12.2_SM_V7_W004_496bp

ACTGTTTTGATGAGAATTGTGAGGCGGTTGTGGAGATGAGAGGTGCTGCGTACCTGTGCATTGTGGCTGGTTGGT
GTTGTTGTGATGATGTTAGTGTGAGTGTTTGTGGTGGCAATGCTGTTCACACGTGGATTGAGGAGGCAGTGAGCA
AACGCGACGATGCATTTGAGTGTGTGGTTGTGCTGGACAAATGTGCACATGCAATCGCTGCATTTTCACATGCAC
CATCACAAATGCCACATTCAATTCATACTCCATCCTTTCAACCATGCATTGCGTCCTCATCACCACCACCACCAC
CACCACCACCACAGTTTGCATTATCAATTCACACATACAATTGCAAATTCAGCAGACAATTCATTCAACACAATA
TTCTTCCTTCACATATATCTGTGTTTCAATGTGATGGGAATATGTTTGAGTTGAACATGATTTAGTAATTGTGAT
GTGCGTGATGATGCATGTGACAGGTTTGCAGTTGACGTGGATGGTG

W12.2_SM_V7_W004_499bp

GTTAGTGTGAGTGTTTGTGGTGGCAATGCTGTTCACACGTGGATTGAGGAGGCAGTGAGCAAACGCGACGATGCA
TTTGAGTGTGTGGTTGTGCTGGACAAATGTGCACATGCAATCGCTGCATTTTCACATGCACCATCACAAATGCCA
CATTCAATTCATACTCCATCCTTTCAACCATGCATTGCGTCCTCATCACCACCACCACCACCACCACCACCACCA
CAGTTTGCATTATCAATTCACACATACAATTGCAAATTCAGCAGACAATTCATTCAACACAATATTCTTCCTTCA
CATATATCTGTGTTTCAATGTGATGGGAATATGTTTGAGTTGAACATGATTTAGTAATTGTGATGTGCGTGATGA
TGCATGTGACAGGTTTGCAGTTGACGTGGATGGTGACTGTTTTGATGAGAATTGTGAGGCGGTTGTGGAGATGAG
AGGTGCTGCGTACCTGTGCATTGTGGCTGGTTGGTGTTGTTGTGATGAT

W12.2_SM_V7_W004_475bp

GTGTTTGTGGATGCAATGGTGTTCACACGTGGATTGAAGAGGCAGTGAGCAAATGCGATGATGCATTTGAGTGTG
TGGTTGTGCTGGACCAATATGAACATGGAAATGTTGCTTCTGTACATGAACCATCACGAATAACACACTCAATTT
ATACTCCGTCCCTTCAACCATCGATTGCTTCCTCATCATCAACACGGTTTGCATTATCATTTGGAACATTGAGTT
GAATGTCGATCGGTGAATTCATTCAATAACATATACTTCTTCCTTCACACATATCTCTCATCGAAAATGTTTGTT
CGATTTGATAGTGATGTTTGCGTTTCTCATGGTTATGTGCGTGATGACTGATGTGACAGGAATGAGGTTTATGTG
GATATGGACTGTGACAATGAGAATTGTGAAGCGGATGTGCAGATGAAATGTGCTCCGTACTTGTGCATAGTGGCA
GTAATATGTGTTTATGTTATTTTGT

W13.2_SM_V7_W003

TCATCATCATTGTACATTCACACTCGGTGTCATCCATTGTGGCCGAATCACATTGCATTATCACATCAATTCACA
GCACCATCCCATTCACTTCAACTACATTCAAACATTCTCAATCCACATCACATCCAATTCACTCCACTCAACTCC
ATTGCAAACAAATCAACTAAACACAAACTAAATCATTTCAACTCAACTTATTTCATCTCAACAAAACACAACTCA
AGTCAAAACCAATGAAGTGAAGTGAAGTGAAGTGAAGTGAATTGGAGTGAACTCACCTCAACTGAATGTGTGCAC
AATGAACATGAGAGCCACACACGAGTGTGCTGCAACATCTCACTGGTCAGTAAATCCTGGCTAATCAAATCATCG
AGCATGGAGCATATTGAATGCTACACGTATCTCAGTTCATTCTCCACTGAACAGTACAGATCGACTGATTGTGAG
ACTAGGAATGCTTGCAACCACTTCACAACTCACACTACAACTTCCTATCAAGTCACTAGCTCTCAACTCAATTC

W16.2_SM_V7_W007

TATTGTGAAGATCAAATCAATAGACACTGATCAGTCATTACAATTCCATAATAATAAAGCTGTAAAATCATTTTC
TCTACATTATTAATAATAAAGTGATCATCACTCACTTTATTATTTAGTCAACGTCTTTTCAGGTTGTTCAAATTT
AAGGTTTCAGTACTGGATTTCGTACAGAAAACCACGTCATATAATGAACACTAATCTGTCAAACAAAAGTATATG
CAAAAATGTGTACGATCATTGCAATCTATTTTCACTTAGCGACCGTAGACGATTCTACTGAGATCAGTTCACTAG
TACAACAAGATAATCTC

W22.2_SM_V7_W008

CGCACCCACTCAATCACATCATTCACATATCTGTTCATTGTGAATGATTGGTATTCACGTTGGTAGTAGTTGCGA
AGATGTGCAGATCAGACACACAGACAGATGAACAGATCTCGTACAATCTGTGTGCTTCGAATGATGTCGATTTCA
CTACTACATCAATGAAGGATCCACTTGTAATGAGACGTTGAATAAACGAACGACTCATTGTGCAGCCACATTAGT
TCACTTGTTGTTTGTGTAGGAGTTTCGGCATCAATTCACGCTGTTCTCATTGCACAATGCGTATGTGTCAACACA
CCTCACATATGTGATTTTCATCAGTGACAATGCTTCCTACAACACAAATATCTCATTCATGTCTACTCATATTCA
CACGCTCGAACATTCGCTCATTTACTCAACATTGACAAACTCACTGACTCAATCATTCATCCACTCACTCACTGA
ATCACTCACACAATCAACAAGTCACTCACTCTCCCACTCACTCACTCACTCACTCACTCACTCACTCACTCACTC

ACACTCACTCACTTAATCACTCACTCACTCACTCACTCACTGATTCACACACTCACTCACTCAATCACTCACTCA
CTCA

W24.2_SM_V7_W020_636bp

ATATTTCTGATGAAATTGCTTACAAGCCTGAACATAAATTGCTGAATGAGTCAAATCATGATCAAAAACCATTCT
ACATAATACACAGTCTACATCCAATACACTGTCGAACAGACGTAAGATGAACTCAAGAGGAAGACGTTCAATCGA
TCACAGACACTGACACTGCAATTAAGGCTGTGTCGGATCAGCTGTTCACACGAATCAATGATTTCTTTGTATTGA
TGACTGTTTTCACTTTCAATTCATAATACAGTACATTCGTTCGTGCTCATCGAATACTGATTGATGAAATTGCGT
TATTGCTGGAATTGCTAGTTTATACTATAATTCAAATACTCTCAAACGTCACATTGGCGTCGCGATGGAGCCTGT
GATGAACCGGTTCGATACACATTCAAATGTAAATGCTTTCGATGATGACATGGAGAGGTTGGAAATCTGGACTAT
GACCAAGCAACATACTGAGTATTTCAATATTGTGGCTCATTTGCTCACTTTCATCAAAAATGAAGCATAGCAGTT
CAGAGTTGAATCAAACCTAGAATCACTGTGAGACAAAAGTTTCCGATCAATCAACTTCTTATCACATTTCTCGTG
TTATTGTACCAGATGCGGTTTGTCATAATGAGTCAC

W25.2_SM_V7_W012_428bp

CTACTGAATGCGTCTCACAATAGGACGAAACGACCGTCCAGTGCTTCCAGGATTACCCTGATGTTCTACCTTCAA
TTCACTCATTATTTCAAATATTAATATACTCAAATCACCACATATGCCTCTCCTCATACTAATAACATAATAATA
ATAATAATAATAATGAAATGCTCACCAGTGACCGACTTGAAGAGATATTTCCTTGAGTTCTAGTGAGAAGCAGTG
ACCAGTGGAGTTGAAACCACGTCTGTTGTGAGATATCAACTCAGTGAAGACAAATGGTGGAGGGATGGTGAACTT
CGTCGGTTGGTTGGAGTTAGACATAAACACCAGTGGATGCCGTCTCAGTGGTGATTGTTTTAAGTGTTCTGGTAC
GAGTCATGTTGTTCCTGTGTTGGAATGTGTTGAGGAGAGGAGGTGGATGCGCA

W25.2_SM_V7_W012_415bp

ATAATAATAATAATAATAATAATCAAATGCTCACCAATGACTGAATTGAAGAGATATTTCGTGGAGTTGTAGTGA
GAAGCAGTGACCAGTGGAGTTGAAGCCACGTCTGTTGTGAGATGTCAACTGACTGAAGACAATTGTGAACGGTTG
GTGAACTTGGTGGATTGGTTGGAAATAGACATAAACACCATGGGATGCCGTCTCAGTGGTGTATGTGTTGAGTGC
TCTATTGAGAAAGTTGTTATTCCTGTGTTGGAATCTCGTGAGGCGAAATCGTGGATTGGAAATGCTGAGGAGTCG
CACAATAGAACCAAATGACCGTCCAGTGTTTCCAGGATTTCCCTAGTGGTCTACCTTCAATCCACTCATGATTTC
AAATATAAATACACTCAAATCTCCACAAATGCCACTCCTC

W26.2_SM_V7_W010_400_bp

GCGGTTTGTTTTGAAAAATATCTCACTGAAGACATTGGTGGATGTGTCGCTGAATATCGTGGATCGGTTGAAGAT
GGAAATGAACACGGTTGGATGCCGACTCAGTGTTCTTGATGTTCTCAGCTTGCGCACGAAAATGATAAGCGCAGT
TTTCGAATCTGGAGAGGCGGTATCGTGGATGCACACTGCTGAGAAAGATATTAATGAAGTTCCAAGGCTGCTCAG
ATTGTCGAGGTTAACATGACGGTCTCTCAACAATTGATTCATCATCATAACCATTAACATTACGAGATTATCCAC
AAAACCACTTCTCATATTAGTCAACACGAGCTCACTAGTGACTGATTTCAACAGGTATTTCCTGGAGTTCTGGTG
AGAAGCAGAGACCAGTGCAGTTGAA

W26.2_SM_V7_W010_402bp

TGACGGTCTCTCAACAATTGATTCATCATCATAACCATTAACATTACGAGATTATCCACAAAACCACTTCTCATA
TCAGTGAACACGAGCTCACTAGTGACTGGATTCAATAGGTATTTCCTGGAGATCTGGTGAGAAGCAGTGACCAGT
TGAGTTGACGTACTTGTGTTTTGAGAGAGTAACTCACTGAAGACATTGATGGATGTGTCGCTGAATATCGTGGAT
CGGTTGAAGATGGAAATGAACACGGTTGGATGCCGACTCAGTGTTCTTGATGTTCTCAGCTTGCGCACGAAAATG
ATAAGCGCAGTTTTCGAATCTCGAGAGGCGGTATCGTGGATGCTCACTGCTGAGAAAGATATTAATGAAGTTCCA
AGGCTGCTCAGATTGTCGAGGTTAACA

W26.2_2_SM_V7_W003_401_bp

TTGTCGAGGTTCACATGACGGTCTCTCAACAATTGATTCATCATCATAACCATCAACATTACAAAATTATCCACA
AAACCACTTCTCATATCAGTGAACACGAGCTCACTAGTGACTGACTTCAACAGGTATTTTCTGGAGTTCTGGTGA
GAAGCAGTGACCAGTGGAGATGAAACAGGTATGTTATGAAAATTATCTCACTGAAGACATTGGTGGATGTGTCAG
TGAATATCGTGGATCGGTTGAAGATGGAAATGAACACGGTTGGATGCCAAATCAGTGTTCTTGATGTTGAGAGCT
CGCACACGAAAATGTGAAGCGCAGTTCTCGAATCTGGAGAGGCGGTATCGTGGATGCGCACTGCTGAGAAAGATG
GCAATAAACTTCAAAGGCTAATCGGA

W26.2_2_SM_V7_W003_399bp

ATATCGTGGATCGGTTGAAGATGGAAATGAACACGGTTGGATGCCGACTCAGTGGTCTAGATCTTCTCAGCTGCG
CACGAAAATGATAAGAGCAGAGTTCGAATCTGTGAGAGGAGGTATCGTGAATGCACACTGCTGAGAAAGATATCA
ATAAGGACAAAGGCCGCTCAGATGTCCAGGTTAACATCACGGTCTAGCAACAATTGATTCATCATCATAAACATT
AACATTACGAGATATATACACAAAACCACTTCTCATATTATTCAACACGTCTCACTAGTGACTGGATTCAATAGG
TATTTCCTGGAGATCTGGTGAGAAGCAGTGACCAGTTGAGTTGACGACTTGTGTTTTGAGAGAGTAACTCACTGA
AGACATTGGTGGATGTGTCGCTGA

W27.2_SM_V7_W017

TGAGATGGTCACTCAATGAAGACATTGGTGGATGTGTCGGTCATTTCAGTGAATCGTTTGAAGTAAGGCATTGAG
AGCGTTGGATGGTGACCCAGTGGTCTAGTGTTTAAGTGCTCACGCGCGAGACTGATAGGTGCTGGGTTGGAATCT
CGCGAGACTGCATCGTGGATGCGCACTGCTGAGCAGTCCCACAGTACGACGAAACGGCCATCCAGTGCTTCCAGG
TTTTCCATCGTGCTCCAGCTTCAATTCATTCATCATCTCAACTATCAACATTACTATAATATCTACGAAACGCCT
TCTCATCACTGTCAACATTTGCTCAACAGTGACTGGCTTGAAGAGGCATTTCCTGAAGTTCTCGTGAGAAGCGGT
GACCAGTGGATTGGAAGCAGGTGTGATG

W31.2_SM_V7_W004

TTGTTAGTTGTGAGTTAGTGGCGACGATGCGTGATATCCGGAATGAGTTTATCAGAAGTCACCTAATAATTAATA
TCATTTGTTTTGTATATGCAGCAGACGAAGTGTAGTGCTTACCTCATTGCTTTATTTTAAGACAATGGTATTGCG
ATGCAGTATGCAGTCTAAGAGTTCGTTGATCTACTCTGCAGTCGAAAAACATTTTCATATACGTTGGCGTAGTTT
GTGGACTGGTTATATTTTGTATGTTTTGTATTGGG

W36.2_1_SM_V7_W005

TTACATGCATGTTCTCACTGATGTTGAGCACTGCAATTGATCAGTGTGTTATTGACATATGTGCATAGTGTGCGT
ATTGCCTCGATAAAGCTTCAATTGACAAGCATTTAATGGGGAGATGGATAGTGGTTTTCAGTGGAATGCAGGATG
CGTGTTTCGTTCGATTTGCCACTGACCATCTGGATGTACCTGCATCTCGTGTTCATGTTCACTGTGACACTGGAA
CACAGTAACATTCACCTGAAACGCCATCAAGTTATCCACTCACCTACTGATTTCCCACAGCCACTCACTTGTGCA
AACTGGTCATGTTCAAATTCAATCAGCATTGT

W36.2_2_SM_V7_W001_333_bp

CCTGCATTCCACTCATAACCACTATCCATCTCTCCTTATAATGCTTGTGAATTGAGGCTATCTAGAGGCAATACG
CACACTTTGCACATATGTCAATAACAGACTGATCAATTGCAGTCCTCAACATCAATGAGAAGATGCAAGTAAACA
ATGCTGAGTGAATTTGAACATGACCAGTTTGCACAAGTGAGTGGCTGTGTGAAATCAGAAGGTGAGTGGATGTGT
TGATTGCGTTTGAAGCGAATGTTACTGCTTTCGAGTCTCACAGTGAACATGAACACGCAGATGCATGTTCATGTA
GACGACCATTCGCCAACAGGACGAAACACGCAT

W36.2_2_SM_V7_W001_335_bp

CGAGTGGACAACGCGATGGCGTTTGAAGCGAAAGCTACTGGGTTCGAGTCCCAGAGTGAACATCAACACTGAGAT
GCAGGTACATCCAGCTGACCAGTCGGAAATAGGACGAAACGCGCGTCCTGGATTCCACTGCTAGCCACCATCCAT
CTTTGCTTACCATGCTTGTGAATTTAGGCTATATCGAGGCAATACGCACAGTATGCACATATGACAATTACAGAC
TGACCAGTTGCAGTCCTAAACACATCAATGGGAAGATCCAAACAAACAATACTAAGTAAATTTCAACTTCACCCC
ATTGCACAAGCAAGTGGCTATCAGGACTCAGTGGC

Suppl. Fig. 2: **B**, sequence information of all newly defined 19 WEFs. All listed WEF sequences can be downloaded from zenodo (https://zenodo.org/) using the link https://doi.org/10.5281/zenodo.5482269.

Supplementary Figure 3.

WE sequences found on autosomes in males (A) and copy number variants (B)

A

>W2.2_SM_V7_W001_709bp

AATGTCACGTGGAAACTGATGTCCGAAAGAGCATAACACACAATTTCAGCGTTTCCCGATCTCAACCGAACGGGA
ATCGTTCGTGAACACACTTCACTGTTTATTGATTGAGGAAATGCGCAACGATTCTCGGCACTCGGCTCATCATTT
GAGCTGTTGACTGTTCAATCGTTGTGCATGATTGTCTTCTACTCATACTGATTGATCACTTGAATAGACATCGGC
AAACTGCACATCAGCAAGCTTACGCACTTGGTCGTCCAACGTGTGTACTCCTCTACTATGATGAACAACTGATTT
GCTATCTTCCGAGTATGATGCAGGTCCGAAAACAGCCCACCACAACAACGTTCTGACGGCGTACGCGTTTCGCCT
TACACACATTTCGTGGCCTGACACCCAACGTTCTTCCGACACGTCACAACCAATCACCAGTAGAACCTCTCCACA
ATCTACATCATCTAATTGTACATCACACAGATGCGGTCAATTCACCAGGTTGTTCGTTATCAACTTTGTTGACTT
ATGCACTCATACACATGCACAATCACAGCTCCTTCGATCATCACTTGTTCAGACCAATATGCAGAACATAGCTCA
ATAGACGCATTCGTAATTTTCGTCGTTCTATTACCACACACAGTGTGCATTTCCACCGACACTTAGATTTCCTTC
AACCCGACCGACCTCAACACAGTCTCACTTCATC

>W4.2_SM_V7_W004_1206bp

ATGATTCTCGAAGCTAAGTATAAGATGACTGAGAAAATTTACTGAGAAAAGTTGACCACGAAGAGAAGATCAGTA
ACGGTGAACGCTGGAAGAAGGAATCGAAATACCATGACTCGGTCATGGAGGCGTGGTCAACCCTCATAACTTCTT
GTGTTTCGTATCAAATATATTATTCTGTCCTCAATTCGAACGTGTCCTTCTGGAGTGCTAGGTTTCATAATGCTG
ATTGTCATGGTACGACGAGTCAGAGTAGAATGAGATGAGATCTCCTCGTTATATCTTATAGGTTATGTAGTCACA
TCGTGACAAATCTACAATTTAGCGATTGGGTCAATTATTCCATTAGCAACGATAAATGTACTAATATCGAACTAG
ACCATAATACTACAACCAAACTTTCATCAACTTGGTGTTTAAAAAGTACGTTAGACAACTAAATATTTAATCCCT
GACCATAGTTTCCACATATGATAGCAGTTTAACATATGTATGTATTTCAATAAATAAGTTGTTATTGTACGAATG
ACTCTACTCGTTAAATAAGTCAACAAAAGGGCTGAGCATGATGCTATTGCTGCCACCAAGTCCAGCAAGACATCC
TACTTTCATATTACTAACCAATTACACTCTGTTCTGAGTAGTATTAATTTCAGTTATTTGCTAGCTTATATATAT
TCATTGAATTGTTTTCTCGCCTTATTCGTTTCCTAATTCTTGCCTCCATTTCATTCATTTGCAGTCTAATCCTAT
ACTCTCGAATGCAAGAAATCAGATTACTGGCTGGAAATGTGATAGCATCTAATTCCAGCAATCTAGCTGTCCTGA
TATGAACGGAATTATATTGAAAAGATAACAACTCAGAACATCCTTATGAACGCCCTTGGTTAAGGAATTTAGTTG
ACGCATAATGTAAATAAGTTGTTATTGTACGAATGACTCTACTCGTTAAATAAGTCAACAAAAGGGCTGAACCAA
ATGAAACGTGAACAATCTCAAATGTACATGGACTGCAATACCTTTTTAGAATAAAGTTTACAGAAGTCTAAATGA
TTTCATCATGACAGGTGAATATGGGATCATCCAGGTCACCTCAATTAGGTGATTCTGTCTGACACTCACTGGAGG
ACTTATATCAGATAATCGCAATATAATCGCTCATATAATAATTGACGCTTAGATGATATCTTACCCCTTCACACT
AAAGTTTCTAATGTCTGGGTGCTCACACAGACTGTGTGACCGTGTTTTGGTGTTGCTGCTAACCAC

>W5.2_SM_V7_W018_1104bp

GGGTGCTGATAAATACTTCAAGTGCTTTGTCATGCATATTCATCAACAAGGTTCACATTCATACACCATGAATAA
CGTTGAGCTACCTGTTGTCCAGCCACATAATGGCCTGTGAGTCATCGTTGACCAAGACGTGAGAAATACTCAAAG
CTTCTGTATGATAGCCAACAAAGGTTCCACAATCTTCCAACCGTGTTGTTGTGAAAGCATTTTTAACTTTCTACA
TCGTGTTTGTTCGTCCTAAACCCGAGTAATGCACACAGGTGGCTGTCGCCTGTCCAAAATGATACAGTGAGCATT
TGCAAATGTTTCATGGAACCATCACTTAGCTGATTCTCGAGATCCACAAGCTCGCGTACGATGATAGACTGGCTG
AACTAATCCTATTTTCCTTGTCATAAAATAGGTGCACAAGTGACTTGATCACAGTTTCCAATTTCATTAATGATA
ACTTTGCATCTGACATGTCCTCATATTATTATTATTATGATTACTGTCATTATGATTATTCGTATTCTCAAACAG
AATTCACGAGGACACTGAGAATTATCACATGCACAGAACAAATCACTTATCACTTCACCATCAAATCATCGAATT
TTCACTCAGTGGAATCCATCATCTCACCACTCACGTGAAGCTCTGTCTGTCCACATTTTCACATCAATTTAGATC
ATTTGAGAGACTGGCATCGCTAGCACTAACGAAGGTCGCTCAGACTCCCGTCCTCTCCATACTCAAACCGCGTCT
CCCCTCTTCAGTCTCCTCACTGCATTTCAACGTGTCACGGCTCAAGTAGCATTTCATGTAGAGTTAACTGAGTCT
GTCCTATGTTTCCATTCTGATCTTGGTTCGACTTTCATGGTGTGCGCTCTCCCTTGTGATTAGTCAGAGAAACCG
TCATAGGGACAGTGTTATCCCTCTCATATATAAATCACCATCCTCGTCTGGCATCCTCATCATCATCATCATCAT
CATCATCATCATCATCGTTCTTGCTATATGCTGACTATGTGAAGATATGGAGAGCGATATGAAGTGAGGGTGGTA
GCTGAGAACTCACAAATGACCAGAAGAAATTATCTGGATGGTTCCAAACTTGAC

>W6.2_SM_V7_W014_718bp

AGTCTCACATTGTATTGTTGATGGTAACGCCGGTCACACACACTCGAATGATGAACTGCTGAAAACAACAGTGTT
CATGAGTGTAATATACAGCTACAGAGGAAGCAAACAAAGTCACATTGATGACACAGAAACTTGACTCAATAGAAT
ACTGAAAATGTCAATTCAATATATACCACACTGAAGGAAGTTGAGTTCCGTTGAAACGATTGGTAATATGAAGAA
GATATTTTGTGAAGCAAAGTGTTCACTGCATATCTCATGCAAATGCTTGGTGTGAATACTGACAGCGAATGAAAA
GAATTTGGCGTGTGTGATCAGCTGACATGTGGACGCGAACGATTGTAAATGAAATGGCAAAGAAATGACACACTT
GAAATATGTGAAGGGAATGGTGGAGATTGTTGAGCTGTGATTGACTTGATGAAATGATGAGTGTTGGAGGACGGT

TGAAGAGCTGGAAGCACTGGACGACCGATTGGTCGTTGCATGGGACTCGTGATCAGTGCGCCTCAGTGTTATGAC
CCTGTTTGTGTTGAGTATCGTGTCTCAGTCTACAGCTCACAGTCGATGAACATATCCAACTAAAGTACACATGAT
CAATATTTCATTATTATTATTATTATATATCATTGTTACTCACTTGCAAATGATATGATACGAACGTTGTTGCAG
CACTGGTCAATAGACCGTCTGTGATTGTTGACTGAACGAATTC

>W11.2_1_SM_V7_W002_903bp
CTAATCAAACACGTAGTATCCCTGTTCAAACTATTTCACTACACCGACTGAACATTTCATAGCACGAATCAATAT
TCAGTAATGTTGTATTCAATAATCAATCATTTTCACTTAAATATCTGTCAGAAGAAATAACATCAATGAAACCAC
CATGATGCAGGGCACACAGTTGTCAAGAATTTCACAGTCTCCACAATCAATATCCAAGCATAAATTGTTCATCTG
AAATACAACGAAGCAGTGCGTTCTACATGTTGATTCGATGAGAAGCGAAATATTGAAATTTTGTTCACTGAAATG
AGAAGATTGTTGAGAAAAGTGAATTGGACTTCGCAAATGAAATGTAGATCATTGGACACTATGGACTGCATTCAG
AATGCTTTCTGACTCGTAGTCGAACAGACATGTGAAACGGTTGGTCATTTTGAGGTTGAAATAATCAGAGCCGTA
TTTGTTCACTTGAAAACATGAAGATTGGTACAAGGAGGCACGAAATGTATATATATGCAGCACACATTGCGGTTG
GATTTGTCTGAGGACTGGAATACAGTGCGAGCGCCCGAACCGAATCAGGTGGATTTCTCAGGAGGCCACACCACC
AGCCTTTCACCTGAGGTGTAATCCACAAGGCAGTGAAGCAACGTCGGGCGATACGGTCCCATGGTAGACGGTGAC
CAACAATCGGTTTCATACGCCATTTATTCCCTCAACAGTTATAATCTAACACCTACACCTACAATGTTGAACATG
CAGTATGTGTCTGATTTGTAAAGATTTCAATCTTCTCGTCGCTAATCACAACGACTTCAACCGATCAATCCATTT
GGTCATGGGCGGCATATGACCACTGACAAATACTACGAAACGCCTGTCTTCCATTCTACGGTCAGTCAATAGAAA
ACT

>W11.2_2_SM_V7_ZW_1294bp
AAATGAAAAGTCACATGTGGAGATTCTGATTGGCTGATTAATTCACTGCTACTATGTTTAGCATTATTCTAGAAT
TTTCAGTAAAACGCAAGGACTTTAAAATACTATAAAAGTCCTATATTTTCTGTACATCAAACGAACCTTTTGAAG
TGAAGTGCTTCTCGCATATTTGTGCCTTTTCTCTCGTGTTCAAGTCGCTGTGTAGTTCTAGCTTGGGGGTCACGA
AAGTAGCTTAGGATCCGAATAATAGCGTTCAACCAACAAGACGTATCAGCGTATGAACCCGTGTTGGTCAACGAC
TACCATGGGACTGTATCGTCTAACGTTGCTCCACTGCCTTGTGGATCAGACCTTCACATCAAAGGCTCGTGGTGT
GGCCCCCTAAGAAAACCACCTGCTTCGGTTTCACGCACGAACACTATTCCAGTCCTCAGACAAATCCAATCACAA
TGTGTGCTGCATATACATTTGGTGCCTCCTTGTACCAATCTTCATGTTTTCTAGTAAATAAATACGGCACCTCTT
ATTTTAACCTCAAAATGACCAATCATTTCACATGTCGGCTCGACTACGAGTCACAGAGCATTCTCAATGCAGTCC
ATAATGTCAAATGATCTAGATGTAATTTGAGAAGTTCAATTTAGTTTTCTCAACAGTCTTCTCATTTCACTGAAC
AAAATTTCAATATTTCTCTTGTCTCATGAAATCAACATCTAAAACGCACTGCTCAGTTGTATTTCAGATGAACAA
TTTATGGTTGGATATTGATTGTAGAGACTATGAAATTCTTCACAACTGTGTGCCTCGCATTATAGTGGTTTCATT
TAAGTTATGCTGTTTGACAGATATTTAGGTAAAAATGAGCTATTATGGAATACAACACTACTGAATGATGATTCG
TGCTATAAAATGTTCAGTCGGTATAGTGAAATAGTTGAAACAGAGATACTATCTACGAGTTTTCTCTACAGTTTT
ATAGTGACTACCCGTAGAATGGAAGACACGCGTTTCGTAGTATTTGGGAGTCGTCATATGCCACCATTGTCCAAA
CGGAATGATCAATTGAAGTGGTTATCATTAACGACGAGGAGATTCAAACGTTTACAGATCCAAGGCACACTGCAT
GTTCAAAACGTGAGGTGCAAGTGTTACAATATAACTGTTGAGGGAAGAAATGGCGTATGTTGGTATGATCCTGAA
AATTTCTCGCAATCGACTTGATAAGCTCATGAAACATTGAGGAGCATTTCATTCAATCAGCGTTTGATTGGAAGT
TATGTTAGTAACATAGCGA

>W12.2_SM_V7_W004_499bp
GTTAGTGTGAGTGTTTGTGGTGGCAATGCTGTTCACACGTGGATTGAGGAGGCAGTGAGCAAACGCGACGATGCA
TTTGAGTGTGTGGTTGTGCTGGACAAATGTGCACATGCAATCGCTGCATTTTCACATGCACCATCACAAATGCCA
CATTCAATTCATACTCCATCCTTTCAACCATGCATTGCGTCCTCATCACCACCACCACCACCACCACCACCACCA
CAGTTTGCATTATCAATTCACACATACAATTGCAAATTCAGCAGACAATTCATTCAACACAATATTCTTCCTTCA
CATATATCTGTGTTTCAATGTGATGGGAATATGTTTGAGTTGAACATGATTTAGTAATTGTGATGTGCGTGATGA
TGCATGTGACAGGTTTGCAGTTGACGTGGATGGTGACTGTTTTGATGAGAATTGTGAGGCGGTTGTGGAGATGAG
AGGTGCTGCGTACCTGTGCATTGTGGCTGGTTGGTGTTGTTGTGATGAT

>W16.2_SM_V7_W007_317bp
TATTGTGAAGATCAAATCAATAGACACTGATCAGTCATTACAATTCCATAATAATAAAGCTGTAAAATCATTTTC
TCTACATTATTAATAATAAAGTGATCATCACTCACTTTATTATTTAGTCAACGTCTTTTCAGGTTGTTCAAATTT
AAGGTTTCAGTACTGGATTTCGTACAGAAAACCACGTCATATAATGAACACTAATCTGTCAAACAAAAGTATATG
CAAAAATGTGTACGATCATTGCAATCTATTTTCACTTAGCGACCGTAGACGATTCTACTGAGATCAGTTCACTAG
TACAACAAGATAATCTC

>W22.2_SM_V7_W008_604bp
CGCACCCACTCAATCACATCATTCACATATCTGTTCATTGTGAATGATTGGTATTCACGTTGGTAGTAGTTGCGA

AGATGTGCAGATCAGACACACAGACAGATGAACAGATCTCGTACAATCTGTGTGCTTCGAATGATGTCGATTTCA
CTACTACATCAATGAAGGATCCACTTGTAATGAGACGTTGAATAAACGAACGACTCATTGTGCAGCCACATTAGT
TCACTTGTTGTTTGTGTAGGAGTTTCGGCATCAATTCACGCTGTTCTCATTGCACAATGCGTATGTGTCAACACA
CCTCACATATGTGATTTTCATCAGTGACAATGCTTCCTACAACACAAATATCTCATTCATGTCTACTCATATTCA
CACGCTCGAACATTCGCTCATTTACTCAACATTGACAAACTCACTGACTCAATCATTCATCCACTCACTCACTGA
ATCACTCACACAATCAACAAGTCACTCACTCTCCCACTCACTCACTCACTCACTCACTCACTCACTCACTCACTC
ACACTCACTCACTTAATCACTCACTCACTCACTCACTCACTGATTCACACACTCACTCACTCAATCACTCACTCA
CTCA

>W24.2_SM_V7_W020_636bp
ATATTTCTGATGAAATTGCTTACAAGCCTGAACATAAATTGCTGAATGAGTCAAATCATGATCAAAAACCATTCT
ACATAATACACAGTCTACATCCAATACACTGTCGAACAGACGTAAGATGAACTCAAGAGGAAGACGTTCAATCGA
TCACAGACACTGACACTGCAATTAAGGCTGTGTCGGATCAGCTGTTCACACGAATCAATGATTTCTTTGTATTGA
TGACTGTTTTCACTTTCAATTCATAATACAGTACATTCGTTCGTGCTCATCGAATACTGATTGATGAAATTGCGT
TATTGCTGGAATTGCTAGTTTATACTATAATTCAAATACTCTCAAACGTCACATTGGCGTCGCGATGGAGCCTGT
GATGAACCGGTTCGATACACATTCAAATGTAAATGCTTTCGATGATGACATGGAGAGGTTGGAAATCTGGACTAT
GACCAAGCAACATACTGAGTATTTCAATATTGTGGCTCATTTGCTCACTTTCATCAAAAATGAAGCATAGCAGTT
CAGAGTTGAATCAAACCTAGAATCACTGTGAGACAAAAGTTTCCGATCAATCAACTTCTTATCACATTTCTCGTG
TTATTGTACCAGATGCGGTTTGTCATAATGAGTCAC

>W25.2_SM_V7_W012_428bp
CTACTGAATGCGTCTCACAATAGGACGAAACGACCGTCCAGTGCTTCCAGGATTACCCTGATGTTCTACCTTCAA
TTCACTCATTATTTCAAATATTAATATACTCAAATCACCACATATGCCTCTCCTCATACTAATAACATAATAATA
ATAATAATAATAATGAAATGCTCACCAGTGACCGACTTGAAGAGATATTTCCTTGAGTTCTAGTGAGAAGCAGTG
ACCAGTGGAGTTGAAACCACGTCTGTTGTGAGATATCAACTCAGTGAAGACAAATGGTGGAGGGATGGTGAACTT
CGTCGGTTGGTTGGAGTTAGACATAAACACCAGTGGATGCCGTCTCAGTGGTGATTGTTTTAAGTGTTCTGGTAC
GAGTCATGTTGTTCCTGTGTTGGAATGTGTTGAGGAGAGGAGGTGGATGCGCA

>W26.2_SM_V7_W010_400_bp
GCGGTTTGTTTTGAAAAATATCTCACTGAAGACATTGGTGGATGTGTCGCTGAATATCGTGGATCGGTTGAAGAT
GGAAATGAACACGGTTGGATGCCGACTCAGTGTTCTTGATGTTCTCAGCTTGCGCACGAAAATGATAAGCGCAGT
TTTCGAATCTGGAGAGGCGGTATCGTGGATGCACACTGCTGAGAAAGATATTAATGAAGTTCCAAGGCTGCTCAG
ATTGTCGAGGTTAACATGACGGTCTCTCAACAATTGATTCATCATCATAACCATTAACATTACGAGATTATCCAC
AAAACCACTTCTCATATTAGTCAACACGAGCTCACTAGTGACTGATTTCAACAGGTATTTCCTGGAGTTCTGGTG
AGAAGCAGAGACCAGTGCAGTTGAA

>W27.2_SM_V7_W017_403bp
TGAGATGGTCACTCAATGAAGACATTGGTGGATGTGTCGGTCATTTCAGTGAATCGTTTGAAGTAAGGCATTGAG
AGCGTTGGATGGTGACCCAGTGGTCTAGTGTTTAAGTGCTCACGCGCGAGACTGATAGGTGCTGGGTTGGAATCT
CGCGAGACTGCATCGTGGATGCGCACTGCTGAGCAGTCCCACAGTACGACGAAACGGCCATCCAGTGCTTCCAGG
TTTTCCATCGTGCTCCAGCTTCAATTCATTCATCATCTCAACTATCAACATTACTATAATATCTACGAAACGCCT
TCTCATCACTGTCAACATTTGCTCAACAGTGACTGGCTTGAAGAGGCATTTCCTGAAGTTCTCGTGAGAAGCGGT
GACCAGTGGATTGGAAGCAGGTGTGATG

>W36.2_1_SM_V7_W005_332bp
TTACATGCATGTTCTCACTGATGTTGAGCACTGCAATTGATCAGTGTGTTATTGACATATGTGCATAGTGTGCGT
ATTGCCTCGATAAAGCTTCAATTGACAAGCATTTAATGGGGAGATGGATAGTGGTTTTCAGTGGAATGCAGGATG
CGTGTTTCGTTCGATTTGCCACTGACCATCTGGATGTACCTGCATCTCGTGTTCATGTTCACTGTGACACTGGAA
CACAGTAACATTCACCTGAAACGCCATCAAGTTATCCACTCACCTACTGATTTCCCACAGCCACTCACTTGTGCA
AACTGGTCATGTTCAAATTCAATCAGCATTGT

>W36.2_2_SM_V7_W001_335bp
CGAGTGGACAACGCGATGGCGTTTGAAGCGAAAGCTACTGGGTTCGAGTCCCAGAGTGAACATCAACACTGAGAT
GCAGGTACATCCAGCTGACCAGTCGGAAATAGGACGAAACGCGCGTCCTGGATTCCACTGCTAGCCACCATCCAT
CTTTGCTTACCATGCTTGTGAATTTAGGCTATATCGAGGCAATACGCACAGTATGCACATATGACAATTACAGAC
TGACCAGTTGCAGTCCTAAACACATCAATGGGAAGATCCAAACAAACAATACTAAGTAAATTTCAACTTCACCCC
ATTGCACAAGCAAGTGGCTATCAGGACTCAGTGGC

Suppl. Fig. 3A: Analyses of WEs of the newly defined WEFs and of WE-transcript sequences detected in males. We used these sequences for BLASTn analyses against V7 in the NCBI database, which allowed assigning hits to individual chromosomes. Shown are all WEFs being expressed as full-length or partial transcripts in males. Black letters mark the complete WE sequences, purple letters indicate transcript sequences identified in autosomal regions, green letters combined with purple letters in W26.2.and W27.2 indicate that for these WEs, we found full-length (green and purple) and partial transcripts (purple) (see also Tab. 2). Grey-shaded areas indicate the sequence segments that appeared as partial transcripts in males.

Supplementary Figure 3B

See separate Excel file, which contains a list of copy number variants of WEF on autosomes. In addition to numbers, 3D graphical overviews are provided with copy numbers, copy sizes, and their autosomal occurrence on chromosomes 1-7 (1-7).

Supplementary Figure 4.

Similarities between individual WEFs and mobile genetic elements

WEF	Description	Max Score	Total Score	Query Cover	E- value	Percent Identity	Accession
W2.2	TPA: Schistosoma mansoni Saci-1 LTR retrotransposon mRNA, complete sequence	130	130	85%	3E-25	65.42%	BK004068.1
W4.2	Schistosoma mansoni LTR-retrotransposon Boudicca, partial sequence	58,1	58,1	3%	0,002	88.64%	AY308024.1
W5.2	TPA: Schistosoma mansoni Perere-2 non-LTR retrotransposon	109	109	10%	4E-19	80.00%	BN000793.1
W11.2_ZW	Schistosoma mansoni clone F04 Perere 3 retrotransposon mRNA, partial sequence	152	152	11%	4E-32	84.00%	AY838777.1
W16.2	TPA: Schistosoma mansoni transposon Curupira-1	233	460	100%	1E-56	83.80%	BN001525.1
W36.2_335	Schistosoma mansoni repetitive DNA element t-2 of SM(alpha) family	499	499	84%	4e-137	98.58%	X15618.1

```
Query: W2.2_SM_V7_W001_711bp Query ID: lcl|Query_25507 Length: 711

>TPA_exp: Schistosoma mansoni Saci-1 LTR retrotransposon mRNA, complete sequence
Sequence ID: BK004068.1 Length: 5980
Range 1: 1438 to 2044

Score:130 bits(143), Expect:2e-25,
Identities:401/613(65%),  Gaps:12/613(1%), Strand: Plus/Minus

Query  1     TCTTCTACTGATACTGATTTATCACTTGAATAGACATCGGCAAACTGCACATCAGCAAGC  60
             || ||||||||    |||||||| |||||||| ||||| || || |  || |||  ||||
Sbjct  2044  TCCTCTACTGACGGCGATTTATCGCTTGAATATACATCAGCGAATTCTACGTCATAAAGC  1985

Query  61    TCACGCACTTGGTCGTCCAACGTGTGTACTCCTCTACTATGATGAACAACTCATTTGCTA  120
             | ||| | ||| || || ||||| ||||     ||  ||  || |  ||||  |||  | 
Sbjct  1984  TTACGGAATTGATCTTCTAACGTCTGTAGCTTACTCATACAATTACTAACTTTTTTCTTC  1925
```

```
Query  121    TCTTCCGAGTGTGGTGCAGCTCCGAAAACAGACCACCACAACAACGTTCTGACGGCGTAC  180
                | ||||   || || || ||| || || | |||||  | ||||||| | || || ||
Sbjct  1924   AATCCCGAAAATGATGTAGGTCCAAACACGGTCCACCCGAGCAACGTTTTTACTGCATAT  1865

Query  181    GCGATTCGCCTTACAC---ACATTTGGTCGCCTCACACCCAATGTTCTTCCGACACGTCG  237
              |   ||   ||| |||   ||  ||| |||  |   ||||| ||  ||||||  || ||
Sbjct  1864   GGATTTTTTCTTCCACCTAACCGTTGATCGAGT---ACCCAGTGGGCTTCCGGGACATCA  1808

Query  238    CAACCAATCAGCAGTAGAACCTCTCCACAATCTACATTATCTAATTCTACATCACACAGA  297
              ||||||||||| |  | ||| || ||| |||||| |   |||||   |||||| | || |
Sbjct  1807   CAACCAATCAGTAACACAACTTCGCCAGAATCTATAACTTCTAAAGGTACATCGCTCAAA  1748

Query  298    TGCGGTCAATTCACCAGGTTGTTCGTGATCAACTTTGTTGACTTATGCACTCATACACAT  357
              ||||| || ||||| ||| || || ||||     || || | ||||||| |    ||
Sbjct  1747   TGCGGCCACTTCACTAGGCTGCTCATGATTGTTTTCGTCGGCTTATGCCCAGGTATTTGC  1688

Query  358    G-CACAATCACAGCTCCTTCGATCATCACTTGTTCACACCAATATGCAGAACATAGCTCA  416
| ||| | ||  |||||||  ||  | || || |||      || |    ||| | | ||||
Sbjct  1687   GACACTACCAGGGCTCCTTGAATTTTAACGTGCTCAGTTTGATCTAAGGAATACACCTCA  1628

Query  417    ATAGGCGCATTCGTAATTTTCCTCGTTCTATTACCACACACAGTGTGCATTTCCACCGAC  476
              | ||| |  ||  | | |     | |   |||| || | ||| || || |   ||||||||
Sbjct  1627   AAAGGTGTCTTTATCACTCGTGTTGCCTTATTGCCGCTCACGGTTTGTACAACCACCGAC  1568

Query  477    -ACTTAGAT-TTCCTTCAACACGACCGACCTCAACACAGTCTGACTTCATCAATGTTACG  534
                |||  | |  || || | | | | | | || |||| |||  ||||| | ||||| ||||||
Sbjct  1567   GACT--GTTCCTCGTTTAGCCCCAACAACTTCAA-ACATCCTGACCTGATCAAGGTTACG  1511

Query  535    TGGAAACTGATGTGCGAAAGAGCATAACACACAATTTCAGCGTTTCTCGATCTCAACCGA  594
              |   | |   | | | |||| ||||| | |||||  |||||||||| |||||||||||
Sbjct  1510   TCAGAGCCATTATCCAAAAGGGCATACCCCACAACCTCAGCGTTTCCCGATCTCAACCTC  1451

Query  595    ACGGGAATCGTTC  607
              ||||||||| |||
Sbjct  1450   ACGGGAATCATTC  1438


Query: W4.2_SM_V7_W004_1206bp Query ID: lcl|Query_25508 Length: 1206

>Schistosoma mansoni LTR-retrotransposon Boudicca, partial sequence
Sequence ID: AY308024.1 Length: 1722
Range 1: 512 to 555

Score:58.1 bits(63), Expect:0.001,
Identities:39/44(89%),  Gaps:0/44(0%), Strand: Plus/Plus

Query  346  GCAACGATAAATGTACTAATATCGAACTAGACCATAATACTACA  389
            |||| |||||||| | ||||||| |||||||||||||| |||||
Sbjct  512  GCAAAGATAAATGCAATAATATCAAACTAGACCATAATTCTACA  555


Query: W5.2_SM_V7_W018_1104bp Query ID: lcl|Query_25509 Length: 1104

>TPA_inf: Schistosoma mansoni Perere-2 non-LTR retrotransposon
Sequence ID: BN000793.1 Length: 4544
Range 1: 3467 to 3586

Score:109 bits(120), Expect:2e-19,
Identities:96/120(80%),  Gaps:0/120(0%), Strand: Plus/Plus

Query  983    atcatcatcGTTCTTGCTATATGCTGACTATGTGAAGATATGGAGAGCGATATGAAGTGA  1042
              ||| |||||| |||| || ||||||||  |||| |||||||||||||||||   |||   |
Sbjct  3467   ATCGTCATCGGTCTTACTCTATGCTGATGATGTCAAGATATGGAGAGCGATACAAAGCAA  3526
```

```
Query  1043  GGGTGGTAGCTGAGAACTCACAAATGACCAGAAGAAATTATCTGGATGGTTCCAAACTTG  1102
||| | ||||| ||||||   ||||||||   ||| |||||||| ||||| |||||| ||
Sbjct  3527  GGGCGATAGCTTAGAACTTCAAAATGACCTAGAGAGATTATCTGAATGGTCCCAAACCTG  3586


Query: W11.2_2_SM_V7_ZW_1294bp Query ID: lcl|Query_25510 Length: 1294

>Schistosoma mansoni clone F04 Perere 3 retrotransposon mRNA, partial sequence
Sequence ID: AY838777.1 Length: 451
Range 1: 285 to 432
Score:152 bits(168), Expect:2e-32,
Identities:126/150(84%),  Gaps:3/150(2%), Strand: Plus/Plus

Query  20   GAGATTCTGATTGGCTGATTAATTCACTGCTACTATGTTTAGCATTATTCTAGAATTTTC  79
            ||| ||||||||||||||||| | ||||||||||| ||| || |  ||||  ||||||||
Sbjct  285  GAGTTTCTGATTGGCTGATTACTACACTGCTACTACGTTGAGTAGAATTCCCGAATTTTC  344

Query  80   AGTAAAACGCAAGGACT-TTAAAATACTATAAAAGTCCTATATTTTCTGTACATCAAACG  138
            || ||||| |||||||| ||||| ||||||||||  ||||||||||||||||||  || |
Sbjct  345  AGGAAAACCCAAGGACTGTTAAAGTACTATAAAAACCCTATATTTTCTGTACAT-GAATG  403

Query  139  AACCTTTTGAAGTGAAGTGCTTCTCGCATA  168
||||  ||| ||| ||| ||||||||||||
Sbjct  404  AACC-ATTGGAGTAAAGGGCTTCTCGCATA  432

Query: W16.2_SM_V7_W007_317bp Query ID: lcl|Query_25511 Length: 317

>TPA_exp: Schistosoma mansoni transposon Curupira-1
Sequence ID: BN001525.1 Length: 4878
Range 1: 2706 to 2995

Score:302 bits(334), Expect:2e-77,
Identities: 252/313(84%),Gaps:23/313(7%),Strand: Plus/Plus

Query  1     TATTGTGAAGATCAAATCAATAGACACTGATCAGTCATTACAATTCCATAATAATAAAGC  60
             |||||  ||| |||| | || ||||||||||| ||||| ||||||||||| |||||||||
Sbjct  2706  TATTGCTAAGGTCAAGTGAACAGACACTGATCGGTCATCACAATTCCATAGTAATAAAGC  2765

Query  61    TGTAAAATCATTTTCTCTACATTATTAATAATAAAGTGATCATCACTCACTTTATTATTT  120
             |||| |                  | ||||||     ||||||| ||||||||||||  |
Sbjct  2766  TGTAGA------------------TGAATAAT-----GATCATCGCTCACTTTATTACCT  2802

Query  121   AGTCAACGTCTTTTCAGGTTGTTCAAATTTAAGGTTTCAGTACTGGATTTCGTACAGAAA  180
              | ||||  ||||| ||||| |||||||||| ||||||||||   |||| ||||||||||
Sbjct  2803  TGCCAACAGCTTTTAAGGTTTTTCAAATTTACGGTTTCAGTAAACGATTCCGTACAGAAA  2862

Query  181   ACCACGTCATATAATGAACACTAATCTGTCAAACAAAAGTATATGCAAAAATGTGTACGA  240
             || |||||||||||| ||||||||| ||| |||||||||||||||| ||  |||||||||
Sbjct  2863  ACAACGTCATATAATAAACACTAATTTGTTAAACAAAAGTATATGCTAAGTTGTGTACGA  2922

Query  241   TCATTGCAATCTATTTTCACTTAGCGACCGTAGACGATTCTACTGAGATCAGTTCACTAG  300
             ||||| ||| || ||||||||||||||| |||| ||||| ||||||||||||||||||||
Sbjct  2923  TCATTTCAAACTCTTTTCACTTAGCGACGGTAGGCGATTGTACTGAGATCAGTTCACTAG  2982

Query  301   TACAACAAGATAA  313
|||||||||||||
Sbjct  2983  TACAACAAGATAA  2995


>Schistosoma mansoni repetitive DNA element t-2 of SM(alpha) family
Sequence ID: X15618.1 Length: 335
Range 1: 54 to 335
```

```
Score: 499 bits(270), Expect: 4e-137,
Identities: 278/282(99%,Gaps: 0/282(0%), Strand: Plus/Plus


Query  2     GAGTGGACAACGCGATGGCGTTTGAAGCGAAAGCTACTGGGTTCGAGTCCCAGAGTGAAC  61
             ||||||||||||||||||||||||||||||||||||||||||||||||||||||||||||
Sbjct  54    GAGTGGACAACGCGATGGCGTTTGAAGCGAAAGCTACTGGGTTCGAGTCCCAGAGTGAAC  113

Query  62    ATCAACACTGAGATGCAGGTACATCCAGCTGACCAGTCGGAAATAGGACGAAACGCGCGT  121
             ||||||||||||||||||||||||||| |||||||||||||||| |||||||||||||||
Sbjct  114   ATCAACACTGAGATGCAGGTACATCCAACTGACCAGTCGGAAATTGGACGAAACGCGCGT  173

Query  122   CCTGGATTCCACTGCTAGCCACCATCCATCTTTGCTTACCATGCTTGTGAATTTAGGCTA  181
             ||||||||||||||||||||||||||||||||||||||||||||||||||||||||||||
Sbjct  174   CCTGGATTCCACTGCTAGCCACCATCCATCTTTGCTTACCATGCTTGTGAATTTAGGCTA  233

Query  182   TATCGAGGCAATACGCACAGTATGCACATATGACAATTACAGACTGACCAGTTGCAGTCC  241
             ||||||||||||||||||||||||||||||||||||||||||||||||| ||||||||||
Sbjct  234   TATCGAGGCAATACGCACAGTATGCACATATGACAATTACAGACTGACCGGTTGCAGTCC  293

Query  242   TAAACACATCAATGGGAAGATCCAAACAAACAATACTAAGTA  283
             ||||||||||||| ||||||||||||||||||||||||||||
Sbjct  294   TAAACACATCAATAGGAAGATCCAAACAAACAATACTAAGTA  335
```

Suppl. Fig. 4: Results of BLASTn analyses including sequence alignments of W2.2, W4.2, W5.2, W11.2, W16.2, and W36.2_W001 with the LTR retrotransposonSaci-1 (DeMarco et al. 2004), the LTR retrotransposon Boudicca (Copeland et al. 2003), the non-LTR retrotransposons Perere-2 and Perere-3 (DeMarco et al. 2005), the DNA transposon Curupira-1 (4,878 bp) (Jacinto et al. 2011), and the SMalpha family of SINE-like retrotransposons (Ferbeyre et al. 1998), respectively.

Supplementary Figure 5.

Autosomal occurrence of W25.2 on chromosome 4

>SM_V7_4_19147661-19149984_reverse

ACAGGCGTAAGTAAGTAAGTAAATTGACATGGCTGTGAAACTTGAAGAAATATTGAATAATCTTAGGTTTTGATG
CACACTTTCATGTGTGATGACAGAAAGCAAGACTGATCATCTAGTTACTATATATTACTTACCATTCCATTGTTG
AGCCACTCGCATCTTGCAAAATGACTCAAACAGTGTGTTGTCAATTGATCCAATGAATGAAATCCAAGGCATGAC
ACTTGCTGTGATGATTTCAGTCGAAGTATTCAAAGTCTTTTGATACACGAATGAGTTGACTGAAATCCATGTGTT
TCCCAAATCCTTGCTCATTATTGCACCTCCTCCATTCGATCCCAAACCGAACAATATGCCAAGTGGATTTGTGTA
TGCTATGATTTGTTCAACAGATGGTCCAACATCTGAGAATCGAGCAGAAATCAGTAGAAATATATTTTACATTAT
CTTGTCAAAATAAGCTTAAACAAAATTTTCGATTAATCAAAGTTGTTTGTAACTATGTATTTTATCAATCTGTTC
AATGAACAGTTAATATACTAAAAGTTTGTATTTACTACTTATTAAAATGCTTTATTTGTCCTAGACGAAAACGAA
ATCTCTTATGCCAGTCAGTTCAAGTTTTCTTGTATAATTAATAGTAAAATATGAAGAGAAGGAATTGTAAATGCT
GTTTTCAAGTACAAATTCTGCCACAATTAGCTTGTGGTGTTGAACTTGAATGTAAAACATTTGTTCTCCTTCATT
ATGATAATGTTTGATTTTATACATCATGTCTTGTAAACTGAGTCAGATATTCAGTCAATAGATTCATGAATAATC
ATTTTTGATAATTGTTATTCTATTAATTTCTAATGTCAACTAACTAAGGTTGATTAGCACATCATGAATGTGGTT
AAAATGGGCAATGAGTAGAATCGAACAGAGGTGGTACTGGATACTCTCCTGTTACAAGGTAGTAAGCTATAAACA
TCAAAATGTTC**GTGGATGCGCACTG**CTGAAGAGTCCCATAATAGGAAGAAACGGCCGTCCAGTGTTTCCAGGTTT
TCCATGGTGGTCTAGCTTCAATTGACTCATGCTTTCTACTATGAAAAATACTAAATCTCCACAAAACCCCTTCTG
ATAATTAATATAATCATATGCTCACTAGTGACTTCGAGAAGTATATCTAGGAGCTCTAGTGAGAAGCAGTGACCA
GTGGAGTTCAAAACCACGTCTGTTGTGAGATAGGAACTCACTGAAGACAATTGGTGAATGGTTGCTCAACTTCGT
GGATCAGTTGAAGTTAGACATTAACACCGTTGGATGCCAGCTCAGTGGTCTATCGGTTAAGGGCTCTAGCTCCAG
ACTGGTAGGTCCTGGGTTCGAATCTCGCGGGTGCGGATC**GTGGATGCACACTG**CTGAGGAGTCCCATAATTGTT
ATTCTATTAATTTCTAATCTAATGTCAACTAACTAAGGTTGTTTAGCACATCATGAATGTGGTTAAAATGGGGCA
ATGAGTAGAATCGAACAGCGGTGGTACTGGATACTCTCCTGTTACAAGGTAGTAAGCTCTGAACATCAAAAGGTT
GATTATTCTGAAGAAATTACTATACTTGTCGAATAATATAAATCTTGGTTGTGTGTGTGCTACCGAAGTCGATAG
ATATAAGTAGTATGTGTCATCAATCGAATGTGACATGCCTGGAAGTAGAAAGTTAAGAAGATCGAAGAAAAAAGA
GAACAAGAACAGAACGATTGGTGTGGAAACGAAGGAACAATCAAGTGTGAGACGATTGATTGACATTTCGTAAAT
GAAGTATTCAGTTTATGGTTCCCAGATTTTACTAAAATATTCTGTAATTTTGTATTGTAATGCATTTGGTTGTAC
CCTCTGGTGTTCTCGTTCACTACATTTGAACTAAATAGGTTTGCATTGAGGGTATATTCAGTATTGATTTGAACA
TACGTTTATTTGTTATTGGGATTTCTATAGTTTTATAAAATGGAGAAAGAAATTGTTTGTAATTAAAATAAAAGG
TAACTTTAATAAGCATCCAATTAACTTTAAGATTCGGATGACTTCATTTGTCTAGTTTACTCGTTCTAATATTCT
CATTAATTACTGTTGAATTATGTAGAGATATTGTATTGTGTACAATACACACCCTAACTAAACACAAAGATGGAG
TTAACTGTGCTACATTGGTGTACATTTAACTGTCATAGTTAACACGATTATTCAATTGAATATTAAATATTCAAA
CCATATCGAAATCAACACCGAACATTCTAATTCTGATAAATGTTAGTATAATTTGATTAGTCGTTTTGTACTG

Suppl. Fig. 5: Representative example of a WE occurring on an autosome. In this case, a W25.2 element on chromosome 4 (SM_V7_4_19147661-19149984) is shown flanked by autosomal sequences. The W25.2 element is 420 bp long (highlighted in yellow). At its 5´end, a 14 bp long sequence (GTGGATGCGCACTG; **bold letters**) occurs that is repeated near the 3´end of this W25.2 element with one base difference (GTGGATGCACACTG; underlined). In close vicinity of this W25.2 element, another repeated sequence area of about 150 bp exists (underlined) that is part of the flanking autosomal region. Although there is no complete sequence identity (identical bases are highlighted in grey), the sequence similarity is obvious. Bases marked in red represent the ribozyme sequence, which is part of W-chromosomal and autosomal variants of W25.2 (see text and Supplementary Figure 12).

Supplementary Figure 6.

Sample-distance matrix analysis shows WE transcript occurrence across all analysed samples

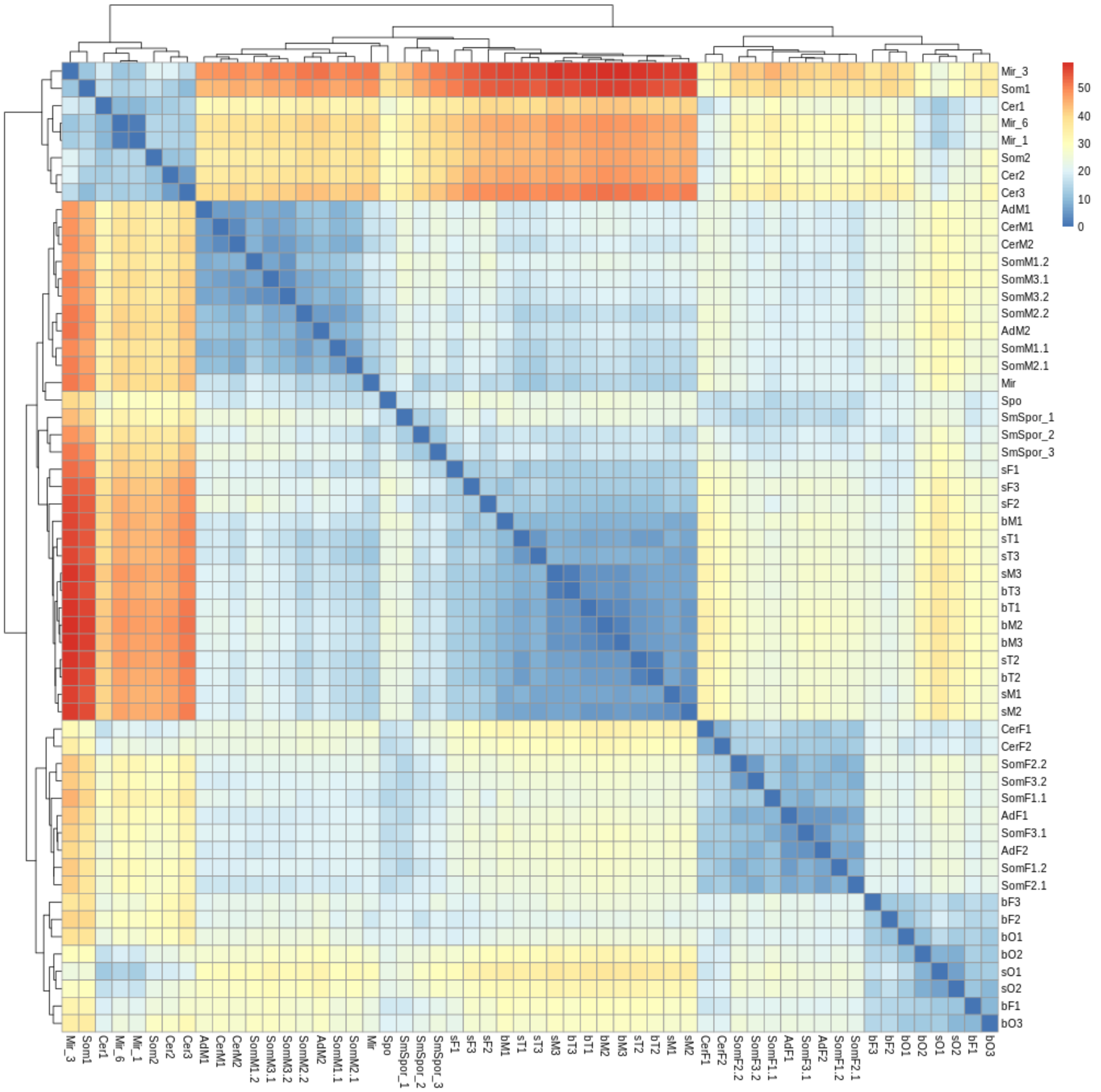

Suppl. Fig. 6: Sample-distance matrix analysis showing differences of transcript occurrence of all WEF among all samples. Instead of indicating individual WEF transcript levels, in this first overview the occurrence of transcripts of all WEFs present in a single sample is shown in comparison to all other samples analysed in this study. The orange/red color indicates high differences in transcript amounts of all WEFs added together versus high congruence in transcript amounts given in light/dark blue. A value of complete congruence (100%) is only achieved when a sample is compared to itself (see dark blue squares of the diagonal). E.g., among the biggest differences is Mir_3 (the third biological replicate of miracidia from the Liberian strain, Supplementary Table 1) compared sM2 (the second biological replicate of unpaired males from the Liberian strain). Further miracidial samples (Mir_1, Mir_6) from this strain showed a similar tendency, also to other samples of unpaired males (sM1, sM3). High

congruence in transcript amounts, for example, occurs in schistosomula (SomF and SomM samples) and samples from males and testes (sM1-sM3; bM1-sM3; sT1-sT3, bT1-bT3). The biological samples covered the following schistosome life stages: Mir, miracidia; Cer, cercariae; (Sm)Spo(r), sporocysts; Cer(M/F), cercariae (male/female); Som(F/M), schistosomula (female/male); AdM, adult paired males; sF, unpaired (single-sex) females, bF, paired (bisex) females; sM, unpaired (single-sex) males; bM, paired (bisex) males; sO, ovaries of unpaired females; bO, ovaries of paired females; sT, testes of unpaired males; bT, testes of paired males. When available, biological and technical replicates were included. The first number behind a sample abbreviation indicates the number of the biological replicate. The second number indicates the technical replicate. For example, SomF1.1 indicates the first biological and first technical replicate of a female schistosomula sample. SomF1.2 is the second technical replicate of this schistosomula sample. Samples without number had no replicate (see also Supplementary Table 1). Sample distance relations are given at the outer left side, and at the top.

Supplementary Figure 7: **Log_2 values of WEF**

See separate Excel file. These data were generated by a normalization approach in the form of log2-transformed normalized counts of all data sets.

Supplementary Figure 8.

Comparison of WEF transcript profiles between the Puerto Rican and the Guadeloupe strains of *S. mansoni*

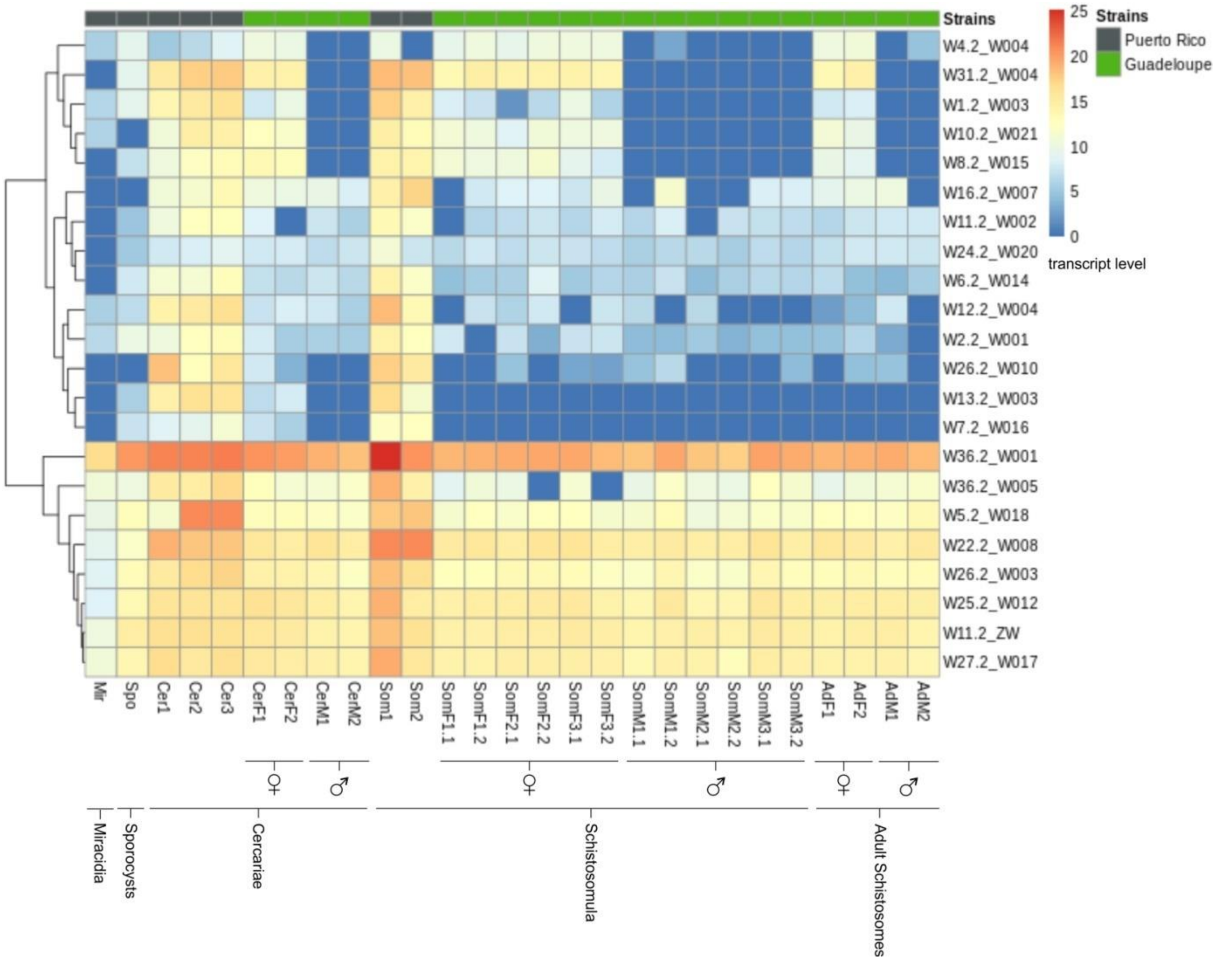

Suppl. Fig. 8: Sample-distance matrix analysis showing a quantitative analysis of the transcript amounts of all transcribed WEFs among samples from the Puerto Rican and the Guadeloupe strains. The biological samples covered the following schistosome life stages: Mir, miracidia; Spo, sporocysts; Cer, cercariae; Cer(M/F), cercariae (male/female); Som(F/M), schistosomula (female/male); AdM, adult paired males. When available, biological and technical replicates were included (see Supplementary Figure 5 and Figure 2). Biological symbols were used to indicate female and male samples. In cases without symbol, the sample origin was mixed-sex. The horizontal line at the top of this Figure shows a color code for the two different schistosome strains, Puerto Rico (grey) and Guadeloupe (green). Sample distance relations are given on the outer left side, and the appropriate WEs/WEFs on the outer right side. The color code in each horizontal row indicates relative transcript levels (from dark blue = no transcripts to deep red = high transcript level) based on log2-transformed normalized counts of all data sets.

Supplementary Figure 9.

WEF transcript profiles among all samples of the Liberian strain of *S. mansoni*

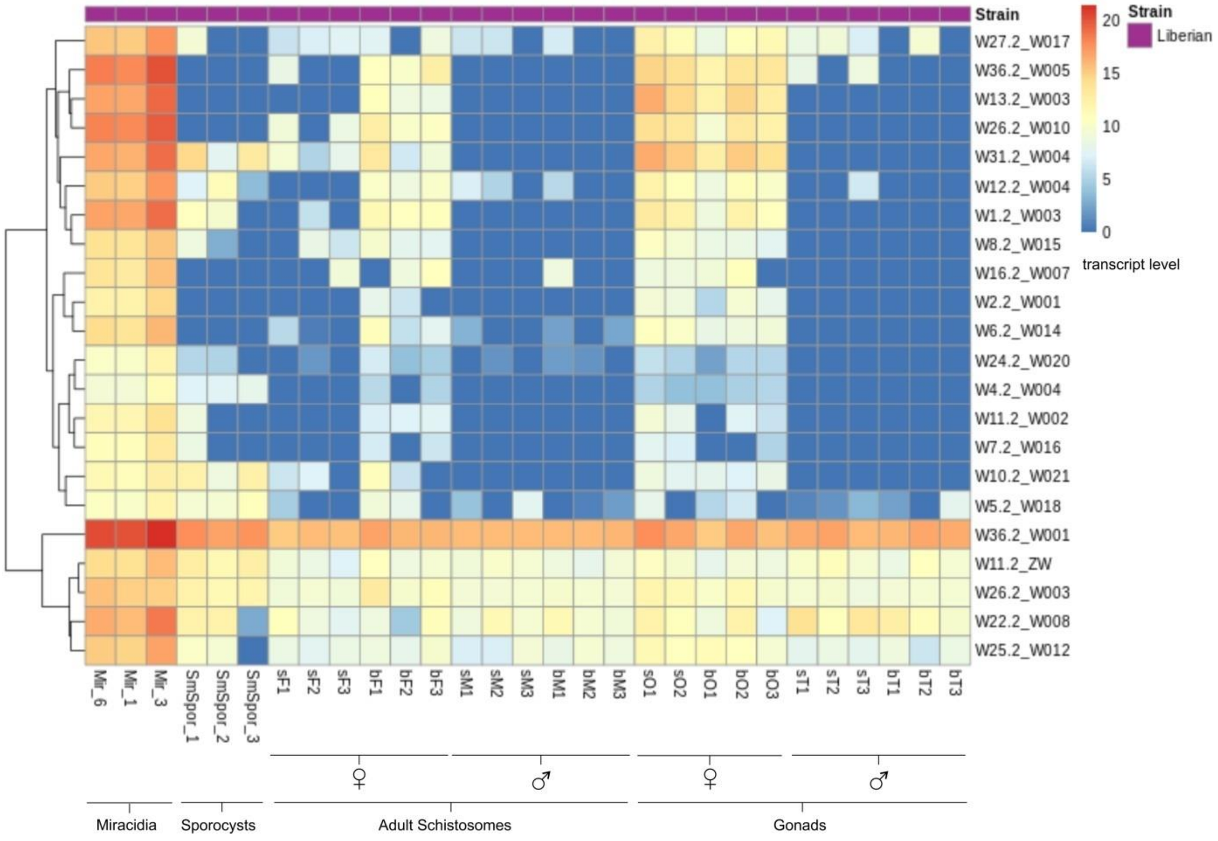

Suppl. Fig. 9: Sample-distance matrix analysis showing a quantitative analysis of the transcript amounts of all transcribed WEFs within the Liberian strain. The biological samples covered the following schistosome life stages: Mir, miracidia; SmSpor, sporocysts; sF, unpaired (single-sex) females, bF, paired (bisex) females; sM, unpaired (single-sex) males; bM, paired (bisex) males; sO, ovaries of unpaired females; bO, ovaries of paired females; sT, testes of unpaired males; bT, testes of paired males. When available, biological replicates were included, see Suppl. Fig. 5 and Figure 2. Biological symbols were used to indicate female and male samples. In cases without symbol, the sample origin was mixed-sex. The horizontal line at the top of this Figure shows thecolor code for the Liberian strain (purple). Sample distance relations are given on the outer left side, and the appropriate WEs/WEFs on the outer right side of the Figure. The color code indicates relative transcript levels (from dark blue = no transcripts to deep red = high transcript level) based on log2-transformed normalized counts of all data sets.

Supplementary Figure 10.

miRNA and snoRNA motifs within exemplary WEFs

A

W1.2

TCATTCAACAACATATAATTCTTTCTTCACACATATCACTGATCGAATGTCTTTGGAATATTTTGGAGTGAAATT
TGCGTTTCTCATTCTTATGAGCTTGATGACTCATGTGACAGGAATAAGATTTATGTGGATATCGACTGTGACAAT
GAGAATTGTGAATCGGATGTGCAGATGAGTTGTGCTGCGTACTTGTGTAGAGTTTCACAAATGTGTGATAATGCC
AATTCGAGTGTTTGTGGTTGCAATGACGTTCACACCTGGATTGAAGAGGCAGTGAGCGAATGTGATGATGCATTT
GATTGTGTGGTTGTGTTGGACCAATGTGCACATGGAATCGTTGCTTGTGCACATGGACCACCACAAATAACACAC
TCAATTCATTCTCCGTCTTCTCGAACAGGCATTGCCTACGAATCAAAAAAACTGTTTGCCTTATCATCTCGAACT
TTAATTTTTATTTCGAGAGTTAAAT

B

W22.2

ACTCACTCACTCACGCACCCACTCAATCACATCATTCACATATCTGTTCATTGTGAATGATTGGTATTCACGTTG
GTAGTAGTTGCGAAGATGTGCAGATCAGACACACAGACAGATGAACAGATCTCGTACAATCTGTGTGCTTCGAAT
GATGTCGATTTCACTACTACATCAATGAAGGATCCACTTGTAATGAGACGTTGAATAAACGAACGACTCATTGTG
CAGCCACATTAGTTCACTTGTTGTTTGTGTAGGAGTTTCGGCATCAATTCACGCTGTTCTCATTGCACAATGCGT
ATGTGTCAACACACCTCACATATGTGATTTTCATCAGTGACAATGCTTCCTACAACACAAATATCTCATTCATGT
CTACTCATATTCACACGCTCGAACATTCGCTCATTTACTCAACATTGACAAACTCACTGACTCAATCATTCATCC
ACTCACTCACTGAATCACTCACACAATCAACAAGTCACTCACTCTCCCACTCACTCACTCACTCACTCACTCACT
CACTCACTCACTCACACTCACTCACTTAATCACTCACTCACTCACTCACTCACTGATTCACACACTCACTCACTC
AATC

C

W5.2

GGGTGCTGATAAATACTTCAAGTGCTTTGTCATGCATATTCATCAACAAGGTTCACATTCATACACCATGAATAA
CGTTGAGCTACCTGTTGTCCAGCCACATAATGGCCTGTGAGTCATCGTTGACCAAGACGTGAGAAATACTCAAAG
CTTCTGTATGATAGCCAACAAAGGTTCCACAATCTTCCAACCGTGTTGTTGTGAAAGCATTTTTAACTTTCTACA
TCGTGTTTGTTCGTCCTAAACCCGAGTAATGCACACAGGTGGCTGTCGCCTGTCCAAAATGATACAGTGAGCATT
TGCAAATGTTTCATGGAACCATCACTTAGCTGATTCTCGAGATCCACAAGCTCGCGTACGATGATAGACTGGCTG
AACTAATCCTATTTTCCTTGTCATAAAATAGGTGCACAAGTGACTTGATCACAGTTTCCAATTTCATTAATGATA
ACTTTGCATCTGACATGTCCTCATATTATTATTATTATGATTACTGTCATTATGATTATTCGTATTCTCAAACAG
AATTCACGAGGACACTGAGAATTATCACATGCACAGAACAAATCACTTATCACTTCACCATCAAATCATCGAATT
TTCACTCAGTGGAATCCATCATCTCACCACTCACGTGAAGCTCTGTCTGTCCACATTTTCACATCAATTTAGATC
ATTTGAGAGACTGGCATCGCTAGCACTAACGAAGGTCGCTCAGACTCCCGTCCTCTCCATACTCAAACCGCGTCT
CCCCTCTTCAGTCTCCTCACTGCATTTCAACGTGTCACGGCTCAAGTAGCATTTCATGTAGAGTTAACTGAGTCT
GTCCTATGTTTCCATTCTGATCTTGGTTCGACTTTCATGGTGTGCGCTCTCCCTTGTGATTAGTCAGAGAAACCG
TCATAGGGACAGTGTTATCCCTCTCATATATAAATCACCATCCTCGTCTGGCATCCTCATCATCATCATCATCAT
CATCATCATCATCATCGTTCTTGCTATATGCTGACTATGTGAAGATATGGAGAGCGATATGAAGTGAGGGTGGTA
GCTGAGAACTCACAAATGACCAGAAGAAATTATCTGGATGGTTCCAAACTTGAC

D

W11.2

AAATGAAAAGTCACATGTGGAGATTCTGATTGGCTGATTAATTCACTGCTACTATGTTTAGCATTATTCTAGAAT
TTTCAGTAAAACGCAAGGACTTTAAAATACTATAAAAGTCCTATATTTTCTGTACATCAAACGAACCTTTTGAAG
TGAAGTGCTTCTCGCATATTTGTGCCTTTTCTCTCGTGTTCAAGTCGCTGTGTAGTTCTAGCTTGGGGGTCACGA
AAGTAGCTTAGGATCCGAATAATAGCGTTCAACCAACAAGACGTATCAGCGTATGAACCCGTGTTGGTCAACGAC

TACCATGGGACTGTATCGTCTAACGTTGCTCCACTGCCTTGTGGATCAGACCTTCACATCAAAGGCTCGTGGTGT
GGCCCCCTAAGAAAACCACCTGCTTCGGTTTCACGCACGAACACTATTCCAGTCCTCAGACAAATCCAATCACAA
TGTGTGCTGCATATACATTTGGTGCCTCCTTGTACCAATCTTCATGTTTTCTAGTAAATAAATACGGCACCTCTT
ATTTTAACCTCAAAATGACCAATCATTTCACATGTCGGCTCGACTACGAGTCACAGAGCATTCTCAATGCAGTCC
ATAATGTCAAATGATCTAGATGTAATTTGAGAAGTTCAATTTAGTTTTCTCAACAGTCTTCTCATTTCACTGAAC
AAAATTTCAATATTTCTCTTGTCTCATGAAATCAACATCTAAAACGCACTGCTCAGTTGTATTTCAGATGAACAA
TTTATGGTTGGATATTGATTGTAGAGACTATGAAATTCTTCACAACTGTGTGCCTCGCATTATAGTGGTTTCATT
TAAGTTATGCTGTTTGACAGATATTTAGGTAAAAATGAGCTATTATGGAATACAACACTACTGAATGATGATTCG
TGCTATAAAATGTTCAGTCGGTATAGTGAAATAGTTGAAACAGAGATACTATCTACGAGTTTTCTCTACAGTTTT
ATAGTGACTACCCGTAGAATGGAAGACACGCGTTTCGTAGTATTTGGGAGTCGTCATATGCCACCATTGTCCAAA
CGGAATGATCAATTGAAGTGGTTATCATTAACGACGAGGAGATTCAAACGTTTACAGATCCAAGGCACACTGCAT
GTTCAAAACGTGAGGTGCAAGTGTTACAATATAACTGTTGAGGGAAGAAATGGCGTATGTTGGTATGATCCTGAA
AATTTCTCGCAATCGACTTGATAAGCTCATGAAACATTGAGGAGCATTTCATTCAATCAGCGTTTGATTGGAAGT
TATGTTAGTAACATAGCGACACGCACGAACACTATTCCAGTCCTCAGACAAATCCAATCACAATGTGTGCTGCAT
ATACATTTGGTGCCTCCTTGTACCAATCTTCATGTTTTCTAGTAAATAAATACGGCACCTCTTATTTTAACCTCA
AAATGACCAATCATTTCACATGTCGGCTCGACTACGAGTCACAGAGCATTCTCAATGCAGTCCATAATGTCAAAT
GATCTAGATGTAATTTGAGAAGTTCAATTTAGTTTTCTCAACAGTCTTCTCATTTCACTGAACAAAATTTCAATA
TTTCTCTTGTCTCATGAAATCAACATCTAAAACGCACTGCTCAGTTGTATTTCAGATGAACAATTTATGGTTGGA
TATTGATTGTAGAGACTATGAAATTCTTCACAACTGTGTGCCTCGCATTATAGTGGTTTCATTTAAGTTATGCTG
TTTGACAGATATTTAGGTAAAAATGAGCTATTATGGAATACAACACTACTGAATGATGATTCGTGCTATAAAATG
TTCAGTCGGTATAGTGAAATAGTTGAAACAGAGATACTATCTACGAGTTTTCTCTACAGTTTTATAGTGACTACC
CGTAGAATGGAAGACACGCGTTTCGTAGTATTTGGGAGTCGTCATATGCCACCATTGTCCAAACGGAATGATCAA
TTGAAGTGGTTATCATTAACGACGAGGAGATTCAAACGTTTACAGATCCAAGGCACACTGCATGTTCAAAACGTG
AGGTGCAAGTGTTACAATATAACTGTTGAGGGAAGAAATGGCGTATGTTGGTATGATCCTGAAAATTTCTCGCAA
TCGACTTGATAAGCTCATGAAACATTGAGGAGCATTTCATTCAATCAGCGTTTGATTGGAAGTTATGTTAGTAAC
ATAGCGA

Suppl. Fig. 10: Shown are sequences of the WEFs W1.2 (**A**), W22.2 (**B**), W5.2 (**C**), and W11.2 (**D**). According to StructRNAFinder analysis, W1.2 (**A**) contains sequence parts representing the miRNAs mir-2587 (underlined) and mir-279 (green letters), which partly overlap. W22.2 (**B**) contains the snoRNA sequences sR11 (green background), snoZ178 (green letters), and SCARNA7 (underlined). W5.2 (**C**) contains sequence parts representing the miRNA mir-589 (underlined) and the snoRNA sR36 (green letters). W11.2 (**D**) contains sequence parts representing the miRNA mir-232 (underlined) and the snoRNA DdR16 (green letters), which partly overlap.

Supplementary Figure 11.

Putative secondary structures of the predicted miRNAs and snoRNAs

See separate Excel file.

Supplementary Figure 12.

SNORD 59 is a predicted sequence part of WEF W2.2

A

```
                                         Alignments:
                          v                                                              v  NC
                         <<<-<_______________________________________________~~~~~~>->>> CS
           RF00273    1 cCUUCaaUGAUGAuUuAcauuuUGACUUUCGUUCUUCUGAGUUUGCUGAAGC*[16]*GAAGg 73
                         : U C+AUGAUGA      ++++UGA UU C  UCUUC GAGU UG UG AGC      GAA :
SM_V7_W001_422658-47 112 UCUACUAUGAUGAA----CAACUGAUUUGCUAUCUUCCGAGUAUGAUGCAGC*[ 3]*GAAAA 167
                         ************75....5789**************************9987...8..***** PP
```

B

>W2.2_SM_V7_W001_709bp
AATGTCACGTGGAAACTGATGTCCGAAAGAGCATAACACACAATTTCAGCGTTTCCCGATCTCAACCGAACGGGA
ATCGTTCGTGAACACACTTCACTGTTTATTGATTGAGGAAATGCGCAACGATTCTCGGCACTCGGCTCATCATTT
GAGCTGTTGACTGTTCAATCGTTGTGCATGATTGTCTTCTACTCATACTGATTGATCACTTGAATAGACATCGGC
AAACTGCACATCAGCAAGCTTACGCACTTGGTCGTCCAACGTGTGTACTCCTCTACTATGATGAACAACTGATTT
GCTATCTTCCGAGTATGATGCAGGTCCGAAAACAGCCCACCACAACAACGTTCTGACGGCGTACGCGTTTCGCCT
TACACACATTTCGTGGCCTGACACCCAACGTTCTTCCGACACGTCACAACCAATCACCAGTAGAACCTCTCCACA
ATCTACATCATCTAATTGTACATCACACAGATGCGGTCAATTCACCAGGTTGTTCGTTATCAACTTTGTTGACTT
ATGCACTCATACACATGCACAATCACAGCTCCTTCGATCATCACTTGTTCAGACCAATATGCAGAACATAGCTCA
ATAGACGCATTCGTAATTTTCGTCGTTCTATTACCACACACAGTGTGCATTTCCACCGACACTTAGATTTCCTTC
AACCCGACCGACCTCAACACAGTCTCACTTCATC

>W2.2_SM_V7_W001_711bp
TCTTCTACTGATACTGATTTATCACTTGAATAGACATCGGCAAACTGCACATCAGCAAGCTCACGCACTTGGTCG
TCCAACGTGTGTACTCCTCTACTATGATGAACAACTCATTTGCTATCTTCCGAGTGTGGTGCAGCTCCGAAAACA
GACCACCACAACAACGTTCTGACGGCGTACGCGATTCGCCTTACACACATTTGGTCGCCTCACACCCAATGTTCT
TCCGACACGTCGCAACCAATCAGCAGTAGAACCTCTCCACAATCTACATTATCTAATTCTACATCACACAGATGC
GGTCAATTCACCAGGTTGTTCGTGATCAACTTTGTTGACTTATGCACTCATACACATGCACAATCACAGCTCCTT
CGATCATCACTTGTTCACACCAATATGCAGAACATAGCTCAATAGGCGCATTCGTAATTTTCCTCGTTCTATTAC
CACACACAGTGTGCATTTCCACCGACACTTAGATTTCCTTCAACACGACCGACCTCAACACAGTCTGACTTCATC
AATGTTACGTGGAAACTGATGTGCGAAAGAGCATAACACACAATTTCAGCGTTTCTCGATCTCAACCGAACGGGA
ATCGTTCGTGAACACACACTTCACTGTTTAGTGATTAAGGAAATGCGCAACGATTCTCGGCACTCGGCTCATCAT
TTCAGCTGTTGACTGTTGAATCGTTGTGCATGATTG

Suppl. Fig. 12: StructRNAFinder analysis detected SNORD 59 as part of WEF W2.2. **A**, alignment of the sequence of SNORD 59 (Rfam ID RF00273; http://rfam.xfam.org/) with the part of W2.2, which contains this snoRNA. **B**, two variants of W2.2 (709 bp and 711 bp), which show either complete congruence of the sequence (yellow background) including the C-box (UGAUGA; red) and D-box (CUGA; green) motifs in the 709 bp variant, or a mutated form in the 711 bp variant with a single G/C base mutation at position 3 of the D-box motif (grey).

Supplementary Figure 13.

Multiple sequence alignment of SMalpha-HHR and HHR-like sequences found within WEFs on autosomes

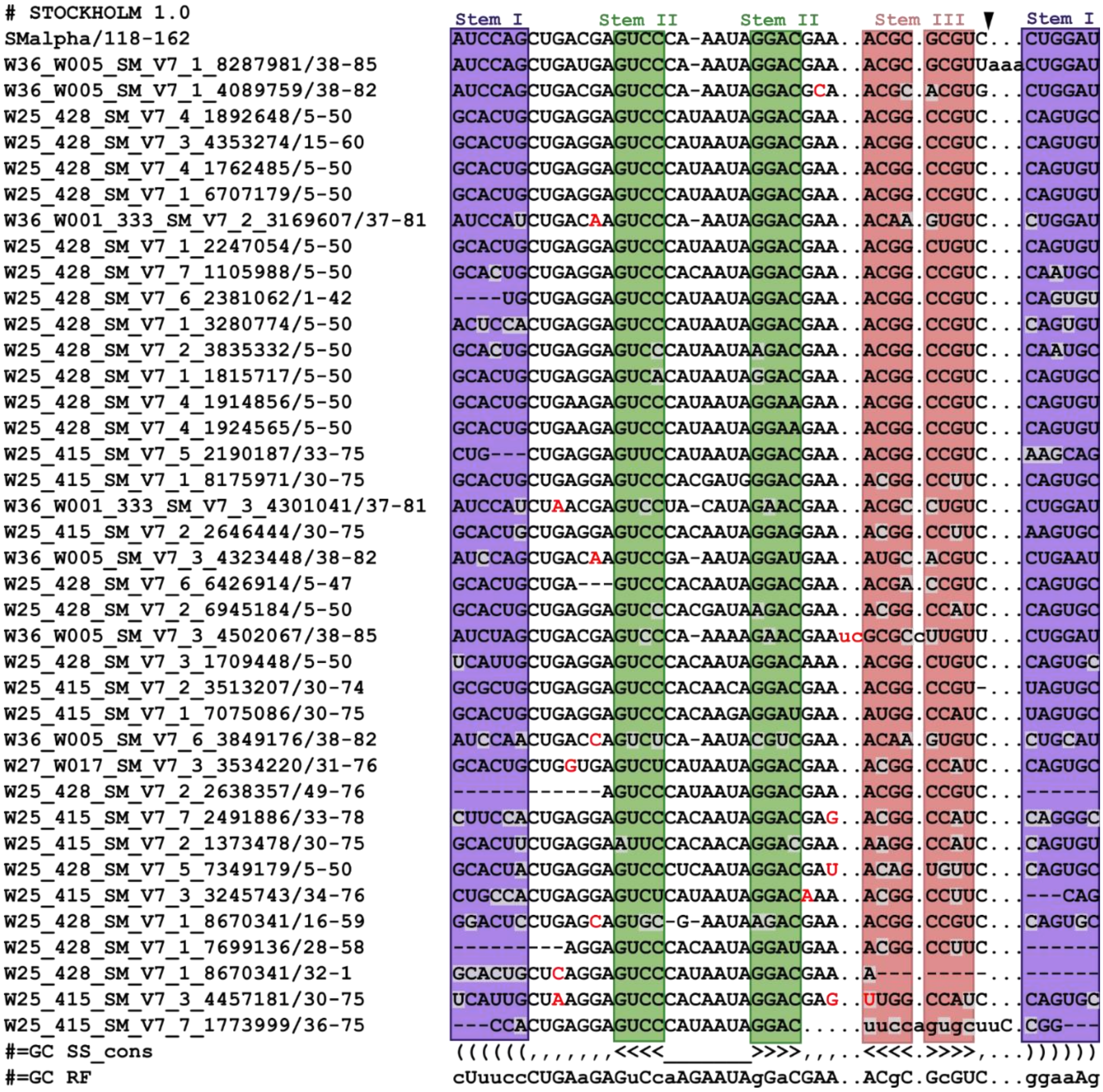

Suppl. Fig. 13: This alignment was generated using Infernal version 1.1.2 (Nawrocki et al. 2013). Conserved base-pairing interactions are indicated by the dot bracket annotation at the bottom of the alignment and additionally highlighted by colored boxes labelled at the top. The ribozyme cleavage site is indicated by an arrowhead. Nucleotide conservation over all analysed sequences is shown in the last row of the alignment. Nucleotides that differed from the consensus CUGANGA and GAAA sequences typical for HHRs are highlighted in red and nucleotides that do not take part in forming otherwise conserved secondary structures are in light grey boxes. WE sequences shown in this alignment correspond to examples listed in supplementary table 3.

II. Supplementary Table 1.

Samples and sources of *S. mansoni* used in this study

Type	Abbreviation	Project Accession	Sample Accession	Biol. Repl.	Reference	Strain
Unpaired female (single sex)	sF1; sF2; sF3	PRJEB 14695	ERR506091, ERR506092, ERR506093	3	Lu et al. (2016)	Liberia
Paired female (bisex)	bF1; bF2; bF3	PRJEB 14695	ERR506082, ERR506083, ERR506084	3	Lu et al. (2016)	Liberia
Ovary of sF	sO1; sO2	PRJEB 14695	ERR506071, ERR506072	2	Lu et al. (2016)	Liberia
Ovary of bF	bO1; bO2; bO3	PRJEB 14695	ERR506073, ERR506074, ERR506075	3	Lu et al. (2016)	Liberia
Female cercariae	CerF1; CerF2	PRJNA 312093	SRR3223434, SRR3223435	2	(Picard et al. 2016)	Guadeloupe
Female schistosomula stage 1	SomF1.1; SomF1.2	PRJNA 312093	SRR3223436, SRR3223439	2	(Picard et al. 2016)	Guadeloupe
Female schistosomula stage 2	SomF2.1; SomF2.2	PRJNA 312093	SRR3223443, SRR3223444	2	(Picard et al. 2016)	Guadeloupe
Female schistosomula stage 3	SomF3.1; SomF3.2	PRJNA 312093	SRR3223445, SRR3223446	2	(Picard et al. 2016)	Guadeloupe
Adult female	AdF1; AdF2	PRJNA 312093	SRR3223447, SRR3223448	2	(Picard et al. 2016)	Guadeloupe
Adult male before pairing (single sex)	sM1; sM2; sM3	PRJEB 14695	ERR506110, ERR506111, ERR506113	3	Lu et al. (2016)	Liberia
Adult male after pairing (bisex)	bM1; bM2; bM3	PRJEB 14695	ERR506076, ERR506088, ERR506090	3	Lu et al. (2016)	Liberia
Testis of sM	sT1; sT2; sT3	PRJEB 14695	ERR506077, ERR506078, ERR506085	3	Lu et al. (2016)	Liberia
Testis of bM	bT1; bT2; bT3	PRJEB 14695	ERR506079, ERR506080, ERR506081	3	Lu et al. (2016)	Liberia
Male cercariae	CerM1; CerM2	PRJNA 312093	SRR3211868, SRR3216389	2	(Picard et al. 2016)	Guadeloupe
Male schistosomula stage 1	SomM1.1; SomM1.2	PRJNA 312093	SRR3223426, SRR3223427	2	(Picard et al. 2016)	Guadeloupe
Male schistosomula stage 2	SomM2.1; SomM2.2	PRJNA 312093	SRR3223428, SRR3223429	2	(Picard et al. 2016)	Guadeloupe

Male schistosomula stage 3	SomM3.1; SomM3.2	PRJNA 312093	SRR3223430, SRR3223431	2	(Picard et al. 2016)	Guadeloupe
Adult male	AdM1; AdM2	PRJNA 312093	SRR3223432, SRR3223433	2	(Picard et al. 2016)	Guadeloupe
Miracidium	Mir	PRJNA 209511	SRR922067	1	Wang et al. (2013)	Puerto Rico
Sporocyst (in vitro 48h)	Spo	PRJNA 209511	SRR922068	1	Wang et al. (2013)	Puerto Rico
Cercaria	Cer1; Cer2; Cer3	PRJEB 2350	ERR022872, ERR022877, ERR022878	3	Protasio et al. (2012)	Puerto Rico
Schistosomulum (in vitro3h)	Som1; Som2	PRJEB 2350	ERR022876, ERR022879	2	Protasio et al. (2012)	Puerto Rico
Miracidia	Mir_6; Mir_1; Mir_3	PRJEB 15637	ERR1674583, ERR1674584, ERR1674585	3	published in ENA; source: Grevelding (1995)	Liberia
Sporocysts 18 weeks	SmSpor_1; SmSpor_2; SmSpor_3	PRJEB 15637	ERR1674590, ERR1674591, ERR1674592	3	published in ENA; source: Grevelding (1995)	Liberia

Suppl. Tab. 1: List of all sample types, strains, replicates (Biol. Repl.), accession numbers (projects and samples) and references used in this study. RNA-Seq data were obtained from ENA (http://www.ebi.ac.uk/ena). The data originated from different sources as indicated. Background color code indicates the different strains used and refers to the figures.

Supplementary Table 2.

miRNAs predicted to be parts of WEFs

miRNA	WEF	Rfam ID	Transcript occurrence
mir-2587	W1.2	RF01917	bF2, bF3, sO1, bO2, bO3, Cer1, Cer2, Cer3, Som2
mir-785	W12.2	RF02244	bF1, sO1, bO1, bO2, Cer1, Cer2, Cer3, Som1
mir-181	W7.2	RF00076	sO2, Spo, Cer3, Som1, Som2
mir-589	W5.2	RF01059	sF1, bF1, CerF1, CerF2, all SomF, AdF1, AdF2, sM1, sM3, bM2, bM3,sT1, sT2, sT3, bT1, bT3, CerM1, CerM2, all SomM AdM1, AdM2, Cer1
mir-232	W11.2_ZW	RF00856	bF2, sT3
mir-279	W1.2	RF00754	Spo
mir-891	W8.2	RF01042	Spo

Suppl. Tab. 2: Summary of StructRNAFinder results identifying potential miRNAs in WEFs. Given are the miRNA identifiers, the harboring WEFs, RFamIDs (Kalvari et al. 2018; https://rfam.xfam.org), and the stages and/or tissues in which the respective WEF/miRNA transcripts were detected. bM, bisex males; sM, single-sex males; bT, testes from bisex males; sT, testes from single-sex males; bF, bisex females; sF, single-sex females; bO, ovaries from bisex females; sO, ovaries from single-sex females; Cer, cercariae; CerM/F, male or female cercariae, respectively; Som, schistosomula; SomM/F, male or females schistosomula, respectively; Spo, sporocysts; numbers added to these abbreviations indicate biological replicates.

Supplementary Table 3.

SnoRNAs predicted as parts of WEFs

snoRNA	WEF	Rfam ID	Transcript occurrence
sR11	W22.2	RF01150	bM3, sT1, CerM1, SomM1.2, AdM2
DdR16	W11.2_ZW	RF01566	sM1, sM3, sT2
sR36	W5.2	RF01124	Mir, Spo, Som2
TB11Cs2H1	W26.2_W003	RF01537	bF3, bO1
SNORD59	W2.2	RF00273	bF1, bO2, Spo
GlsR19	W36.2_W001	RF02482	bO2
snoZ178	W22.2	RF00306	bM3
SNORD5	W24.2	RF01161	AdM2
SCARNA7	W22.2	RF01295	Cer1

Suppl. Tab. 3: Summary of StructRNAFinder results identifying potential snoRNAs in WEFs. Given are the snoRNA identifiers, the harboring WEF, RFamIDs (Kalvari et al. 2018; https://rfam.xfam.org), and the stages and/or tissues in which transcripts of these WEFs were identified.

Supplementary Table 4.

Prediction of self-cleavage activity of different HHR candidates found in WEFs in *S. mansoni*

WEF	Autosomal sequence	HHR	Predicted activity	Mutations or mismatches
W36_W001_333	SM_V7_3_4301041	Y	inactive	CU**G**ANGA
	SM_V7_2_3169607 (W36_333_1)	Y	slow or inactive	CUGAN**G**A
W36_W005	SM_V7_1_8287981	Y	active	
	SM_V7_1_4089759	Y	slow or inactive	G**A**AA
	SM_V7_3_4502067	Y	slower	GAA**A**
	SM_V7_6_3849176	Y	inactive	CUGAN**G**A
	SM_V7_3_4323448	Y	slow or inactive	CUGAN**G**A
W27_W017	SM_V7_3_3534220	Y	inactive	CUG**A**NGA
W26_402	SM_V7_4_2937271	N	no HHR	
	SM_V7_1_4421753	N	no HHR	
W26_401	SM_V7_4_2693603	N	no HHR	
	SM_V7_3_2126851	N	no HHR	
W26_400	SM_V7_2_4369464	Y	slow or inactive	CUGAN**G**A
W25_415	SM_V7_3_4457181	Y	inactive	CU**G**ANGA
	SM_V7_5_2190187	Y	active	
	SM_V7_2_3513207 (W24_415_3)	Y	inactive	Deletion in cleavage site
	SM_V7_2_1373478	Y	active	
	SM_V7_1_7075086 (W25_415_1)	Y	active	
	SM_V7_5_2462227	Y	active	
	SM_V7_2_2646444	Y	active	
	SM_V7_7_2491886	Y	inactive	GA**A**A
	SM_V7_1_8175971	Y	active	
	SM_V7_3_3245743	Y	slow or inactive	**G**AAA
	SM_V7_7_1773999	Y	inactive	GAAA is missing
W25_428	SM_V7_2_3835332	Y	active	
	SM_V7_7_1105988	Y	active	
	SM_V7_2_2638357	Y	no HHR	
	SM_V7_3_4353274	Y	active	
	SM_V7_6_2381062	Y	inactive	Stem I is missing
	SM_V7_3_1709448	Y	slow or inactive	**G**AAA
	SM_V7_1_2247054	Y	active	
	SM_V7_4_1762485	Y	active	
	SM_V7_1_1815717 (W25_428_10)	Y	active	Mismatch in Stem II
	SM_V7_1_3280774	Y	active	Mismatch in Stem I
	SM_V7_4_1914856 (W25_428_5)	Y	active	
	SM_V7_4_1924565	Y	active	
	SM_V7_1_6707179	Y	active	
	SM_V7_3_4008523	Y	slower	GAA**A**
	SM_V7_4_1892648	Y	active	
	SM_V7_2_6945184	Y	active	Mismatch in Stem III

	SM_V7_3_4545219	Y	inactive	GA**A**A
	SM_V7_1_7699136	Y	inactive	CUGANGA is missing
	SM_V7_6_6426914	Y	inactive	CUGAN**GA**
	SM_V7_1_8670341	Y	inactive	CUGANGA is missing
	SM_V7_4_3433275	Y	inactive	CUGANGA is missing
	SM_V7_6_5833222	Y	inactive	CUGANGA is missing
	SM_V7_5_7349179	Y	inactive	GA**A**A

Suppl. Tab. 4: Chromosome number and initial position of the WEs on the autosome are listed under autosomal sequence. We examined HHR sequences for the presence of highly conserved nucleotides in the ribozyme core and presence of the three-stem junction to determine whether the candidate is likely a HHR (Y) or not (N). Based on findings from Ruffner et al. (1990), we predicted HHR activity. We subjected the candidates highlighted in grey to co-transcriptional self-cleavage assays (Figure 4C).

Supplementary Table 5.

WEF occurrence in *S. mansoni, S. japonicum, S. haematobium, and S. rodhaini*

See separate Excel file.

Supplementary Table 6.

Oligonucleotides used in this study

Name	Sequence	Purpose
**CEW385: W25_428_5_for**	5'- GAAAT**TAATACGACTCACTA TAg**GTTCGTGGATGCGCACTGCTG AAGAGTCCC -3'	Forward primer to produce candidate W25_428_5 with **T7 promotor** together with the primer CEW386
**CEW386: W25_428_5_rev**	5'- GAAAACCTGGAAACACTGG ACGGCCGTTTCTTCCTATTATGGGA CTCTTCAGCAG -3'	Reverse primer to produce candidate W25_428_5 together with CEW385
**CEW387: W25_428_10_for**	5'- GAAAT**TAATACGACTCACTA TA**GGTTCGTGGATGCGCACTGCTG AGGAGTCAC -3'	Forward primer to produce candidate W25_428_10 with **T7 promotor** together with the primer CEW388
**CEW388: W25_428_10_rev**	5'- GAAAACCTGAAAGCACTGG ACGGCCGTTTCGTCCTATTATGTGA CTCCTCAGCAG-3'	Reverse primer to produce candidateW25_428_10 together with the primer CEW387
**CEW389: W36_333_1_for**	5'- GAAAT**TAATACGACTCACTA TAg**GAGATGCAGGTACATCCATCT GACAAGTCCC -3'	Forward primer to produce candidate W36_333_1 with **T7 promotor** together with the primer CEW390
**CEW390: W36_333_1_rev**	5'-GCTAGCAGTGGAATCCAGGA CACTTGTTTCGTCCTATTTGGGACT TGTCAGATGG-3'	Reverse primer to produce candidate W36_333_1 together with the primer CEW389
**CEW391: W25_415_1_for**	5'- GAAAT**TAATACGACTCACTA TAg**GATGGTGGATGCGCACTGCTG AGGAGTCC -3'	Forward primer to produce candidate W25_415_1 with **T7 promotor** together with the primer CEW392
**CEW392: W25_415_1_rev**	5'- GGAAAACCTGGAAGCACTA GATGGCCATTTCATCCTCTTGTGGG ACTCCTCAGCAG -3'	Reverse primer to produce candidate W25_415_1 together with the primer CEW391
**CEW393: W25_415_3_for1**	5'- GAAAT**TAATACGACTCACTA TAg**GAATCTCTCGATGCTGGATCGT TG -3'	Forward primer to produce candidate W25_415_3 with **T7 promotor** to extend HHR sequence with the primer CEW396
**CEW394: W25_415_3_for2**	5'- CGATGCTGGATCGTTGTTGC GCGCTGCTGAGGAGTCCCACAACA GGACGAAAC -3'	Forward primer to produce candidate W25_415_3 together with the primer CEW395
**CEW395: W25_415_3_rev1**	5'- CTAGACCACCATGAGAAACC TGAGAGCACTAACGGCCGTTTCGT CCTGTTGTGG -3'	Reverse primer to produce candidate W25_415_3 together with the primer CEW394

**CEW396: W25_415_3_rev2**	5'- GTATATGTAGTTGAGATCAT GAGCCAATTGAAGCTAGACCACCA TGAGAAAC -3'	Reverse primer to produce candidate W25_415_3 to extend HHR sequence with the primer CEW393
**CEW397: W25_415_3_rev3**	5'- GTATATGTAGTTGAGATCAT GAG-3'	Reverse primer to produce candidate W25_415_3 to amplify HHR with T7 promotor using the primer CEW47
**CEW398: W25_415_3_for2_R B mutant**	5'- CGATGCTGGATCGTTGTTGC GCGCTGCTCAGGAGTCCCACAACA GGACGAAAC -3'	Forward primer to produce the catalytically inactive variant of W25_415_3
**CEW404: W25_415_3_ short_for**	5'- GAAAT**TAATACGACTCACTA TAG**GATCGTTGTTGCGCGCTGCTG AGGAGTCC-3'	Forward primer to produce candidate W25_415_3 in short version with **T7 promotor** for *in vitro* transcription
**CEW405: W25_415_3_ short_rev**	5'- GAAACCTGAGAGCACTAAC GGCCGTTTCGTCCTGTTGTGGGAC TCCTCAGCAGC-3'	Reverse primer to produce candidate W25_415_3 in short version for *in vitro* transcription

Suppl. Tab. 6: Listed are the names, sequences, and purposes of the oligonucleotides designed in this study. Letter in bold represent T7-promoter sequences.
